# Comprehensive Profiling of *N* ^6^‐methyladnosine (m^6^A) Readouts Reveals Novel m^6^A Readers That Regulate Human Embryonic Stem Cell Differentiation

**DOI:** 10.1002/advs.202510075

**Published:** 2026-01-20

**Authors:** Zhou Huang, Rucong Liu, Zibaguli Wubulikasimu, Wanqing Zhao, Jiaqi Huang, Jiaxuan Wang, Tianyuan Zhang, Rui Fan, Wei Kong, Qinghua Cui, Yang Li, Yuan Zhou

**Affiliations:** ^1^ Department of Biomedical Informatics School of Basic Medical Sciences Peking University Beijing China; ^2^ State Key Laboratory of Vascular Homeostasis and Remodeling Peking University Beijing China; ^3^ Department of Cell Biology School of Basic Medical Sciences Peking University Stem Cell Research Center Peking University Beijing China; ^4^ Cancer Center Daping Hospital Army Medical University Chongqing China; ^5^ National Institute of Geriatrics National Health Commission Beijing Hospital Beijing China; ^6^ State Key Laboratory of Female Fertility Promotion Center for Reproductive Medicine Department of Obstetrics and Gynecology Peking University Third Hospital Beijing China; ^7^ Shenzhen Key Laboratory of Cardiovascular Disease Fuwai Hospital Chinese Academy of Medical Sciences Shenzhen China; ^8^ Department of Physiology and Pathophysiology School of Basic Medical Sciences Peking University Beijing China; ^9^ Department of Integrated Traditional Chinese and Western Medicine School of Basic Medical Sciences Peking University Beijing China

**Keywords:** epitranscriptomic regulations, m6A readers, m6A readouts, RNA modifications, stem cell differentiation

## Abstract

*N* ^6^‐methyladenosine (m^6^A) modification constitutes a crucial layer of post‐transcriptional regulations, but the landscape of its downstream readout effects remains less comprehensively understood. Therefore, we systematically assess the readout effects of m^6^A on mRNA half‐life, translation efficiency, and alternative splicing across five cell lines (A549, HEK293T, HUVEC, JURKAT, and human embryonic stem cells (hESCs)) using actinomycin D‐disrupted temporal transcriptome, ribosome sequencing, and ultra‐high‐depth transcriptome sequencing, respectively. Our analysis, coupled with the integration of public and newly profiled m^6^A methylome data, reveals high cell type specificity in m^6^A readouts where m^6^A level alone is insufficient to predict m^6^A readouts. Nonetheless, machine learning models focusing on RNA‐binding protein (RBP) binding context can effectively predict the readouts and prioritize four novel m^6^A‐associated proteins (FUBP3, FXR2, L1TD1, and DDX6). Their m^6^A‐binding ability is validated by m^6^A RNA pull‐down, transcriptome‐wide binding site mapping, and electrophoretic mobility shift assay, while FUBP3 and L1TD1 are further suggested as m^6^A readers regulating mRNA stability based on half‐life profiling of knockout cells. Finally, FUBP3, FXR2, and L1TD1 are demonstrated to regulate hESC differentiation without affecting self‐renewal. Together, this study bridges the gap in understanding m^6^A functional readouts and lays the groundwork for future research on m^6^A‐mediated stem cell fate decisions.

## Graphical abstract

This research deciphers the m^6^A transcriptome by profiling its sites and functional readout effects: from mRNA stability, translation to alternative splicing, across five different cell types. Machine learning model identifies novel m^6^A‐binding proteins DDX6 and FXR2 and novel m^6^A reader proteins FUBP3 and L1TD1. Further experimental investigation reveals their new regulatory role in human embryonic stem cell differentiation.

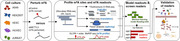

## 1 Introduction

The mammalian transcriptome is extensively regulated by a variety of chemical modifications, among which *N* ^6^‐methyladenosine (m^6^A) has been proven as one of the most prevalent modifications [1, 2, 3]. The on‐and‐off state and quantification of m^6^A methylation are closely related to the reversible enzymatic reaction system behind. Among them, METTL3 is able to independently methylate substrate RNA in vitro, and *METTL3* knockdown leads to a significant reduction of transcriptome m^6^A methylation level, together highlighting METTL3 as the core component of m^6^A writer complex [4]. On the other hand, the m^6^A modification can be removed by eraser proteins such as FTO and ALKBH5 [5]. Through sophisticated procedures executed by the m^6^A writers and erasers, a complicated epitranscriptome is formed. To date, near 500,000 human single‐nucleotide m^6^A sites has been identified [6]. Comprehensive m^6^A profiling by methylated RNA immunoprecipitation sequencing (MeRIP‐seq) has revealed distinct but partly shared m^6^A profiles across different cell types [7, 8]. Nonetheless, considering the different transcriptome context among different cell types, direct and systematic measure of the regulatory effects of m^6^A is necessary. Unfortunately, there remains a lack of sizable high‐throughput profiling of m^6^A's effects, and consequently, to what extent the m^6^A regulatory effects are shared or distinct between different cell types is not clear.

The m^6^A modification is also recognized as the top RNA modification with the greatest number of known recognizing partners, i.e. m^6^A readers. This comprehensive and sophisticated reader toolkit gives rise to the diversity and complexity of the m^6^A regulatory effects, i.e. m^6^A readouts. Although with controversial about functional specificity [9, 10], it is recognized that several key m^6^A readers play vital roles in gene expression regulations. For example, YTHDF1 would promote the translation of mRNAs with m^6^A modification, while YTHDF2 would reduce the half‐life of m^6^A target transcripts [11, 12]. Nuclear YTHDC1 could recruit pre‐mRNA splicing factor SRSF3 and inhibit the binding of SRSF10 to mRNAs to influence exon inclusion events in alternative splicing [13], IGF2BP family proteins constitute a new clade of m^6^A readers that inhibit mRNA degradation and enhance translation efficiency [14]. Until recent, novel m^6^A readers have been unveiled. For example, ELAVL1 (HuR) preferentially binds elements in the 3'‐UTR that are enriched for AU or U, thereby regulating mRNA stability [15]. TARDBP (TDP43) is identified as a m^6^A‐binding protein that regulates neurodegeneration [16]. By leveraging mass spectrometry, dozens of proteins have shown favorable presence in the m^6^A‐associated proteome, suggesting existence of unknown m^6^A readers, although the direct m^6^A recognition ability of these putative m^6^A readers need further validations [17].

As the key epitranscriptomic factor, m^6^A also exert substantial impacts on key properties of pluripotent stem cells. In fact, early demonstrations of m^6^A regulatory function on embryonic stem cells (ESCs) constitute the pioneer cases supporting the functional importance of m^6^A modification. In 2014, Wang et al. reported that *Mettl3* inhibition decreases the expression of pluripotency‐associated genes (e.g., *Nanog* and *Sox2*) but up‐regulates the expression of developmental markers (e.g., *Sox17*), and ultimately promotes mouse ESCs (mESCs) differentiation [18]. In induced pluripotent stem cells (iPSCs), Chen et al. also showed that increased m^6^A formation promotes cell reprogramming to pluripotency [19]. However, there are also inconsistent results, for example, in 2014, Batista et al. found that depletion of mouse and human *Mettl3* promoted self‐renewal of mESCs and human ESCs (hESCs) by prolonging *Nanog* expression upon differentiation. Subsequently, Geula et al. showed that deletion of m^6^A could lead to either defected or accelerated differentiation depending on the pluripotent state of the mESCs, i.e. naive or primed [20]. These results imply complexity in the functional readout of the m^6^A tags in ESCs. Besides, various m^6^A readers also play essential roles in regulating ESC pluripotency. Knockout of *YTHDF* genes inhibits mESCs exit from pluripotency as evidenced by poor differentiation during teratoma and embryoid body formation [21]. Deletion of YTHDC1 initiates cellular reprogramming to a 2‐cell like state [22]. These studies emphasize the importance of the m^6^A modification in regulating the self‐renewal and differentiation process of ESCs. However, which and how m^6^A readers exert the specific regulatory effects of hESC differentiation has not been sufficiently investigated.

Together, m^6^A has emerged as the key gene expression regulatory machinery but its regulatory effects and mechanisms have not been fully elucidated. Whether m^6^A showed similar or distinct readouts across different cell types? Are there new readers behind the regulatory effects of m^6^A. And if so, how could these m^6^A readers influence basic stem cell functions including self‐renewal and differentiations? To this end, we comprehensively profiled the three main layers of m^6^A readouts (mRNA half‐life, translation efficiency, and alternative splicing) across five common cell types of different origin (including cancerous A549 and JURKAT, and non‐cancerous hESC, HEK293T, and human umbilical vein endothelial cells (HUVECs)). Besides, quantitative profiling of m^6^A methylation by GLORI, if applicable, was also performed. To exploit these resources, machine learning modeling of the m^6^A readouts was conducted, where putative m^6^A readers were suggested based on the interpretation of model features. Finally, we validated the m^6^A binding ability and regulatory effects of a few of these putative m^6^A readers and unveiled their specific impacts on hESC differentiation.

## 2 Results

### 2.1 Systematic Profiling of m^6^A Methylation Readouts Across Different Cell Types

**FIGURE 1:**
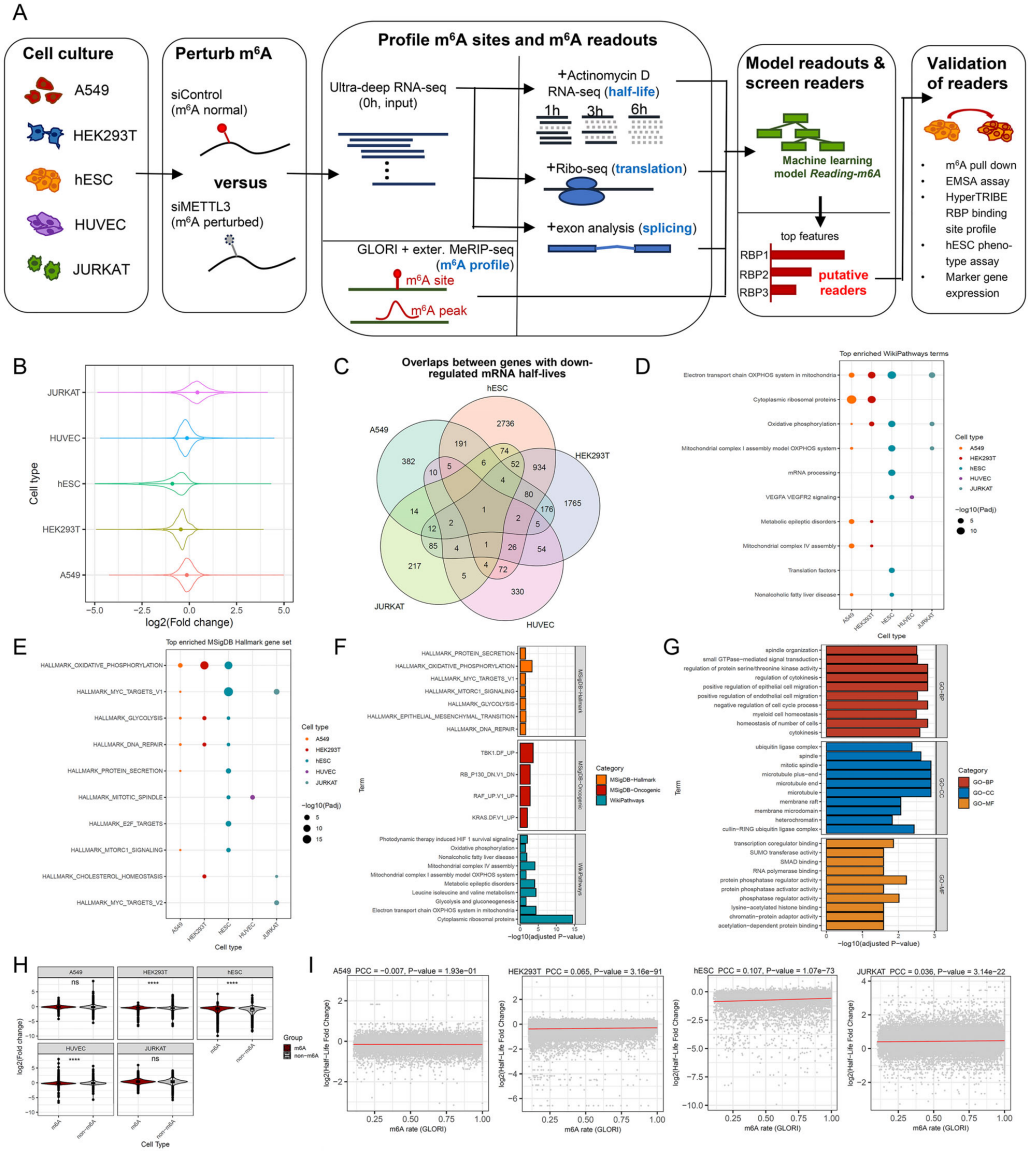
Overview of the experimental design for m^6^A readout profiling and the half‐life readout profiling results. (A) Overview of the experimental design for m^6^A readout profiling. MeRIP‐seq, methylated RNA immunoprecipitation sequencing; Ribo‐seq, ribosome profiling sequencing GLORI, glyoxal and nitrite‐mediated deamination of unmethylated adenosines; RBP, RNA binding protein; EMSA, electrophoretic mobility shift assay; TRIBE, targets of RNA‐binding proteins identified by editing. (B) Overview of m^6^A‐mediated half‐life changes. The comparison was performed between m^6^A‐normal cells (shControl) versus m^6^A‐disrupted cells (shMETTL3) as the background. (C) Venn diagram of half‐life down‐regulated genes in different cell types. (D) Top 10 enriched pathways (WikiPathways) of half‐life down‐regulated genes among different cell types sorted by total ‐log10(adjusted p‐value). (E) Top 10 enriched MSigDB Hallmark gene sets of half‐life down‐regulated genes among different cell types. (F) Top enriched pathways and gene sets for half‐life down‐regulated genes in A549 cells. (G) Top enriched Gene Ontology (GO) functional terms for half‐life down‐regulated genes in HUVEC cells. (H) Violin plots comparing the log2(fold change) of mRNA half‐life in m^6^A‐postive and m^6^A‐negative groups, as per the m^6^A methylation peaks identified by MeRIP‐seq on the corresponding cell types. (I) Correlation between fold change in half‐life and m^6^A methylation levels assessed by GLORI. Data in (D‐G) and (I) were statistically analyzed using Fisher exact test and correlation test, respectively. Data in (H) were statistically analyzed using two‐tailed unpaired Student's *t*‐test. **** means *p* < 0.0001, ns means not significant. For all box plots, boxes cover Q1 to Q3, while whiskers extend to 1.5 IQR.

The overall design of this study is illustrated in Figure 1A. Briefly, we perturbed m^6^A in five representative cell lines through knockdown of core m^6^A writer *METLL3*. The efficiency of the knockdown was verified by RT‐qPCR and Western blot (Figures S1 and S2). By procedure, we could measure the mRNA half‐life, translation efficiency, and alternative splicing readouts of m^6^A by comparing the post‐transcriptional gene expression dynamics of m^6^A‐normal cells (i.e. shControl cells) versus m^6^A‐disrpted cells (i.e. shMETTL3 cells). Actinomycin D‐treated temporal RNA‐seq, Ribo‐seq, and ultra‐deep RNA‐seq were adopted to measure the three main categories of m^6^A readouts, respectively. We introduced a cost‐effective experiment design by performing ultra‐deep RNA‐seq on the untreated (0 h) cells, which could also serve as the start point for temporal RNA‐seq as well as the input sample for Ribo‐seq, with the discrepancy in sequencing library size controlled by ERCC spike‐ins (see Methods). To evaluate whether the difference of sequencing depth between 0 h cells and cells collected from other cell time points, we prepared a balanced RNA‐seq library where the RNA‐seq library of 0 h cells were down‐sampled to the sequence depth of the other time points. A good accordance between the mRNA half‐life readouts estimated from the balanced library and those estimated from the original library was observed (Figure S3A–E), indicating that ERCC spike‐in could effectively control the discrepancy between 0 h and other time points.

The mRNA half‐life was first measured by fitting the gene expression to the mRNA decay curve along the time points. For all cell types, a decrease of absolute mRNA quantification along the time points were observed, demonstrating substantial mRNA degradation with the transcription process inhibited by Actinomycin D. Nonetheless, the decay rate of the mRNA half‐life could be diverged between different m^6^A states, with tendencies toward overall down‐regulation of mRNA half‐lives in m^6^A normal cells (shControl cells) for A549, HEK294T, hESC and HUVEC cells that are similar to previous literature [11, 23], but a tendency toward up‐regulation for JURKAT cells (890, 3203, 4188, 521, 481 down‐regulated genes and 245, 64, 30, 417, 3575 up‐regulated genes for A549, HEK294T, hESC, HUVEC and JURKAT cells, respectively; Figure 1B). Nonetheless, the overlap of half‐life down‐regulated genes is limited between different cell types (Figure 1C), even when limiting the scope within the genes that are widely expressed in all of the five cell types (Figure S3F). Similarly, the overlap of half‐life up‐regulated genes is also limited (Figure S4A,B), together indicating a high cell‐type specificity of the half‐life readout of m^6^A.

The limited overlap between half‐life de‐regulated genes may be simply caused by the threshold in measuring the readout. Thus, we performed enrichment for different functional gene sets and pathways to check if different cell types could show consistent changes at the pathway level. Indeed, for down‐regulated genes, gene sets representing fundamental biological functions such as mitochondrial oxidative phosphorylation, glycolysis, and DNA repairs were shared by multiple cell types, indicating a consensus regulation by m^6^A in these basic cellular functions (Figure 1D,E; Figure S3G). On the other hand, some pathways were only enriched in particular cell type, and these pathways are likely to be associated the functions of specific cell type. For example, multiple oncogenic pathways were enriched in lung cancer cell line A549, whereas cytoskeletal and endothelial cell migration functions were enriched in blood vessel‐derived HUVECs (Figure 1F,G). Similarly, TNA‐α signaling turned out to be the shared pathway between half‐life up‐regulated genes from different cell types (Figure S4C). For up‐regulated genes in JURKAT, they are associated with functions vacuolar transport and vesicle organization, implying its contribution to cell‐cell communications (Figure S4D,E).

By querying to the public MeRIP‐seq methylation profiles from the corresponding cell type, we found both m^6^A‐postive and m^6^A‐negative genes could show mRNA half‐life changes (Figure 1H). Therefore, to be specific, unless otherwise stated, all of the subsequent analyses of mRNA half‐life changes were limited within the scope of m^6^A‐postive group. To precisely describe the relationship between m^6^A modification levels and half‐life changes, we evaluated the m^6^A methylation levels of target genes in different cells using glyoxal and nitrite‐mediated deamination of unmethylated adenosines (GLORI) technique [24]. The quantitative m^6^A map of four out of the five cell types were included, where A549, hESC and JURKAT data were newly measured in this study. HUVEC was not included in GLORI analysis because of its proliferation capacity is insufficient to accumulate required amount of input cells. Only a small fraction of false positive m^6^A sites that could be also identified in the *METTL3* knockdown cells, and these sites were excluded in the subsequent analysis (Figure S5A). The m^6^A methylation sites identified by GLORI were all enriched near the stop codon (Figure S5B), which is a typical topology feature of m^6^A sites on coding genes [2]. Our GLORI experiments could capture m^6^A sites with medium‐to‐high modification ratios, although low‐rate sites were relatively underrepresented because of the reduced sequencing depth due to the cost concerns (Figure S5C). Besides, the m^6^A sites and genes were partially shared between different cell types, and noticeable overlaps between m^6^A target genes identified by GLORI and those from MeRIP‐seq could be observed, confirming that the main m^6^A target genes were captured (Figure S5D–F). Finally, both m^6^A‐postive and m^6^A‐negative genes could show mRNA half‐life changes (Figure S5G), therefore we focused on a direct correlation analysis between the absolute quantification of m^6^A sites and the mRNA half‐life fold‐change of their target genes within the m^6^A‐positive group. Weak correlations between m^6^A modification rates and the half‐life changes can be observed in some cell types, suggesting the half‐life regulation would partly rely on the methylation level of the target genes (Figure 1I). Nonetheless, the correlations were weak, and even became near negligible when considering MeRIP‐seq peak score as the measurement of m^6^A levels (Figure S3H), suggesting that other factors, such as the RNA binding protein (RBP) context of m^6^A sites, may also have significant contributions. Indeed, noticeable variability of expression levels of known m^6^A readers could be observed (Figure S6A), For a more direct comparison, the variability in m^6^A reader regulation was estimated via the coefficient of variation of m^6^A‐to‐reader binding site distances on the transcripts across five cell types, while the variability in m^6^A readouts was measured by the standard deviation of log2(fold change). The results suggested that genes showing high variability in half‐life readout (Figure S6B) tend to have less variable m^6^A reader regulation, implying a closer relationship of genes showing higher half‐life readout variability with m^6^A reader regulations. We will investigate this direction further with machine learning modeling technique in the corresponding section below. On the other hand, however, when analysis the m^6^A‐negative genes showing mRNA half‐life changes, few known m^6^A readers could be observed among the top associated RBPs (Figure S6C), indicating the m^6^A independent effect of METTL3 are likely to be mediated by another set of regulators other than m^6^A readers, similar to the observation documented in the previous literature [25, 26].

**FIGURE 2:**
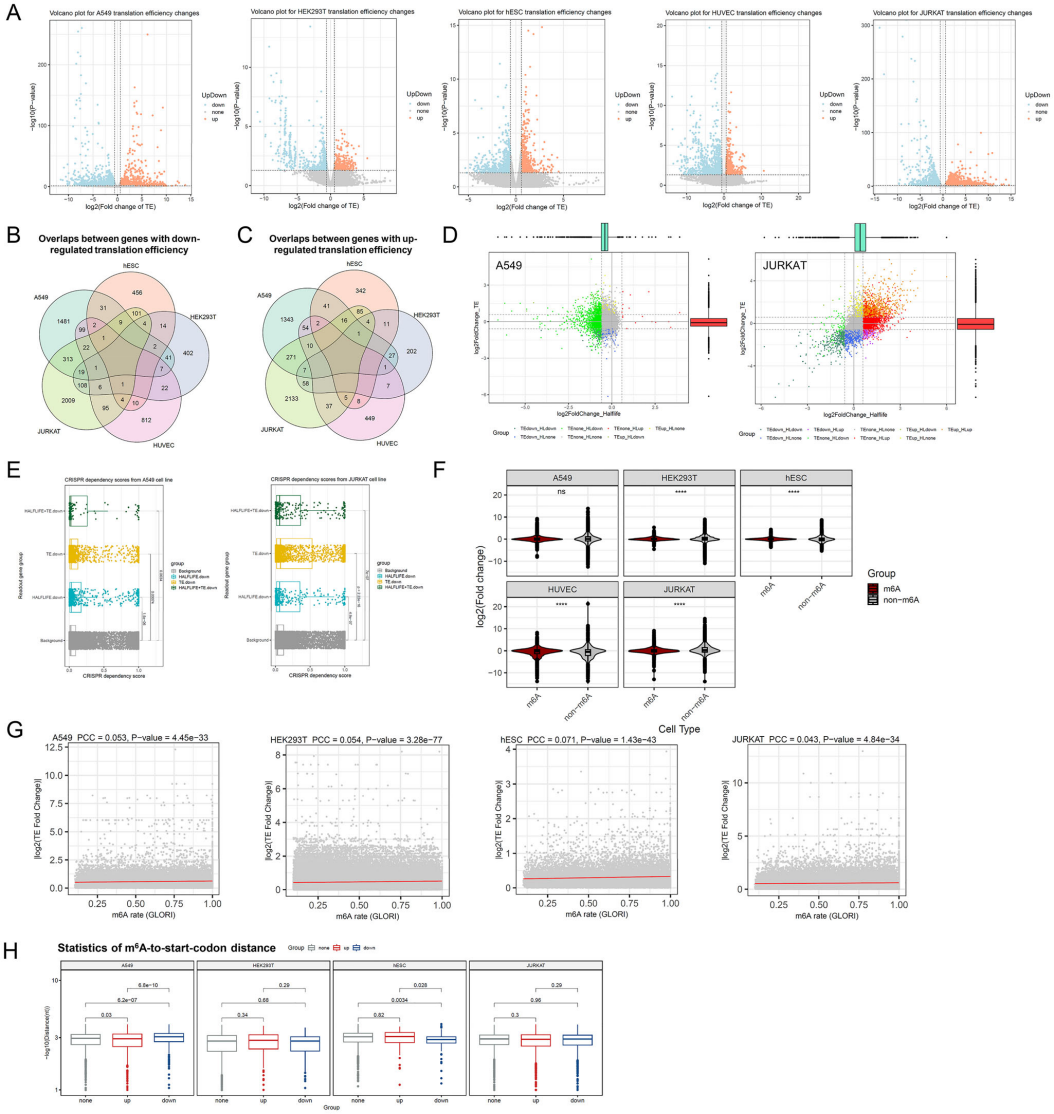
m^6^A methylation regulates translational efficiency in different cell types. (A) Volcano plot of m^6^A‐mediated changes in translational efficiency. The comparison was performed between m^6^A‐normal cells (shControl) versus m^6^A‐disrupted cells (shMETTL3) as the background. (B‐C) Venn diagram of translational efficiency down‐regulated (B) and up‐regulated (C) genes in different cell types. (D) Scatter plot showing translational efficiency versus half‐life changes in A549 and JURKAT cells. (E) CRISPR‐based gene importance scores of genes with half‐life and/or translation efficiency changes in cancer cell lines A549 and JURKAT compared to genome background. (H) Violin plots comparing the log2(fold change) of translation efficiency in m^6^A‐postive and m^6^A‐negative groups, as per the m^6^A methylation peaks identified by MeRIP‐seq on the corresponding cell types. (G) Correlation between fold change in translation efficiency (in absolute values) and m^6^A methylation levels assessed by GLORI. (H) Box plots comparing the minimum m^6^A‐site‐to‐strart‐codon distance between genes showing different translational efficiency changes. Data in (E), (F), and (H) were statistically analyzed using two‐tailed unpaired Student's *t*‐test. **** means *p* < 0.0001, ns means not significant. For all box plots, boxes cover Q1 to Q3, while whiskers extend to 1.5 IQR. Data in (G) was statistically analyzed using correlation test.

Next, we profiled the translation efficiency readout of m^6^A. In terms of translation efficiency, different cell types showed more balanced numbers of up‐ and down‐regulated genes except HUVEC cells (2028, 627, 635, 1082, 2693 down‐regulated genes and 1773, 318, 515, 573, 2627 up‐regulated genes for A549, HEK294T, hESC, HUVEC, and JURKAT cells, respectively; Figure 2A; Figure S7A). The overlap of genes showing translation efficiency changes was also very limited between different cell types, for both up‐ and down‐regulated genes (Figure 2B,C), even when focused on the widely expressed genes (Figure S7B,C). Some of the genes showed both half‐life and translation efficiency changes (Figure 2D; Figure S8A). In A549 cell lines, genes with half‐life or translation efficiency changes had higher importance (DepMap dependency [27]) scores than background, and those with both types of changes ranked the highest; in JURKAT, higher importance scores of m^6^A‐regulated genes were also observed (Figure 2E). Similar to the observation in half‐life readout analysis, some fundamental pathways are also the favored target of the translation efficiency regulations of m^6^A (Figure S8B–G). We found both m^6^A‐postive and m^6^A‐negative genes could show translation efficiency changes (Figure 2F; Figure S5H). Therefore, to be specific, unless otherwise stated, all of the subsequent analyses of translation efficiency were limited within the scope of m^6^A‐postive group. When focusing on m^6^A‐postive group, we observed that both the GLORI‐estimated and MeRIP‐seq‐estimated methylation levels only show weak or negligible correlation with the fold change in translation efficiency (Figure 2G; Figure S7D). We also noted that genes with up‐ or down‐regulated translation efficiency did not always tend to have a m^6^A site near the start codon (Figure 2H). These results indicate that neither m^6^A level nor m^6^A topology is sufficient to fully explain the translation efficiency readout of m^6^A. Instead, similar to the observation for half‐life changes, genes showing high variability in translation efficiency readout (Figure S6D) tend to have less variable m^6^A reader regulation, implying a closer relationship of genes showing higher translation efficiency readout variability with m^6^A reader regulations. On the other hand, also similar to the observation for half‐life changes, when analyzing the m^6^A‐negative genes showing translation efficiency changes, few known m^6^A readers could be observed among the top associated RBPs (Figure S6E).

**FIGURE 3:**
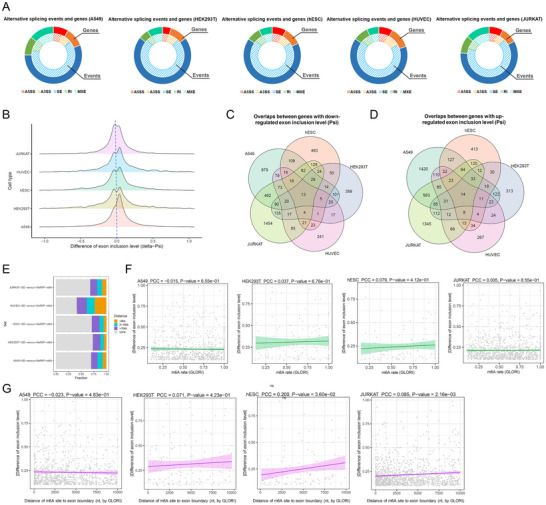
m^6^A methylation regulates alternative splicing in different cell types. s (A) Circular plot summarizing different types of alternative splicing changes mediated by m^6^A. The inner and outer loops representing the percentages of alternative splicing events and genes, respectively. (B) Ridge plot summarizing the m^6^A‐mediated exon inclusion ratio (Psi) changes. The comparison was performed between m^6^A‐normal cells (shControl) versus m^6^A‐disrupted cells (shMETTL3) as the background. (C‐D) Venn diagram of exon inclusion ratio down‐regulated (C) and up‐regulated (D) genes in different cell types. (E) Percentage of exons showing exon inclusion ratio changes with m^6^A methylation peaks (identified by MeRIP‐seq assay on the corresponding cell types) near their exon boundaries. (F) Correlation between changes in exon inclusion ratio (in absolute values) and m^6^A (within 10 kb to exon boundary) methylation levels assessed by GLORI. (G) Correlation between changes in exon inclusion ratio (in absolute values) and the distances from m^6^A methylation sites to exon boundaries. Data in (F‐G) were statistically analyzed using correlation test.

The ultra‐deep sequencing enabled extensive detection of alternative splicing events, where the m^6^A‐mediated alternative splicing was mainly manifested as exon skipping, at both the event and the gene level (Figure 3A). Therefore, subsequent analyses of alternative splicing were focused on exon skipping. Generally, an increase of exon inclusion ratio (Psi) could be observed in m^6^A‐normal cells (2944, 1117, 1182, 750, 3753 Psi down‐regulated exons from 2108, 929, 999, 651, 2654 genes and 4080, 1026, 1201, 895, 3737 up‐regulated exons from 2811, 852, 986, 757, 2636 genes for A549, HEK294T, hESC, HUVEC and JURKAT cells, respectively; Figure 3B), consistent with previous studies [13, 28]. Genes showed altered exon inclusion were less shared between different cell types (Figure 3C,D). Basic functions like organelle organization and DNA repair are enriched for these genes (Figure S9). However, it is noteworthy that although m^6^A identified from GLORI were distributed in both introns and exons (Figure S10A), which is similar to a previous study [29], only a small proportion of these exons had known m^6^A methylation sites on themselves (Figure S10B) or in their vicinity (Figure S5I). Similar results can be observed when using the m^6^A identified from MeRIP‐seq (Figure S10C; Figure 3E), together suggesting that there might be a greater influence of indirect factors on the observed exon inclusion changes. Therefore, to reduce the influence of indirect factors, unless otherwise stated, for the subsequent analysis we only considered exons harboring m^6^A methylation sites within 10 kb flanking the exon boundary. Among these exons, the m^6^A‐to‐exon‐boundary distances show weak or no correlation with the changes in exon inclusion ratios (Figure 3F). In addition, GLORI‐estimated methylation levels show weak or no correlation with the changes in exon inclusion ratios (Figure 3G). Similar results could be observed when using MeRIP‐seq data (Figure S10D,E), together suggesting that simple transcript topological features are not sufficient to predict the alternative splicing readout of m^6^A. Besides, when analyzing the m^6^A‐negative genes showing exon inclusion ratio changes, few known m^6^A readers could be observed among the top associated RBPs (Figure S10F).

### 2.2 Predicting m^6^A Readouts Based on the RBP Binding Context

**FIGURE 4:**
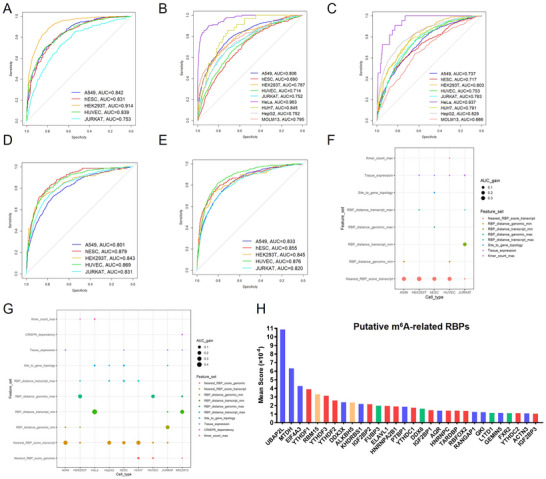
Machine learning models of m^6^A readouts and their top predictive features. (A‐E) ROC curve assessing the performances of machine learning models (termed as Reading‐m^6^A) for predicting the m^6^A readout effects in terms of half‐life down‐regulated genes (A), translational efficiency down‐regulated genes (B), translational efficiency up‐regulated genes (C), exon inclusion ratio down‐regulated exons (D), and exon inclusion ratio up‐regulated exons (E) in different cell types. (F) Bubble plot summarizing the performance contributions (as evaluated by the gain of AUC) of different feature sets for predicting half‐life down‐regulated genes. (G) Bubble plot summarizing the performance contributions of different feature sets for predicting translational efficiency down‐regulated genes. (H) Bar plot summarizing the RNA binding proteins (RBPs) with high feature importance score in the machine learning models, where red color indicates known m^6^A readers, orange color indicates known m^6^A writers/erasers, green color indicates the newly identified m^6^A reader in this study (FUBP3, DDX6, L1TD1 and FXR2), and blue color indicates the RBPs showing no significant m^6^A binding ability in our RNA pull‐down assay.

Given that m^6^A methylation level and topology could not accurately predict m^6^A functional readout, we paid more attention on the genomic context, especially the RBP binding context of the m^6^A sites. To this end, we systematically collected and re‐analyzed high‐throughput RBP binding site data from public databases (see Methods) to comprehensively evaluate the associations between m^6^A sites and RBP binding sites. To ensure applicability, we used MeRIP‐seq‐based m^6^A site annotation here (see Methods). The score of the closest RBP binding site and the genomic/transcript distance were calculated as the RBP features of an m^6^A site. We also introduced the distribution features of m^6^A sites in transcripts, tissue expression level, gene importance score and k‐mer features to better characterize m^6^A sites and their target genes. The above features were inputted into the XGBoost model to build the m^6^A readout predictor, hereafter referred as Reading‐m6A. Reading‐m6A could deal with five readout categories: half‐life down‐regulation, translation efficiency down‐regulation and up‐regulation, and exon inclusion ratio decrease or increase. The half‐life up‐regulation cases were not considered due to the limited number of this type of genes. Because of the obvious differences in m^6^A readout between different cells, each cell type was modeled separately and the prediction accuracy of the model was assessed using tenfold cross‐validation. Only genes harboring m^6^A modification and exons with approximal m^6^A sites were considered. Genes without required encoding features were also discarded (see Methods). The results showed that the overall prediction performance of the model was acceptable, with an average AUROC of 0.8, especially for half‐life and alternative splicing readouts (correctly predicting 366/526, 1414/1624, 1969/2853, 305/426, 139/254 half‐life down‐regulated genes, 305/500, 108/144, 113/142, 231/297, 496/697 exon inclusion rate down‐regulated exons, and 607/873, 90/118, 147/188, 264/321, 570/815 exon inclusion rate up‐regulated exons for A549, HEK293T, hESC, HUVEC and JURKAT at 80% specificity; ROC curve shown in Figure 4A,D,E). To further test this modeling approach, we also introduced translation efficiency readout data derived from public Ribo‐seq datasets. Four cell lines (HeLa, HepG2, Huh7 and MOLM13) were included. and the predictive performance of the model is equally acceptable in these cells except MOLM13 (correctly predicting 614/937, 118/190, 121/272, 396/839, 841/1466, 124/134, 333/566, 100/174, 169/264 translation efficiency down‐regulated genes and 493/959, 493/959, 105/173, 172/330, 269/493, 724/1280, 80/88, 789/1125, 126/203, 126/323 translation efficiency up‐regulated genes at 80% specificity for A549, HEK293T, hESC, HUVEC, JURKAT, HeLa, HepG2, Huh7 and MOLM13 cells; ROC curve shown in Figure 4B,C). Compared to other classical machine learning frameworks, XGBoost showed the best performance for most cases (Figure S11A); and its performance is insensitive to hyperparameter optimization, with at most 0.01 AUC gain after hyperparameter optimization (Figure S11B). We also checked whether considering GLORI‐based m^6^A sites, especially their m^6^A modification rates, could result in better prediction performance. The results demonstrated that replacing MeRIP‐seq‐based m^6^A site annotation with GLORI‐based ones could benefit the performance in some cases but reduce the performance in other cases, even the highly comprehensive GLORI profile from HEK293T was adopted, while including m^6^A modification rate features show marginal influence to the performance (Figure S11C). Therefore, we kept MeRIP‐seq‐based m^6^A site annotation as the primary source to build our models.

While the above models were established on the comparison between METTL3 knockdown and control cells, we also extended the test by including METTL3 inhibitor‐treated cells in the prediction. More specifically, H460 cells were treated with METLL3 inhibitor STM2457 and the changes in mRNA half‐life are profiled accordingly. In H460 cells, METLL3 inhibitor induced sizable amount of both up‐ and down‐regulations of mRNA half‐life (Figure S12A–C), enabling modeling of both half‐life down‐regulation and up‐regulation readouts. Two prediction models were established accordingly based on this mRNA half‐life alteration profiles in METLL3 inhibitor‐treated H460 cells. The models correctly predict 721/870 half‐life down‐regulated genes and 316/392 up‐regulated genes at 80% specificity, with ROC curves also indicating acceptable performance (Figure S12D).

One of the main reasons we used the XGBoost framework for modeling was its good interpretability. We found that the RBP‐based feature sets indeed ranked the best for their contribution to AUC. For half‐life and translation efficiency readout predictions, the binding scores of RBP‐binding sites near the m^6^A site were the most important feature set (Figure 4F,G; Figures S11D and S12E). While the transcript of genomic distance between m^6^A and RBP‐binding site ranked top in exon inclusion readout predictions (Figure S11E,F). Gene topology features and tissue expression contributed to the performance to a much smaller extent. K‐mer features showed only marginal or no contribution to AUC, implying the context features related to m^6^A readouts were largely covered by current RBP binding site data. On the other hand, we also noted the performance for cross‐cell‐type prediction are noticeably reduced due to the high cell specificity of m^6^A readouts (Figure S11G–K). Therefore, instead of establishing a generic model suitable for all cell types, in the subsequent investigation, we focused on the interpretation and experimental validation of the important RBP features. Indeed, further analysis of the importance scores of individual RBP features revealed that some known m^6^A readers had high importance scores, while other important RBP features suggested putative novel m^6^A readers. s for which we will be experimentally validated in the next sections (Figure 4H; Figure S13).

### 2.3 m^6^A Readout‐Related RBPs as the Novel m^6^A‐Binding Proteins or m^6^A Readers

**FIGURE 5:**
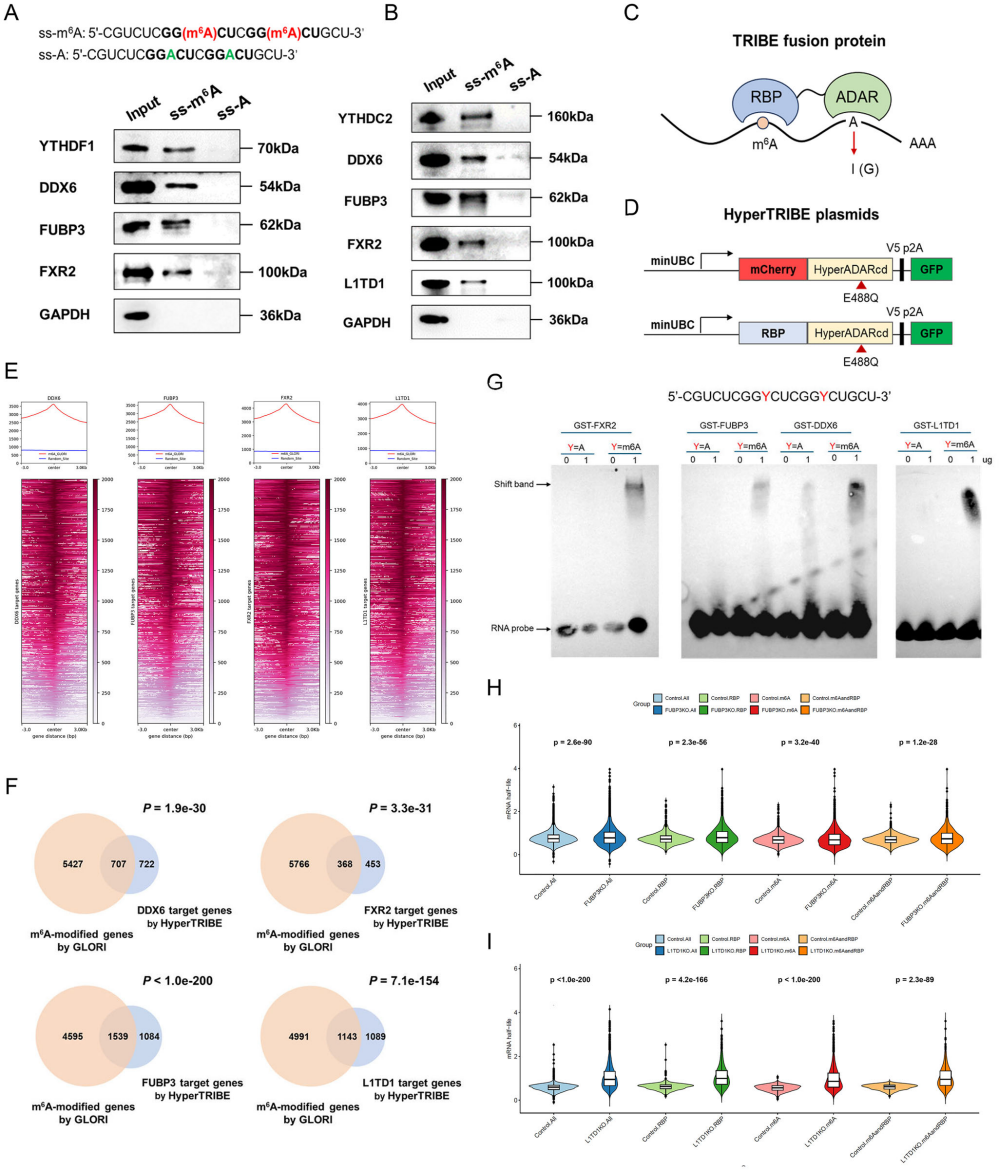
Identification of m^6^A binding proteins and m^6^A readers in hESCs. (A) RNA pull‐down assays that verify the binding of RBPs to m^6^A in A549 cells. (B) RNA pull‐down assays that verify the binding of RBPs to m^6^A in hESCs. (C) Overview of principle of the TRIBE/HyperTRIBE for transcriptome‐wide identification of RBP binding sites. (D) Diagram of plasmid constructs for HyperTRIBE. (E) Heatmap showing the distribution of HyperTRIBE‐identified RBP binding sites near the m^6^A methylation sites determined by GLORI in hESCs. (F) Venn diagram showing the intersection between RBP target genes identified by HyperTRIBE and m^6^A target genes identified by GLORI in hESCs. (G) RNA EMSA assays showing the direct binding of DDX6, FUBP3, FXR2, and L1TD1 with m^6^A probe but not the non‐modified RNA probe. (H) Comparison of mRNA half‐life between *FUBP3* knockout (*FUBP3*‐KO) hESCs and wildtype controls within different scopes of gene sets. (I) Comparison of mRNA half‐life between *L1TD1* knockout (*L1TD1*‐KO) hESCs and wildtype controls within different scopes of gene sets. Data in (F) and (H‐I) were statistically analyzed using Fisher exact test and two‐tailed unpaired Student's t‐test, respectively.

We noticed some RBPs with high feature importance in our m^6^A readout models have been reported as m^6^A readers [4]. These RBPs included typical m^6^A readers such as YTHDF1/2/3, YTHDC1/2, and IGF2BPs as well as some recently discovered m^6^A readers like TARDBP and ELAVL1 (Figure 4H; Figure S13). To further screened novel m^6^A‐binding proteins, we used methylated single‐stranded RNA bait (ss‐m^6^A, with the consensus sequence GG‐m^6^A‐CU) or unmethylated control RNA (ss‐A) to perform RNA pull‐down assay, where several known m^6^A readers (YTHDF1, YTHDC2, RBFOX2, TARDBP) were included as the positive controls (Figure 5A,B; Figures S14–S16). The pull‐down screening was mainly performed in A549 cells, and the results showed that DDX6, FUBP3 and FXR2 could strongly bind to the methylated bait instead of the unmethylated control (Figure 5A; Figure S15A), and their binding to m^6^A was also confirmed in hESCs (Figure 5B; Figure S15B). Because L1TD1 has no expression in A549 cells, we could only validate L1TD1 binding to methylated bait in hESCs only (Figure 5B; Figure S15B).

We further investigated the transcriptome‐wide binding preference of these candidate readers toward m^6^A sites by using TRIBE/HyperTRIBE technique [30, 31, 32]. TRIBE involves fusing the target RBP to the catalytic domain of the RNA editing enzyme ADAR. When the fusion proteins are expressed in target cells, the A‐to‐I editing at residues in proximity to RBP binding sites was catalyzed by ADAR, providing a signal detectable in conventional RNA‐seq (Figure 5C). HyperTRIBE exploits a mutated version of ADAR domain to improve its activity thus enhance the A‐to‐I signals [31, 32]. To demonstrate the feasibility of HyperTRIBE approach, we first applied this technique to well‐known m^6^A reader YTHDF1 in HEK293T cells (Figure 5D). After applying stringent thresholds and subtracting negative controls (see Methods), over 100,000 sites targeted by YTHDF1‐ADAR fusion protein were observed. The YTHDF1 target genes identified by HyperTRIBE significantly overlapped with known m^6^A‐modified genes identified by GLORI (*p* = 2.9e‐43, Fisher's exact test; Figure S17A). Similar results were observed when using MeRIP‐seq data [33] (*p* < 1.0e‐200, Fisher's exact test; Figure S17B). Moreover, the majority of YTHDF1 target genes overlapped with those identified by the previous TRIBE assay [9] (Figure S17C). These results together demonstrate the feasibility of HyperTRIBE.

Then we applied HyperTRIBE to DDX6, FUBP3, FXR2 and L1TD1 proteins in hESCs. The enrichment heatmaps and profiles showed that DDX6, FUBP3, FXR2, and L1TD1‐binding sites were all notably enriched near m^6^A sites rather than non‐m^6^A sites (Figure 5E). When comparing DDX6, FUBP3, FXR2 and L1TD1 target genes with the m^6^A target genes (identified either by our GLORI profiling or by MeRIP‐seq), significant overlaps could be observed (Figure 5F; Figure S17D). We also found significant overlaps between the HyperTRIBE‐identified target genes and the target genes identified from CLIP‐like techniques (Figure S17E).

Since RNA pull‐down and HyperTRIBE assays could not exclude indirect m^6^A binding of RBPs, we further performed RNA electrophoretic mobility shift assay (EMSA) experiments to validate the direct binding of DDX6, FUBP3, FXR2 and L1TD1 to m^6^A‐modified RNA. EMSA assay is a fully in‐vitro assay that performed on purified recombinant RBPs and synthesized RNA probes with or without m^6^A modification, excluding other cellular RNAs and proteins in the reaction system. More specifically, EMSA was conducted to evaluate the binding specificity of several GST‐fused RNA‐binding proteins (GST‐DDX6, GST‐FXR2, GST‐FUBP3 and GST‐L1TD1) to the synthetic RNA probes. The results revealed consistent and pronounced mobility shifts when each protein was incubated with the m^6^A‐modified RNA probe (ss‐m^6^A), indicating robust binding between these proteins and the modified RNA. In contrast, incubation with the unmodified RNA (ss‐A) resulted in markedly reduced or absent shift signals, demonstrating a clear dependence of the interactions on m^6^A modification. Taken together, the above results demonstrated that DDX6, FUBP3, FXR2 and L1TD1 are novel m^6^A binding proteins in hESCs (Figure 5G).

To further elucidated the regulatory functions of the newly identified m^6^A binding proteins, we compared m^6^A readouts between all m^6^A‐modified genes and those targeted by specific m^6^A readers. The results suggested that FUBP3 and L1TD1 would predominantly function through modulating mRNA stability, as evidenced by a significant decrease in the half‐life of their target mRNAs when m^6^A modification existing (Figure S17F,G). We then experimentally verified this possibility by profiling mRNA half‐lives on *FUBP3* knockout (*FUBP3*‐KO) and *L1TD1* knockout (*L1TD1*‐KO) hESCs. In comparison with the control cells, *FUBP3*‐KO and *L1TD1*‐KO cells showed significantly shift toward higher mRNA half‐life at the transcriptome level comparison. This tendency remained the same when focusing on m^6^A target genes, on FUBP3/L1TD1 target genes, or on genes targeted by both m^6^A and FUBP3/L1TD1 (Figures 5H,I). Together, these results suggested FUBP3 and L1TD1 as the m^6^A readers with the regulatory effects on mRNA stability.

### 2.4 Effect of m^6^A‐Binding Proteins and m^6^A Readers on Early Differentiation in hESCs

**FIGURE 6:**
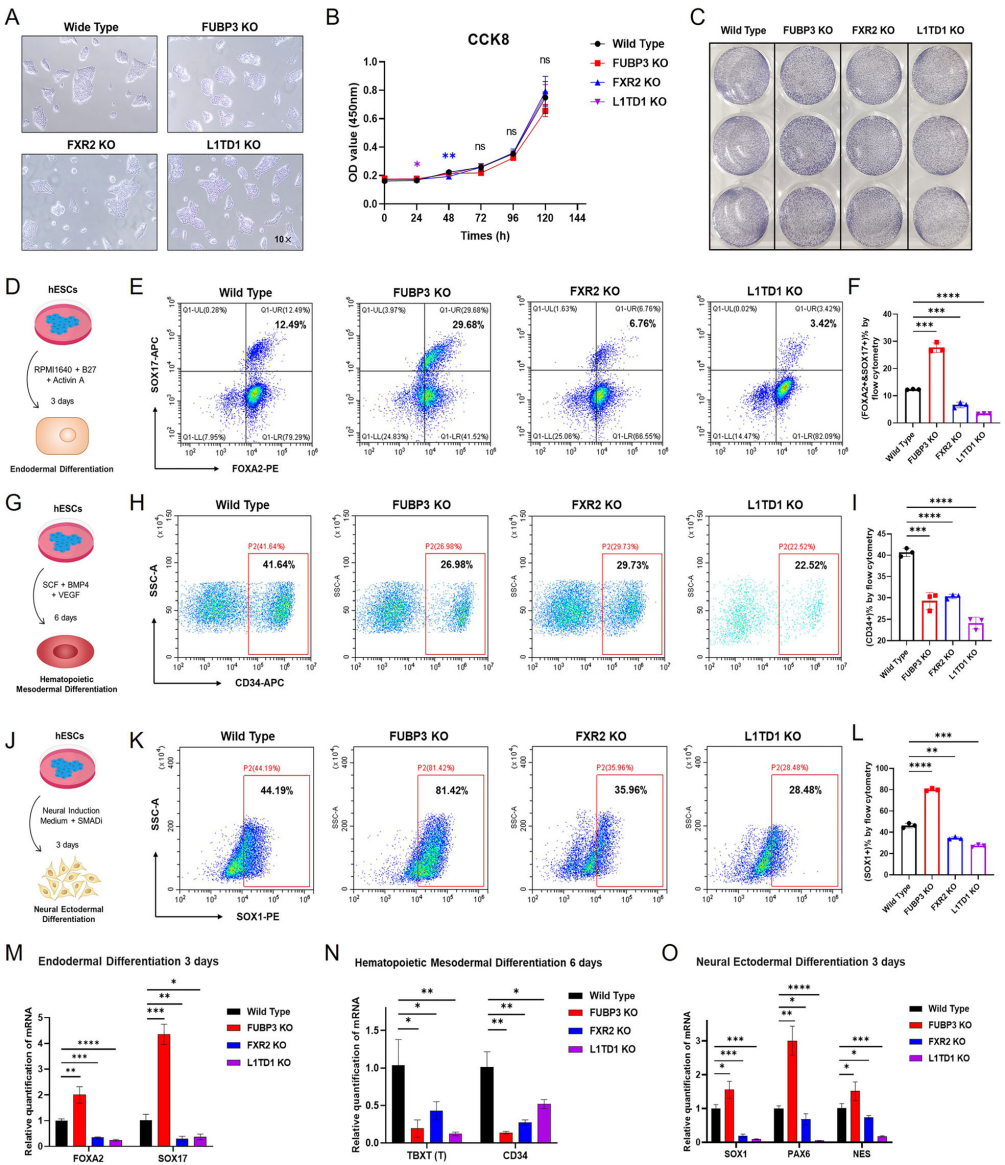
Assessment of the regulatory effects of m^6^A‐binding proteins and m^6^A readers on hESC proliferation and differentiation. (A) Representative images of morphology of WT versus *FUBP3*, *FXR2*, and *L1TD1* KO hESCs under bright field. (B) Growth curve assessing cell proliferation kinetics of *FUBP3*, *FXR2*, and *L1TD1* KO versus WT hESCs. (C) ALP staining assay of WT versus *FUBP3*, *FXR2*, and *L1TD1* KO hESCs. (D) Schematic representation of the induction endodermal differentiation experiment of hESCs. (E‐F) Flow cytometry and statistical histogram analysis of endodermal differentiation of WT versus *FUBP3*, *FXR2* and *L1TD1* KO hESCs. (G) Schematic representation of the induction hematopoietic mesodermal differentiation experiment of hESCs. (H‐I) Flow cytometry and statistical histogram analysis of hematopoietic mesodermal differentiation of WT versus *FUBP3*, *FXR2* and *L1TD1* KO hESCs. (J) Schematic representation of the induction neural ectodermal differentiation experiment of hESCs. (K‐L) Flow cytometry and statistical histogram analysis of neural ectodermal differentiation of WT versus *FUBP3*, *FXR2* and *L1TD1* KO hESCs. (M‐O) RT‐qPCR analysis of definitive endoderm markers SOX17, FOXA2 (M); mesoderm marker TBXT (also known as T) and CD34 (N); and neural‐ectoderm markers SOX1, PAX6, NES in WT versus *FUBP3*, *FXR2* and *L1TD1* KO hESCs (O). Figures in (D), (G), and (J) were drawn with Biovisart (https://biovisart.com.cn) and Bioicons (https://bioicons.com/) with permission. Data in (B), (F), (I), (L), and (M‐O) were statistically analyzed using two‐tailed unpaired Student's *t*‐test. Error bars represent mean ± SD (*n* = 3 independent experiments). For all statistical plots, * means *p* < 0.05, ** means *p* < 0.01, *** means *p* < 0.001, **** means *p* < 0.0001, ns means not significant.

The regulatory function of DDX6 in hESCs has been clarified [34], but the functions of FUBP3, FXR2, and L1TD1 in hESCs have not been fully elucidated. To investigate potential roles for FUBP3, FXR2, and L1TD1 in self‐renewal and early differentiation of hESCs, we constructed *FUBP3*, *FXR2* and *L1TD1* knockout (KO) hESCs by using the CRISPR/Cas9 gene‐editing approach. The validity of KO constructs was confirmed by Western blot and Sanger sequencing (Figures S18 and S19). We first evaluated the effects of these proteins on hESC self‐renewal, but no significant differences in cell morphology between *FUBP3*, *FXR2* and *L1TD1* KO hESCs and wild‐type hESCs were observed (Figure 6A). Besides, compared to wild‐type hESCs, the protein expressions of core pluripotent markers (OCT4, SOX2, and NANOG) did not have significant changes in *FUBP3*, *FXR2*, and *L1TD1* KO cells (Figure S20A,B; Figure S21). RT‐qPCR analysis also found no significantly differences between *FUBP3*, *FXR2* and *L1TD1* KO hESCs and wild‐type hESCs in the expression of core pluripotent markers (Figure S20C). These results revealed that FUBP3, FXR2 and L1TD1 had no significant impact on the expression of core pluripotent genes in hESCs. We also employed the CCK8 and ALP staining assays to investigate the effects of FUBP3, FXR2 and L1TD1 on stem cell proliferation. CCK8 assay showed that the growth rate of *L1TD1* KO and *FXR2* KO cells was changed at 24‐48 h; but when extending to 72‐120 h after cell inoculation, there was no significant difference in proliferation rate between *FUBP3*, *FXR2* and *L1TD1* KO hESCs and wild‐type hESCs (Figure 6B). The ALP staining assay also showed no obvious distinction in the number of colonies formed by *FUBP3*, *FXR2* and *L1TD1* KO hESCs in comparison with wild‐type hESCs (Figure 6C; Figure S20D), indicating that deletion of *FUBP3*, *FXR2* and *L1TD1* had no significant effect on the proliferation rate of hESCs. Together, these results indicated that m^6^A readers FUBP3 and L1TD1, together with m^6^A binding protein FXR2 had no prominent impact on self‐renewal of hESCs.

Next, we investigated the effect of FUBP3, FXR2, and L1TD1 on early differentiation of hESCs by inducing cells into three different germ layers. For endoderm differentiation, Activin A treatment was used in this study. The cells were cultured in RPMI 1640 basic medium supplemented with B27 (Figure 6D). After treating with Activin A for 3 days, cells were collected to detect the expression of endodermal markers FOXA2 and SOX17 by flow cytometry and RT‐qPCR. The flow cytometry results showed that FOXA2^+^SOX17^+^double positive cells were significantly increased in the *FUBP3* KO cultures compared with wild‐type hESCs after endodermal induction for 3 days (Figure 6E,F). However, in FXR2 and L1TD1 KO cultures, the proportion of FOXA2^+^SOX17^+^double positive cells was significantly reduced, indicating that loss of *FUBP3* enhances the ability of hESCs to endodermal differentiation, while loss of *FXR2* and *L1TD1* inhibits the ability to differentiate into endoderm (Figure 6E,F). As for hematopoietic mesodermal differentiation, cells were seeded on low attachment plates to form EB spheres, and then cultured in STEMdiff APEL2 medium supplemented with factors SCF, BMP4 and VEGF for 6 days (Figure 6G). Flow cytometry results showed that *FUBP3*, *FXR2* and *L1TD1* KO hESCs significantly reduced the proportion of mesodermal marker CD34^+^, indicating that FUBP3, FXR2 and L1TD1 were essential for hematopoietic mesodermal differentiation of hESCs. Deletion of *FUBP3*, *FXR2* and *L1TD1* significantly inhibits the ability to differentiate into mesoderm (Figure 6H,I). As for neural ectodermal differentiation, STEMdiff neural induction medium with SMADi were used to induce cells to differentiate into neural progenitor cells by monolayer culture protocol (Figure 6J). Consistent with the tendency of endoderm differentiation, after neural induction for 3 days, more SOX1^+^positive cells were present in the *FUBP3* KO cultures compared to wild‐type group (Figure 6K,L). In *FXR2* and *L1TD1* KO cultures, the proportion of SOX1^+^positive cells was significantly declined, indicating that loss of *FUBP3* promotes the ability of hESCs to neural ectodermal differentiation, while loss of *FXR2* and *L1TD1* inhibits the ability to ectodermal differentiation (Figure 6K,L).

Furthermore, we detected the mRNA expression level of marker genes of three germ layer through RT‐qPCR. Consistent with the flow cytometry results, in endoderm differentiation stage, the mRNA expression levels of FOXA2 and SOX17 were significantly increased in *FUBP3* KO cells but decreased in *FXR2* and *L1TD1* KO cells (Figure 6M). During the hematopoietic mesoderm differentiation stage, the mRNA expression levels of TBXT (also known as T) and CD34 were all significantly reduced in *FUBP3*, *FXR2* and *L1TD1* KO cells compared to wild‐type group (Figure 6N). As for neural ectoderm differentiation stage, the mRNA expression levels of neural‐ectoderm markers SOX1, PAX6 and NES (also known as NESTIN) were significantly increased in *FUBP3* KO cells but decreased in *FXR2* and *L1TD1* KO cells (Figure 6O). These results indicated that FUBP3 may inhibit the endoderm and ectoderm differentiation, but is important for the mesoderm differentiation. FXR2 and L1TD1 genes are crucial for the differentiation of all three germ layers, and absence may significantly affect the differentiation ability of hESCs.

We also noted the distinct binding preference of FUBP3, FXR2 and L1TD1 to genes known to regulate germ layer differentiation between (Figure S22A). FUBP3 exhibited preferential binding to ectoderm regulators NOLC1 [35], CORO1C [36, 37] and mesoderm regulators MAPK1 [38]. FXR2 targeted pan‐lineage differentiation drivers including endoderm FOXD1 [39], ectoderm SOX21 [40], mesoderm TBXT [41], inhibiting three germ‐layer differentiation by regulating target RNA stability. L1TD1 targeted stemness regulators POU5F1 (also known as OCT4) [42] and NANOG [43], exerting impacts on pluripotency exit to inhibit three germ‐layer differentiation. When focusing on FUBP3 that shows different phenotype from the other two proteins, we noted that eight of these FUBP3‐targeted genes that showed pronounced mRNA destabilization with m^6^A modification have been reported to be related to the stem cell differentiation processes (Figure S22B). Indeed, Western blot analysis also indicated that the protein levels of two ectoderm regulators (NOLC1 and CORO1C) significantly increased after *FUBP3* KO (Figure S22C,D; Figure S23). Half‐life assays by actinomycin‐D‐treated RT‐qPCR suggested that the mRNA stability of *NOLC1* and *CORO1C* is significantly increased in *FUBP3* KO hESCs (Figure S22E,F). Previous studies have demonstrated that NOLC1 can act as a nucleolar protein involved in regulation of neural differentiation and neural crest specification; [35] while CORO1C plays an important role in neural crest cell migration and neural development [36, 37]. Together, the above results revealed that FUBP3 could down‐regulate the mRNA stability and protein expression levels of NOLC1 and CORO1C, and this post‐transcriptional regulation ultimately potentiates the neural differentiation capacity of hESCs and modulates the early differentiation trajectory of hESCs (Figure S22G).

## 3 Discussion

In recent years, m^6^A has emerged as a key RNA modification in various biological processes and diseases [4, 44]. Thanks to significant advances in high‐throughput sequencing technologies such as MeRIP‐seq [1, 2] and GLORI [24], systematic mapping of m^6^A modification sites and their dynamic changes in different tissues, conditions and cell types has become feasible [3, 6, 7, 8]. On the other hand, m^6^A modification exhibits diverse post‐transcriptional functional readouts, including changes in RNA half‐life, in translation efficiency, and in alternative splicing [45]. Despite the diverse functional readouts of m^6^A, there is a lack of systematic measurement and comparison of m^6^A post‐transcriptional functional readouts across multiple cell lines. In fact, an extensive search of the public omics database GEO could only yield a very limited number of cell lines where the m^6^A methylome could be coupled with its readouts. For example, we obtained the m^6^A profiles along with Ribo‐seq data for only four cell lines (HepG2, HeLa, Huh7, and MOLM13) with acceptable quality, and these data were introduced as an expanding dataset for translation efficiency readout models. Even for alternative splicing, which can be measured by RNA‐seq, few datasets could meet the required sequencing depth for comprehensive alternative splicing identification. To this end, in this study we first selected five representative cell lines (A549, hESC, HEK293T, JURKAT and HUVEC) and performed temporal transcriptome sequencing, Ribo‐seq and ultra‐deep RNA sequencing on m^6^A‐normal (siControl) and m^6^A‐disrupted (siMETTL3) cells. Our high‐throughput data provide a comprehensive resource for further exploration of the regulatory mechanism as well as the key regulatory targets of m^6^A methylation. Indeed, machine learning modeling of the profiled m^6^A readouts has successfully prioritized novel m^6^A readers from the RBP features of the models, demonstrating the utility of m^6^A readout profiles.

Noticeable cell type specificity of the m^6^A readouts was observed. On the one hand, the m^6^A methylation rate alone is not sufficient to explain this diversity. There are some overlaps of m^6^A target genes between different cell types. However, the overlaps of m^6^A readouts are limited except for some basic pathways. Furthermore, the correlations between GLORI‐measured m^6^A modification ratios with the post‐transcriptional readouts were compromised, and the inclusion of m^6^A modification ratio did not result in better prediction of m^6^A readouts. Instead, we pay more attention on the association of RBP‐binding context of m^6^A sites and m^6^A readouts. Analysis of our readout prediction models suggests that the prediction performance was largely contributed by RBP‐based features, either the RBP binding site score near the m^6^A site or the distance between RBP binding sites and m^6^A modification sites. Interestingly, we found that there are few RBPs that significantly contribute to the predictions of readouts, including some well‐known m^6^A readers, and screened novel m^6^A binding proteins and m^6^A readers based on this candidate list. Together, our results emphasize the importance of m^6^A readers, which form a complex regulatory context that controls how the m^6^A modification is interpreted in cells. On the other hand, it is likely that cell type specificity is overestimated here, when compared to the entire repository of human cell types. To be honest, we intentionally selected five cell types from the different cell clades that show distinctive gene expression patterns to ensure representativeness of our data at an affordable cost. When focusing on a specific physiological or disease process, our readout analysis pipeline would be applied to similar cell lines to investigate the consensus m^6^A readout targets and mechanisms.

In this study, we screened DDX6, FUBP3, FXR2 and L1TD1 as the novel m^6^A binding proteins and assessed their roles in regulating pluripotency in hESCs. DDX6 is an RNA helicase belonging to the Dead Box family, which participates in many aspects of RNA metabolism including processing body (P‐body) formation [46], mRNA storage and decay [47], microRNA pathways [48], and translational suppression [47]. DDX6 plays an essential role in regulating self‐renewal and differentiation. Di Stefano et al. has reported that DDX6 is required for ESC exit from the pluripotent state by mediating the translational suppression of target mRNAs in P‐bodies [34]. Unlike DDX6, the functions of FUBP3, FXR2 and L1TD1 in ESCs is less explored. FUBP3 is an RNA‐binding protein that can bind to the far upstream element (FUSE) of specific RNA molecules, thereby regulating gene expression. In cancer, FUBP3 functions as an oncogene or tumor suppressor gene depending on the type and context of the cancer [49, 50, 51]. However, the role of FUBP3 in cell fate transitions remains largely unclear. Our data in the first time revealed that FUBP3 exerted important impact on early differentiation of three germ lays in hESCs. FXR2 is also an RNA‐binding protein related to Fragile X syndrome. FXR2 are highly enriched in mammalian brains, and has important effects on neurogenesis by regulating the proliferation and differentiation of neural progenitors in mice [52]. In this study, we observed that FXR2 deletion in hESCs impaired early neural development, substantially expanding the scale of FXR2 in neural development regulation. L1TD1 gene was first identified from the expressed sequence tag libraries by Yamanaka group. L1TD1 is abundantly expressed in ESCs and rapidly downregulates upon differentiation [53]. L1TD1 was reported as a part of the pluripotency interactome network of OCT4, SOX2, and NANOG [54]. Interestingly, L1TD1 is dispensable for the maintenance of pluripotency in mESCs, despite its specific expression pattern [53]. We observed that LITD1 deletion had no significant effects on self‐renewal in hESCs. Of note, hESCs with LITD1 showed significant defects in early differentiation of three germ layers, meaning that L1TD1 play an essential role in human early embryonic development. In addition, we revealed the molecular mechanism underlying FUBP3‐mediated mRNA destabilization and identifies two functional targets, NOLC1 and CORO1C, that orchestrate early differentiation processes of hESCs. Our work uncovered the new roles of FUBP3, FXR2 and L1TD1 in stem cell fate control, and sheds light on post‐transcriptional regulation in pluripotency and the importance of RBPs in pluripotent cells.

Our study also has some limitations. First, the scope of cell types included is not sufficient in comparison with the wide spectrum of human cell atlas. This also has limited the cross‐cell‐type prediction performance of the machine learning models, as it is hard to train a generic predictor for all cell types with such high cell specificity of m^6^A readouts among the selected cell types. Besides, the encoding of RBP features depends on the known binding site annotations from public datasets, which are mostly derived via CLIP‐like sequencing technique which is known to have some sensitivity and reproducibility issues. Therefore, the accuracy of the model is also limited by the quality of current RBP binding site annotations. Second, although the m^6^A readouts were measured between m^6^A‐disrupted and m^6^A‐normal cells, and we only consider m^6^A‐positive genes in correlation analysis, machine learning modeling and HyperTRIBE assays, more accurate evaluations would be obtained from the single‐molecule techniques that could directly split transcriptome in m^6^A‐positve transcripts and m^6^A‐negative transcripts. However, at current stage, single‐molecule level co‐profiling of both m^6^A methylation and its different types of readouts (mRNA stability, translation efficiency, and alternative splicing) is technically difficult, even prohibited [55]. We hope a more accurate, single‐molecule level analysis of m^6^A readout could be achieved in the future with the development of single‐molecule sequencing technique [56]. Thirdly, recent studies have revealed new layers of m^6^A readouts such as the regulatory interplay with chromatin [57], while the m6A‐independent effect of METTL3 perturbation were observed in both previous reports [25, 26] and this study, but this aspect was not intensively investigated in current study. Finally, while we demonstrated the direct m^6^A binding ability and regulatory function on mRNA stability of FUBP3 and L1TD1, our data have not highlighted a clear direction of the post‐transcriptional regulatory role for DDX6 and FXR2, which would be further investigated with the increased amount of data in the future. Despite the above limitations, our study has provided useful resource to bridge the gap between m^6^A methylome landscape and its readouts, and prioritizing interesting novel m^6^A readers for stem cell fate control. We expected the future research could expand the range of cell types studied, improve data resources, and delve deeper into the molecular mechanisms underlying m^6^A functional readouts.

## 4 Methods

### 4.1 Cell Culture

The HEK293T cell line (ATCC, catalog#CRL‐3216, RRID: CVCL_0063), A549 cell line (ATCC, catalog#CCL‐185, RRID: CVCL_0023), JURKAT cell line (ATCC, catalog#TIB‐152, RRID: CVCL_0367) and H460 cell line (ATCC, catalog#HTB‐177, RRID: RRID: CVCL_0459) were obtained from the American Type Culture Collection (Manassas, VA, USA) in three batches (2018, 2023 and 2025). HEK293T and A549 cells were cultured in DMEM medium (Gibco, catalog#C11995500BT) supplemented with 10% fetal bovine serum (FBS, Gemini, catalog#900‐108) and antibiotics. JURKAT cells were maintained in RPMI 1640 medium (Gibco, catalog#C11875500BT) with 10% FBS (Gemini, catalog#900‐108) and antibiotics. The human embryonic stem cell (hESC) line H9 (WA09) was obtained from WiCell Research Institute (USA, catalog#WA09, RRID: CVCL_9773) in 2019 under appropriate material transfer agreements. H9 cells were cultivated in serum‐free, defined human pluripotent stem cell medium (STEEMA, catalog#BB50‐400N) and grown on plates coated with growth‐factor‐reduced Matrigel. hESCs were passaged with non‐enzymatic dissociation by 0.5 mM EDTA (Beyotime, catalog#C0196‐100 mL). Primary human umbilical vein endothelial cells (HUVECs) were isolated from the umbilical vein of newborn infants as previously described [58]. The clean vein of the umbilical cord was first lavaged with 0.25% trypsin at 37°C for 15 min. HUVECs were collected and suspended in Endothelial Cell Medium (ECM, ScienCell, catalog#1001). Isolated HUVECs were cultured between passage 4–6 in ECM for experiment. Cells were grown at 37°C with 5% CO2. H460 cells were maintained in RPMI 1640 medium (Gibco, catalog#C11875500BT) with 10% FBS (Gemini, catalog#900‐108) and antibiotics. All cell types tested negative for mycoplasma contamination, according to the results of the mycoplasma detection kit (Solarbio, catalog#CA1080).

### 4.2 METTL3 Knockdown, Transfection, and Flow Cytometry

The doxycycline‐controlled Tet‐Off/On gene expression systems were used for regulating the activity of METTL3 in cell lines. The sequence targeting *METTL3* were synthesized and inserted into the lentivirus vector GV307 (TetIIP‐TurboRFP‐MCS‐Ubi‐TetR‐IRES‐Puromycin) and the lentiviruses were purchased from GeneChem (Shanghai, China). Then according to the characteristics of different cell lines, lentiviruses transfection was performed using the HiTransG A/P developed by GeneChem. Cells transfected with shRNA lentivirus or negative control lentivirus were cultured for 48 h. The target sequence of *METTL3* was CAAGGAACAATCCATTGTT and the negative control sequence of insertion was TCTCGCTTGGGCGAGAGTAAG. Other information of the oligo synthesis was included in Table S1. After the doxycycline induction for 48 h, the transfected cells expressed red fluorescence (RFP) for roughly estimating the overall transfection efficiency. Cell samples were resuspended in MACS buffer for fluorescence‐activated cell sorting (FACS, BD FACSAria III) to obtain high RFP expression transfected cells.

### 4.3 RNA Extraction

Total RNA was isolated from cell samples using TRIzol (Invitrogen, catalog#15596018) following the manufacturer's instruction. Briefly, cells were lysed with TRIzol reagent at room temperature until the cell deposits disappear and chloroform was added. After centrifuge for 15 min at 12,000×g at 4°C, the aqueous phase was transferred to the new EP tube, mixed with isopropanol and stand at room temperature for 10∼15 min. Then centrifuge for 10 min at 12,000×g at 4°C, the supernatant was discarded and the RNA precipitate was washed with 75% ethanol. After centrifuge for 5 min at 7,500×g at 4°C and dry, RNA was dissolved with RNase‐free water and its concentration was measured by Thermo Scientific NanoDrop.

### 4.4 Real‐Time Quantitative PCR (RT‐qPCR) for mRNA Quantification

Total RNA was extracted from transected cells scribed in ‘RNA‐extraction’, then reverse transcribed using PrimeScript RT Master Mix (TaKaRa, catalog#RR036B). All primers used for RT‐qPCR were listed in Table S2. All RT‐qPCRs were run on Bio‐Rad CFX384 real‐time PCR detection system using PowerUp SYBR Green Master Mix (ThermoFisher Scientific, catalog#A25742). GAPDH was used as internal control to normalize the data across different samples.

### 4.5 Western Blot

Cell samples were collected, washed with ice‐cold PBS and fully lysed with RIPA lysis buffer containing protease inhibitors (Beyotime, catalog#P0013C) on ice. After centrifuge for 30 min at 12,000×g at 4°C, supernatant was collected and protein amounts were quantified using the BCA Protein Assay Kit (Beyotime, catalog#P0012S). After boiling for 10 min, equal amounts of protein were loaded and separated by 10% SDS‐PAGE. After electrophoresis, they were transferred to PVDF membranes (Merck‐Millipore, catalog#ISEQ00010). Membranes were blocked with 5% non‐fat milk (Beyotime, catalog#P0216‐1500 g) for 1 h and then incubated at 4°C overnight with the primary antibodies. After incubating 1 h with secondary antibodies at room temperature, images were obtained using the Gel Doc EZ Imager (Bio‐Rad). GAPDH was used as the loading control. All antibodies used for western blot were listed in Table S3.

### 4.6 High‐Throughput Profiling of Half‐Life Readouts of m^6^A

Unless otherwise stated, the m^6^A readouts were measured by comparing shControl (normal m^6^A level) versus shMETTL3 (disrupted m^6^A level) to intuitively depict the regulatory readouts in presence of m^6^A. The sequencing service related to m^6^A readout estimations was provided by Novogene Biotech (Tianjin, China). The experimental and computational pipelines of each readout category is described as follows. a. mRNA half‐life readout. Cells transfected with METTL3 shRNA or negative control shRNA were re‐seeded into 6 well plates. After the doxycycline induction for 48 h, actinomycin D was added to 2 µg/mL at 0, 1, 3, and 6 h before total RNA extraction. Before construction of the library with mRNA sample, ERCC RNA spike‐in control mix (Invitrogen, catalog# 4456740) was added to each sample following the manufacturer's instruction. NEBNext Ultra RNA Library Prep Kit for Illumina (NEB #E7770) and NEBNext Ultra Directional RNA Library Prep Kit for Illumina (NEB #E7760) were used with 400 ng of total RNA for the construction of sequencing libraries. ERCC spike‐in were added to total RNA before library construction, following the protocol recommended by ERCC spike‐in kit manufacturer. RNA libraries were prepared for sequencing using standard Illumina protocols. Two biological replicates were prepared for each time point. For each sample collected at 1, 3, and 6 h, a 15G amount of paired‐end sequencing reads was obtained. For 0 h sample, we required 60G amount of sequences to ensure ultra‐deep RNA sequencing that is necessary for systematic alterative splicing assay. This differences in data size were properly controlled by the aforementioned ERCC spike‐ins. More specifically, quality control, adapter cutting and low‐quality sequence removal were performed by FastQC, fastp and FASTX toolkits. The clean reads were mapped to human genome (hg38) by HISAT2 software (v2.2.1) and the expression values were calculated by featureCounts command of RSubread package with suggested parameter of the software. The absolute quantification of each sample was performed by using ERCC RNA spike‐ins as the reference, following previous work [11, 59]. The mRNA half‐life was also estimated following the method modified from previous work [11]. The degradation rate of RNA *k* was estimated by log_2_(*A_t_*/*A*_0_ · δ) = −*kt*, where *t* is transcription inhibition time (h), *A_t_* and *A*_0_ represent mRNA quantity at time *t* and time 0, δ is a regulating factor in case the RNA:ERCC ratio is prominently deviated from the expectation. *k* values of 3 and 6 h were calculated: time 3 h versus time 0 h, and time 6 h versus time 0 h. A 1.5‐fold change of half‐life threshold was applied for subsequent analysis. For H460 cells, the mRNA half‐lives were compared between METTL3 inhibitor STM2457 treated cells and control cell to estimate the half‐life readout. H460 cells were treated with 5 µM of STM2457 for 72 h before half‐life profiling. In addition, we also profiled the mRNA half‐lives of FUBP3 knockout hESCs and L1TD1 knockout hESCs together with their corresponding control hESCs to investigate the regulatory effects of FUBP3 and L1TD1. The knockout procedure is described in the corresponding sub‐section below.

### 4.7 High‐Throughput Profiling of Translation Efficiency Readouts

The translation efficiency was measured by ribosome profiling sequencing (Ribo‐seq). The reported protocols [60] was adapted with the following modifications: Before cells collection, cycloheximide (CHX, HARVEYBIO, catalog#C21865‐100 mg) was added to the culture media at 100 µg/mL for 5 min. Eighty million cells from each group were collected, rinsed in ice‐cold PBS with 100 µg/mL CHX. After centrifuge for 5 min at 500×g at 4°C, cells were resuspended in cell freezing medium with 100 µg/mL CHX for dry ice transportation. Two replicates were prepared and the reads were processed and analyzed with the similar pipeline to the above mRNA half‐life profiling. To comply with the Ribo‐seq convention, the R1 reads were used for subsequent analysis. The Xtail software (v1.1.5) was utilized to measure the changes of translation efficiency with default parameter [61]. To ensure sufficient number of genes for subsequent analysis, a relaxed thresholds of 1.5‐fold change and *p* < 0.05 were applied. We also considered public Ribo‐seq data from GEO database for the machine learning modeling described later, including those from HeLa (GSE117299), HepG2 (GSE121952), Huh7 (GSE155447), and MOLM13 (GSE98623). These data were analyzed with the same pipeline.

### 4.8 High‐Throughput Profiling of Alternative Splicing Readouts

We exploited the ultra‐deep sequencing of 0 h samples as described in the above part a to measure alternative splicing events. Two replicates were prepared and the reads were processed and analyzed with the similar pipeline to the above mRNA half‐life profiling. The alternative splicing events and exon inclusion ratio (Psi) were calculated by rMATS‐turbo (v4.1.0, default parameter) [62]. A change of exon inclusion ratio (delta‐Psi) no less than 0.1, together with FDR < 0.05, were selected as the significant alternative splicing event.

### 4.9 Functional Enrichment Analysis

The functional enrichment of Gene Ontology (GO), Kyoto Encyclopedia of Genes and Genomes (KEGG), WikiPathways and MSigDB functional gene sets were performed by ClusterProfiler with default parameter and cutoffs [63]. Gene dependency score was obtained from DepMap database [27].

### 4.10 High‐Throughput Profiling of m^6^A Methylation Sites by GLORI Sequencing

Recently, Liu et al. developed GLORI, an antibody‐independent sequencing technique for single‐base quantitative detection of m^6^A methylation [24]. The main idea of GLORI is to find a catalytic system of glyoxal and nitrite through chemical reaction combination screening, which efficiently deaminate unmethylated adenosines to inosines (A‐to‐I), but not methylated ones. We used this state‐of‐the‐arts technique for the quantitative profiling of m^6^A methylation in A549, hESC and JURKAT. The GLORI‐identified m^6^A sites in HEK293T was directly obtained from the original study [24]. HUVEC was not included because of the required large amount of input cells by GLORI sequencing service. GLORI sequencing service was provided by Cloud‐Seq Biotech (Shanghai, China). Both the control and the *METTL3* knockdown cells were profiled for false positive control. Total RNA from the samples was extracted using TRIzol, and RNA was purified according to the MEGA clear transcription purification kit (Thermo fisher, catalog#AM1908). RNA was incubated with fragmentation buffer (NEB, catalog#E6150S) for 4 min at 94°C and glyoxal solution and DMSO were added, and the mixture was incubated for 30 min at 50°C in a preheated thermal cycler. minutes, and at the end of the incubation, the tubes were brought to room temperature, and then saturated H_3_BO_3_ (Sigma‐Aldrich, catalog#B0394) solution was added. The incubation was continued at 50°C for 30 min, and was then added to the pre‐conFig.d deamination buffer (750 mM NaNO_2_ (Sigma‐Aldrich, 31443), 40 mM MES (pH 6.0), and 10 µL of glyoxal solution for a total of 8 h of incubation. Finally, RNA was purified by ethanol precipitation, and sequencing libraries were constructed by GenSeq Low Input RNA Library Prep Kit (GenSeq, Inc.) following the process of the instructions provided by the manufacturer. The constructed sequencing libraries were subjected to quality control and quantification by BioAnalyzer 2100 system (Agilent Technologies, USA), followed by 150 bp paired‐end sequencing. Quality control of raw reads was first performed using Q30. Then cutadapt software (v1.9.3) was used for read de‐multiplex and removal of low‐quality reads. PCR duplicates were removed based on Unique Molecular Identifiers (UMI) tags. Clean reads were aligned to the reference genome (hg38) and BAM files were obtained using STAR (v2.7) and bowtie2 (v2.2.4) software. To ensure acceptable m^6^A site detection rate, we performed two rounds of sequencing, and the aligned BAM files from these two rounds were combined by samtools (v1.12). Finally, the m^6^A sites were called and quantified by GLORI‐tools [24] with parameter “–combine –rvs_fa ‐c 1 ‐C 0 ‐r 0.1 ‐p 0.05 ‐adp 0.05”. Only m^6^A sites that showed modification rate > 0.1 for both replicates were considered [24], and any m^6^A site identified from the *METTL3* knockdown cells were excluded from subsequent analyses.

### 4.11 Re‐Analysis of Public MeRIP‐seq Methylation Profiles

Currently, GLORI methylation profiles are not available for most cell lines. To obtain m^6^A sites for other cell types, we exploited widely used MeRIP‐seq‐based m^6^A methylation profiles [1, 2], To ensure comparable results, raw sequences of MeRIP‐seq studies were obtained from GEO (as specified in Table S4). The adapter trimming and quality control was performed by fastp, and the reads were aligned to human genome (hg38) by HISAT2. Finally, exomePeak2 was used to call m^6^A peaks [6, 64]. To obtain single‐nucleotide m^6^A sites, we compared m^6^A peaks with known single nucleotide m^6^A sites from m6A‐Atlas V2.0 database [6] and GLORI technique by using bedtools (v2.31.0). Sites following the consensus m^6^A motif DRACH were retained.

### 4.12 Obtaining RNA Binding Protein (RBP) Binding Site Annotations Through Re‐Analysis of Public Datasets

Our m^6^A readout prediction models were heavily relied on transcriptome‐wide annotation of RBP binding sites. CLIP‐seq and its derivates constitute the major source of such annotation. We obtained CLIP‐seq (and derivates)‐based RBP binding sites from POSTAR2 database [65]. We did not consider POSTAR3 because there was no RBP binding site score in the downloadable data of POSTAR3. Moreover, to make our annotations up‐to‐date, we obtained and re‐analyzed raw CLIP‐seq data from GEO, and adopted a pipeline similar to that of POSTAR database to process these data. Briefly, raw reads were filtered and trimmed by fastp and cutadapt software, and aligned to human genome (hg38) by using bowtie (v1.3.0, for short reads) or STAR (for longer reads). Samtools was used to process the aligned reads and the RBP binding regions were called by Piranha (v1.2.1) with recommended parameters [66]. We also obtained eCLIP‐based annotation from ENCODE projects [67]. We noted that the default merging of biological replicates in ENCODE would result in very limited binding sites for some RBPs. To this end, we also used bedtools to obtain the union of RBP binding regions derived from both replicates to enhance the sensitivity and transcriptome coverage. Finally, RBP site annotations from non‐CLIP techniques were directly obtained from the corresponding GEO accession. The summary of RBP binding site profiles was available in Table S5.

### 4.13 Feature Encoding for Machine Learning Model

For predicting half‐life and translation efficiency readouts, eleven set of features were considered: (1) The score of RBP binding site with the nearest distance on the genome; (2) The score of RBP binding site with the nearest distance on the transcripts; (3) The minimum RBP‐to‐m^6^A‐site distance among different m^6^A sites of the same gene on the genome; (4) The minimum RBP‐to‐m^6^A‐site distance on the transcripts; (5) The maximum RBP‐to‐m^6^A‐site distance on the genome; (6) The maximum RBP‐to‐m^6^A‐site distance on the transcripts; (7) The site to gene topology (e.g. minimum distance to start codon); (8) Tissue expression level, as measured by GTEx project [68]; (9) Gene dependency score, as record by DepMap database [27]; (10) Nearest distance to a 6‐mer on transcript; (11) Maximum counts of a specific k‐mer in ±100 nt range flanking known m^6^A sites. As for the prediction of exon inclusion ratio alterations, we used the annotation of the closest m^6^A site to the exon boundary to encode features (if any within 10 kb of exon boundary). Since only one representative m^6^A site was considered for each exon, no minimum/maximum operation of RBP features among multiple m^6^A sites on the same gene was required. Besides, as an exon‐level prediction, gene features like GTEx tissue expression and DepMap gene dependency score were also not included.

### 4.14 Machine Learning Model Training and Analysis

We exploited XGBoost [69] to model the readouts for each readout and each cell type. XGBoost has been successfully implemented in both bioinformatic prediction and clinical application [70, 71]. Tenfold cross‐validation to train and test the model, and the overall performance was measure by receiver operating characteristic (ROC) curve and area under ROC curve (AUC). A heuristic search of best combination of feature set was performed to measure the AUC gain of the feature set. And for RBP features, the feature importance score derived from the XGBoost models. We also employed SHAP method (available in the shapviz R package) to confirm the importance of the selected RBP features.

### 4.15 RNA Pull‐Down

The RNA oligonucleotides [ss‐A: 5’‐CGUCUCGGACUCGGACUGCU‐3’; ss‐m^6^A: 5’‐CGUCUCGG‐m^6^A‐CUCGG‐m^6^A‐CUGCU‐3’] with the same sequences of ss‐A and ss‐m^6^A were synthesized by Rui Biotech (Beijing, China). RNA pull‐down assay was conducted by the Pierce Magnetic RNA‐Protein Pull‐Down Kit (Thermo Fisher Scientific, catalog#20164). The included Thermo Scientific Pierce RNA 3’ Desthiobiotinylation Kit (catalog#20163) was used to label the target RNA. Next, biotin‐labeled RNA oligonucleotides containing adenosine or m^6^A (50 pmol) were immobilized onto 50 µL streptavidin magnetic beads in binding buffer at room temperature for 15–30 min. RNA‐conjugated streptavidin beads were then incubated with 200 µg total proteins from target cells in binding buffer in a final volume of 100 µL at 4°C for 30–60 min. RNA‐protein complex‐containing beads were washed three times with wash buffer. After extensive washing, RNA‐binding protein complexes were boiled and eluted in 1 × SDS loading buffer to separate on 10% SDS‐PAGE gels for western blot analysis.

### 4.16 RBP Binding Site Profiling by HyperTRIBE

HyperTRIBE is a promising high‐throughput sequencing technique for identifying potential binding site of an RBP. Leveraging plasmid fusing target RBP with hyperactive ADAR, the RNA editing signals close to the RBP binding site can be detected at transcriptome level through conventional RNA sequencing [32]. Here, the hyperactive ADAR containing TRIBE plasmids were constructed by Rui Biotech (Beijing, China). The control plasmid, mammalian expression plasmid, containing mCherry ADAR control and p2A GFP reporter can be obtained from Addgene (Plasmid #154786). As for RBP‐TRIBE plasmid, using standard restriction enzyme‐based cloning to clone known and putative m^6^A reader RBPs (YTHDF1, DDX6, FUBP3, FXR2 and L1TD1) into TRIBE plasmid. Sanger sequencing was used to confirm proper insertion and then control and RBP‐ADAR plasmids were infected into hESCs by transfection reagent jetOPTIMUS (Polyplus, catalog#101000051). After transient transfection into target cells for 24 h, the cells containing RBP‐ADAR fusion or ADAR alone proteins were enriched and expanded with 0.5 µg/ml puromycin. GFP^+^ cells then were sorted using flow cytometry to enhance the sensitivity of HyperTRIBE assays. Total mRNA was extracted using TRIzol reagent from the sorted cells. As for the sequencing and data analysis procedure, RNA‐sequencing for cells transfected with HyperTRIBE plasmid were conducted by Novogene Biotech (Tianjin, China). Messenger RNA was purified from total RNA using poly‐T oligo‐attached magnetic beads. After fragmentation, the first strand cDNA was synthesized using random hexamer primers followed by the second strand cDNA synthesis. After library quality control, quantified libraries were pooled and sequenced on Illumina NovaSeq with paired‐end 150‐bp reads, providing sequencing data at 40G per library. According to Biswas et al.'s protocol [72], first, cutadapt was used to remove the adaptor and low‐quality reads. Clean reads were then aligned to the reference genome (hg38) by using STAR software. Picard (v3.3.0) was used to remove duplicates and sort the output BAM files. To identify editing sites, we used Bullseye, a set of Perl scripts which was originally developed by Flamand et al. for analysis of editing events induced by transfected plasmids [9]. BAM files were parsed to generate coverage matrices at each position in the genome, excluding duplicate and multi‐mapped reads. Editing sites for HyperTRIBE in individual samples were then called by comparing A‐to‐I mutation rates at all positions within annotated exons (hg38) with samples expressing ADAR alone. Sites edited at rates between 5% and 99%, edited to higher levels than control cells (1.5 × for A2I), with at least two mutations, and that did not overlap annotated SNP were kept for further analysis. The final list of sites was identified as those found in three replicates and with an average editing rate of at least 5% in all samples where sites were called. To investigate the binding preference of the RBPs near m^6^A sites, BAM alignments were further processed using deepTools [73]. Heatmaps and profiles were generated using the plotHeatmap and plotProfile script from the deepTools suite.

### 4.17 RNA Electrophoretic Mobility Shift Assay (EMSA)

Two synthetic RNA probes were utilized: an unmodified sequence (ss‐A: 5′‐CGUCUCGGACUCGGACUGCU‐3′) and an m^6^A‐modified sequence (ss‐m^6^A: 5′‐CGUCUCGGCUCGGCUGCU‐3′). Recombinant GST‐tagged proteins (GST–DDX6, GST–L1TD1, GST–FRX2, and GST–FUBP3) were expressed and purified in vitro, then diluted to a working concentration of 1 µg/µL using RNase‐free water. Protein–RNA binding reactions were performed using the Chemiluminescent REMSA Kit (Beyotime, GS606). Each 10 µL binding reaction contained 1 µL (1 µg/µL) of protein and 1 µL (2.5 nM) of biotin‐labeled RNA probe in the supplied binding buffer. The mixture was incubated on ice for 10 min. Subsequently, complexes were resolved by non‐denaturing polyacrylamide gel electrophoresis at 100 V for 60 min. The RNA‐protein complexes were then transferred to a nitrocellulose membrane at 360 mA for 30 min using a wet transfer system. After transfer, the membrane was subjected to UV cross‐linking for 7 min to immobilize the RNA‐protein complexes. The membrane was blocked and then incubated with streptavidin–HRP conjugate for 20 min. Signal detection was performed using a chemiluminescent substrate according to the manufacturer's instructions.

### 4.18 CRISPR‐Mediated FUBP3, FXR2 and L1TD1 Knock Out (KO)

The CRISPR‐Cas9 knock‐out vector, containing cassettes for GFP, Cas9 endonuclease, and the double guide RNA (gRNA) moiety designed to target FUBP3, FXR2 and L1TD1, was custom synthesized by Guangzhou Regenverse Therapeutics Co., Ltd. (Guangzhou, China). After plasmid transfection for 48 h, GFP+ hESCs were sorted using flow cytometry, and plated at low density for single‐colony isolation. Selected single colonies were tested by western blot for loss of protein. Mutations were validated by sequencing products of PCR amplification of the regions flanking the targeting sites.

### 4.19 Cell Proliferation Evaluation

Cell counting kit 8 (CCK8) assay and alkaline phosphatase (ALP) staining assay were employed to evaluate cell proliferation. For CCK8 assay, hESCs were digested into single cell suspension using Accutase (Merck‐Millipore, catalog#SCR005) and the number of cells were counted by automated cell counter (RWD, catalog#C100). After counting, the cells were seeded in Matrigel‐coated 96‐well with a cell density of 2000 cells per well. After appropriate cultivate time, 10% CCK8 (Dojindo, catalog#CK04) was added into the culture medium and incubate cells for 2 h, then optical density value at 450 nm was detected using a microplate reader. Cells were detected in every 24 h. For ALP staining assay, hESCs were digested into single cell suspension using Accutase and the number of cells were counted by automated cell counter. After counting, the cells were seeded in matrigel‐coated 12‐well with the cell density of 1.2 × 10^5^ cells per well and incubated in mTeSR1 medium for 3 days. Then the cells were fixed with 4% paraformaldehyde for 3–5 min followed by washing with PBS. Next, the samples were added with BCIP/NBT staining solution (Beyotime, catalog#C3206) for 15 min in dark at room temperature. The usage of regents was followed with the manufacturer's recommendations. The cell images were taken after washing the cells with PBS.

### 4.20 hESCs Differentiation Assays

First, to initiate endodermal cell differentiation, hESCs were cultured on Matrigel‐coated 6‐well plate in RPMI1640 basal medium supplemented with B27 (Gibco, catalog#12587‐010) and 100 ng/mL Activin A (Peprotech, catalog#AF‐120‐14E‐10UG). After cell differentiation inducing for 3 days, endoderm markers were detected by flow cytometry and RT‐qPCR analysis. Moreover, for Hematopoietic mesodermal differentiation, hESCs were cultured to 60% density on Matrigel‐coated plates. Using Accutase to digest cells for 3–5 min, the cells were resuspended by blowing with mTeSR1 and seeded into ultra‐low attachment 6‐well plates with the cell density of 2× 10^5^ cells per well. Cells were cultured in a hypoxic incubator (5% CO_2_, 5% O_2_) for 24 h, until they formed to embryoid bodies (EB). To induce mesodermal differentiation, STEMdiff APELTM2 (STEMCELL, catalog#05270) was used as the basal medium, and the differentiation medium was conFig.d by adding corresponding cell factors according to the Kaufman hematopoietic differentiation protocol [74]. After cell differentiation inducing for 6 days, mesodermal markers were detected by flow cytometry and RT‐qPCR analysis. Finally, for neural ectodermal differentiation, monolayer culture protocol was used to initiate neural ectodermal differentiation. hESCs were plated onto Matrigel‐coated 6‐well plates with the cell density of 2 × 10^5^ cells/cm^2^, and then cultured in STEMdiff Neural Induction Medium (STEMCELL, catalog#08581) added with SMADi. After cell differentiation inducing for 3 days, neural ectodermal markers were detected by flow cytometry and RT‐qPCR analysis.

### 4.21 Statistical Analysis

Statistical analysis was performed by using R (v4.2.2) and GraphPad Prism (v8.0). Unless otherwise stated, bar charts represent the mean ± standard deviation of the mean (S.D,), and the results for the two groups were compared using a two‐tailed unpaired Student's *t*‐test. A *p*‐value smaller than 0.05 was considered significant unless stated differently, and the exact degree of significance as indicated by asterisks is stated in the legends. Statistical significance was presented as *p < 0.05, **p < 0.01, ***p < 0.001 and ****p < 0.0001; ns indicates *p* > 0.05.

## Author Contributions

Conceptualization, Y.Z. and Y.L.; Methodology: Y.Z., Y.L., Z.H., W.Z., R.L. and Z.W.; Data curation: Y.Z., R.L. and W.Z.; Resources: Z.H., J.H., W.K., Q.C. and Y.L.; Investigation: Z.H., R.L., Z.W. J.H., J.W. and T.Z.; Formal analysis: Y.Z., R.L., W.Z. and Z.H.; Validation: R.L., Z.W., J.W. and T.Z.; Software: Y.Z. and R.F.; Supervision: Y.Z., Y.L., W.K. and Q.C.; Funding acquisition: Y.Z., Y.L. and Q.C.; Writing – original draft: Y.Z., R.L. and Z.H.; Writing – review & editing: Y.Z. and Y.L.

## Funding

This study was supported by the National Natural Science Foundation of China (32222020 and 32070658 to Y.Z., 32371533 to Y.L.), Michigan Medicine‐PKUHSC Joint Institute for Translational and Clinical Research (BMU2023JI002 to Y.L.), PKU‐Baidu Fund (2019BD014 to Q.C.).

## Conflicts of Interest

The authors declare no conflicts of interest.

## Ethics Approval Statement

The authors have nothing to report.

## Patient Consent Statement

The authors have nothing to report.

## Permission to Reproduce Material from Other Sources

The authors have nothing to report.

## Clinical Trial Registration

The authors have nothing to report.

## Supporting information




**Supporting File**: advs73946‐sup‐0001‐SuppMat.docx.

## Data Availability

The data that support the findings of this study are openly available in [Gene Expression Omnibus] at [https://www.ncbi.nlm.nih.gov/geo], reference number [179872].
