# Supplementary material for: Comprehensive Profiling of N 6‐methyladnosine (m6A) Readouts Reveals Novel m6A Readers That Regulate Human Embryonic Stem Cell Differentiation
Source: Adv Sci (Weinh). 2026 Jan 20;13(18):e10075. doi: 10.1002/advs.202510075 (PMC13042514; doi:10.1002/advs.202510075)
Supplement: Supplementary file 1 — Supporting File: advs73946‐sup‐0001‐SuppMat.docx. [file ADVS-13-e10075-s001.docx]

Supporting Information

Comprehensive Profiling of *N*^6^-methyladnosine (m^6^A) Readouts Reveals Novel m^6^A Readers that Regulate Human Embryonic Stem Cell Differentiation

Zhou Huang, Rucong Liu, Zibaguli Wubulikasimu, Wanqing Zhao, Jiaqi Huang, Jiaxuan Wang, Tianyuan Zhang, Rui Fan, Wei Kong, Qinghua Cui, Yang Li*, and Yuan Zhou*

**
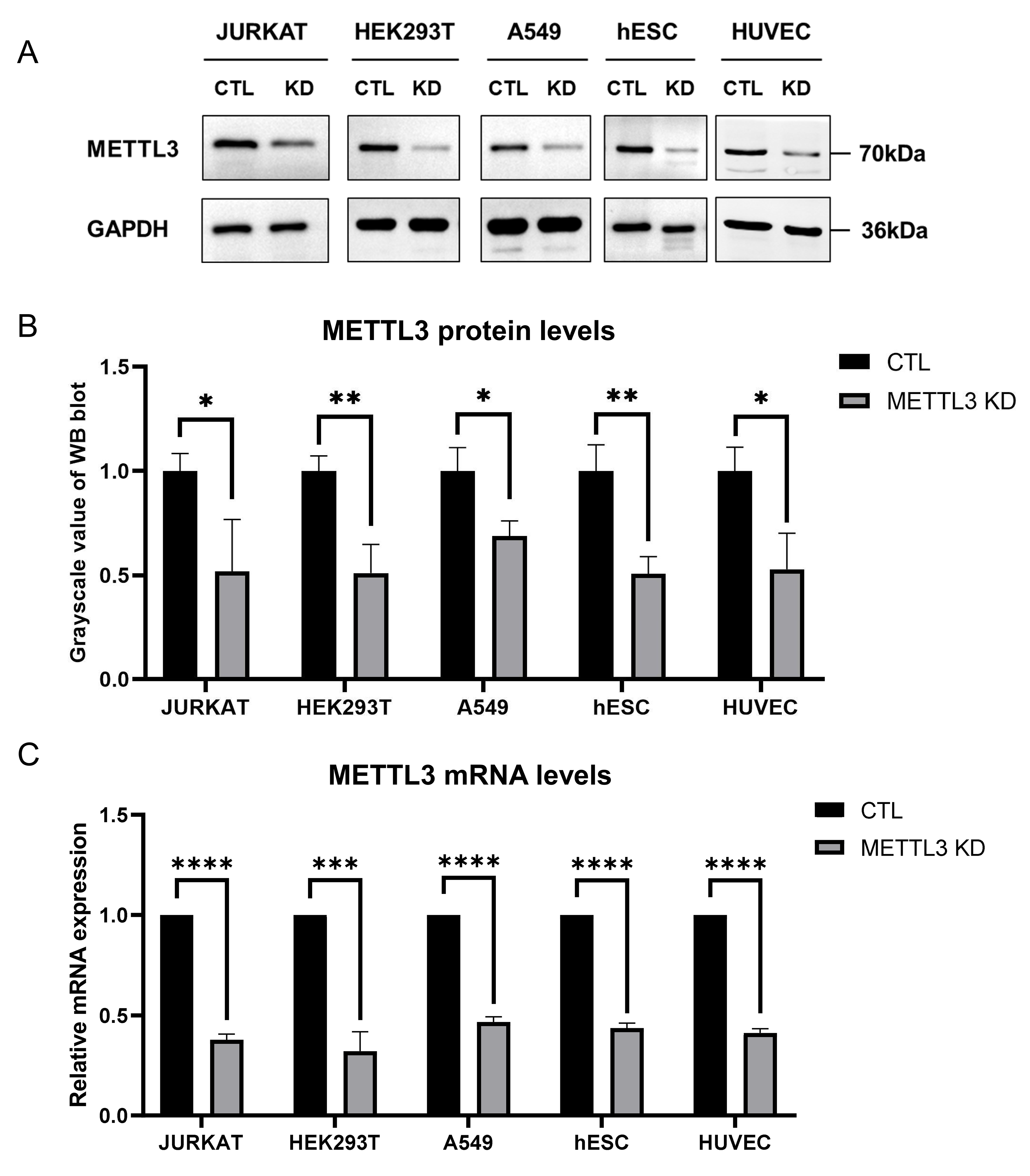
**

**Supplementary Figure S1. Verification of *METTL3* knockdown effects.**

(**A**) Western blot analysis for expression of METTL3 in shControl and METTL3 KD cells. (**B**) The statistics of grayscale value of western blot. (**C**) RT-qPCR analysis for the relative expression of *METTL3* mRNA in shControl and METTL3 KD cells. Data in (**B**) and (**C**) were statistically analyzed using two-tailed unpaired Student's t-test. Error bars represent mean ± SD (n = 3 independent experiments). For all statistical plots, * means P < 0.05, ** means P < 0.01, **** means P < 0.0001.


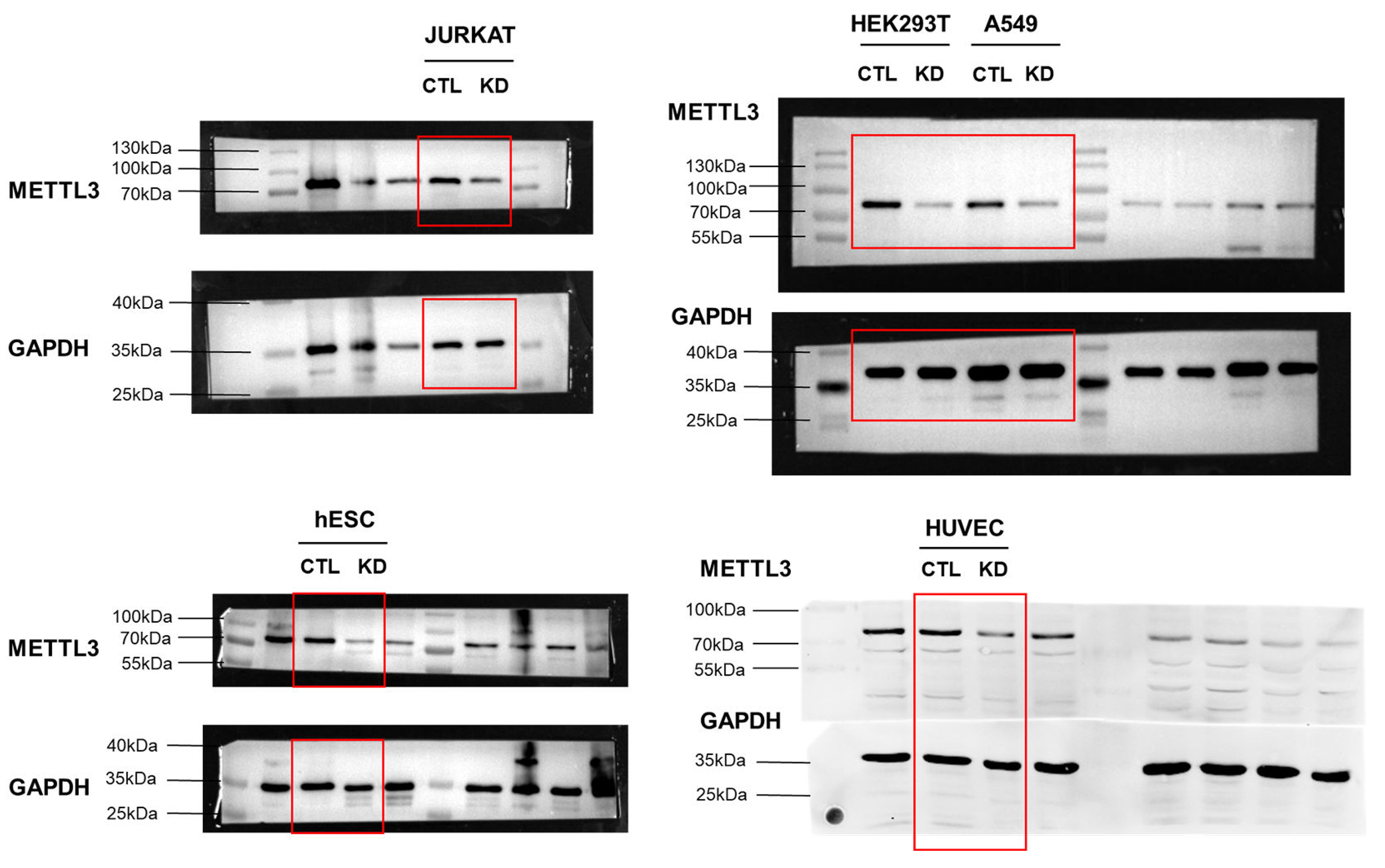


**Supplementary Figure S2. Source images of Western blot assay verifying *METTL3* knockdown effects.**

Source gel images of Supplementary Figure S1 is shown.


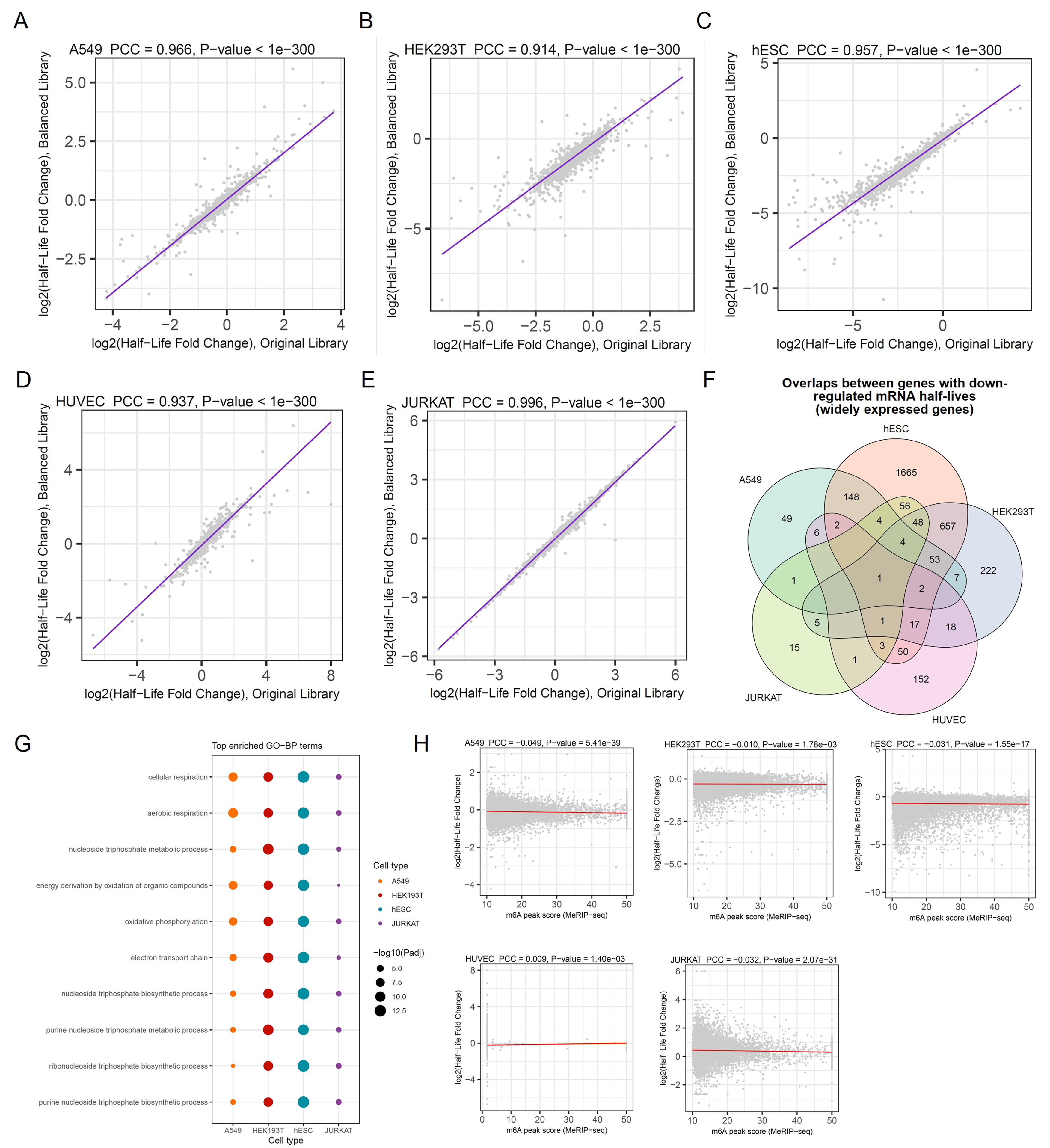


**Supplementary Figure S3.** **Robustness of half-life readout measurements.**

(**A-E**) Correlation between the log2(fold change) of mRNA half-life (control versus *METTL3* knockdown comparison) estimated from the original library and those estimated from the balanced library for A549 (**A**), HEK293T (**B**), hESC (**C**), HUVEC (**D**), JURKAT (**E**). The balanced libraries were generated by randomly down-sampling the sequencing library of 0h to the same depth of the sequencing library of the other time points. (**F**) Venn diagram of half-life down-regulated genes in different cell types, with only genes widely expressed in all of the five cell types are considered here. (**G**) Top 10 enriched Gene Ontology Biological Process (GO-BP) terms of half-life down-regulated genes among different cell types sorted by total -log10(adjusted P-value). (**H**) Correlation between fold change in half-life and m^6^A methylation levels assessed by MeRIP-seq. Data in (**A-E and H**) and (**G**) were statistically analyzed using correlation test and Fisher exact test, respectively.


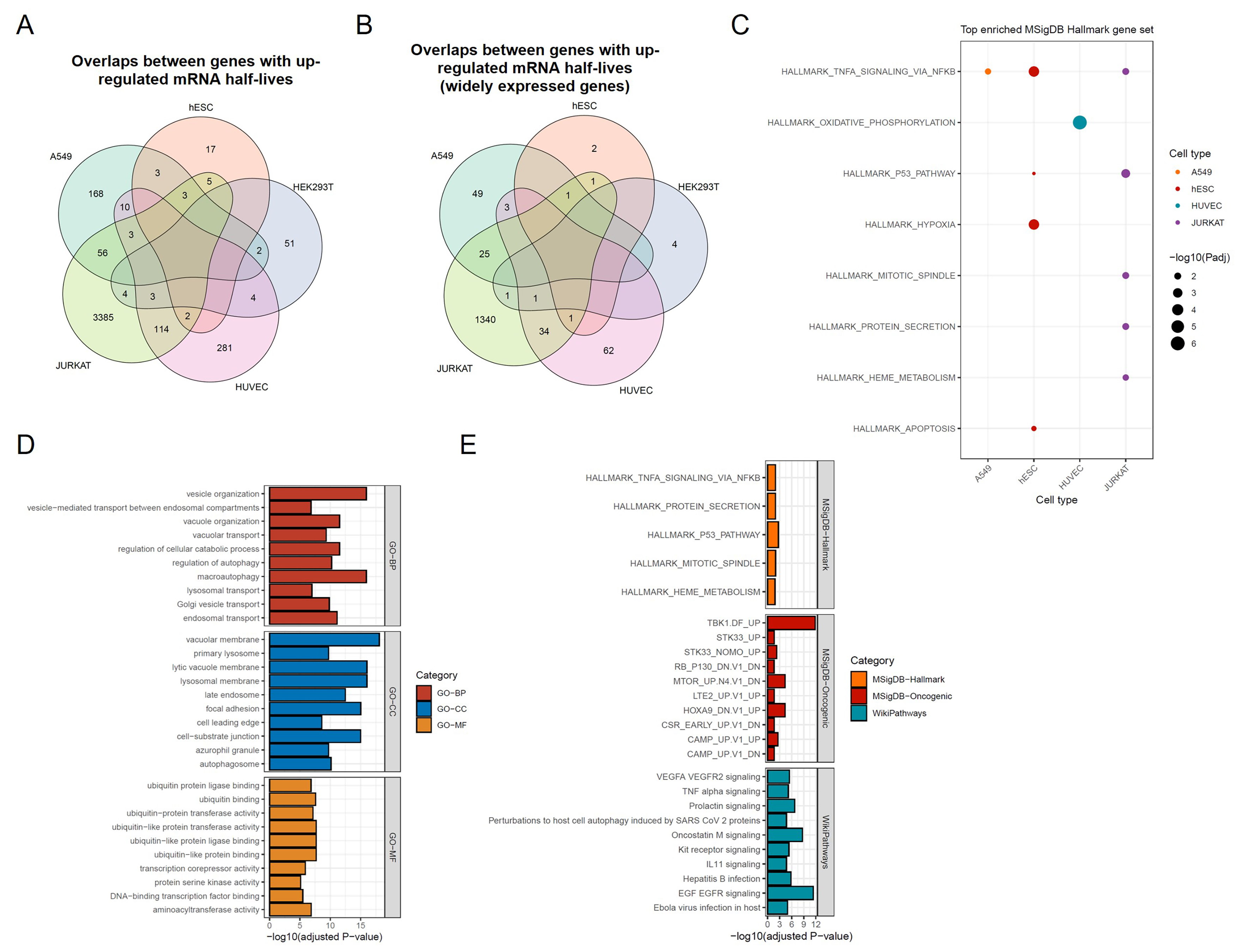


**Supplementary Figure S4.** **Overview of mRNA half-life up-regulated genes.**

(**A**) Venn diagram of half-life down-regulated genes in different cell types. (**B**) Venn diagram of half-life down-regulated genes in different cell types, with only genes widely expressed in all of the five cell types are considered here. (**C**) Top 10 enriched MSigDB Hallmark gene sets of half-life down-regulated genes among different cell types, sorted by total -log10(adjusted P-value). (**D**) Top enriched GO functional terms for half-life down-regulated genes in JURKAT cells. (**E**) Top enriched pathways and gene sets for half-life down-regulated genes in JURKAT cells. Data in (**C-E**) were statistically analyzed using Fisher exact test.


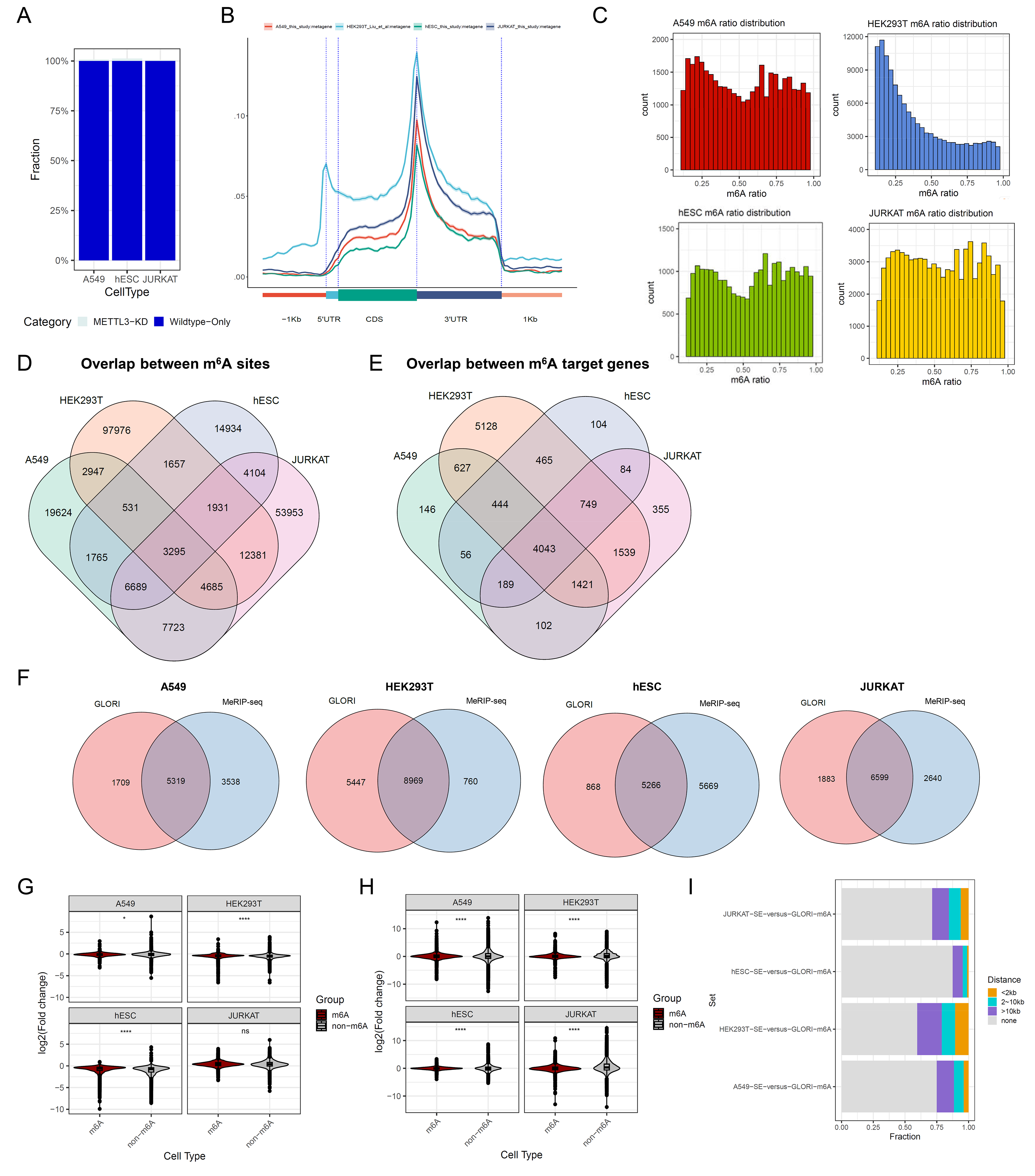


**Supplementary Figure S5.** **Overview of m^6^A modification sites identified by GLORI.**

(**A**) Fraction of false positive m^6^A sites that are also identified from the *METTL3* knockdown GLORI samples among the m^6^A sites identified from the wildtype control GLORI samples. (**B**) Metagene plot showing the distribution of GLORI-identified m^6^A modification sites in the target genes. (**C**) Histogram illustration of m^6^A modification ratio estimated by GLORI. (**D**) Venn diagram showing the overlap of m^6^A modification sites between different cell types. (**E**) Venn diagram showing the overlap of m^6^A target genes between different cell types. (**F**) Venn diagram showing the overlap between m^6^A target genes identified by GLORI and those identified by MeRIP-seq in the corresponding cell types. (**G**) Violin plots comparing the log2(fold change) of mRNA half-life in m^6^A-postive and m^6^A-negative groups, as per the m^6^A methylation sites identified by GLORI on the corresponding cell types. (**H**) Violin plots comparing the log2(fold change) of translation efficiency in m^6^A-postive and m^6^A-negative groups, as per the m^6^A methylation sites identified by GLORI on the corresponding cell types. (**I**) Percentage of exons showing exon inclusion ratio changes with m^6^A methylation sites identified by GLORI on the corresponding cell types near their exon boundaries. Data in (**G**) and (**H**) were statistically analyzed using two-tailed unpaired Student's t-test. * means P < 0.05, **** means P < 0.0001, ns means not significant. For all box plots, boxes cover Q1 to Q3, while whiskers extend to 1.5 IQR.


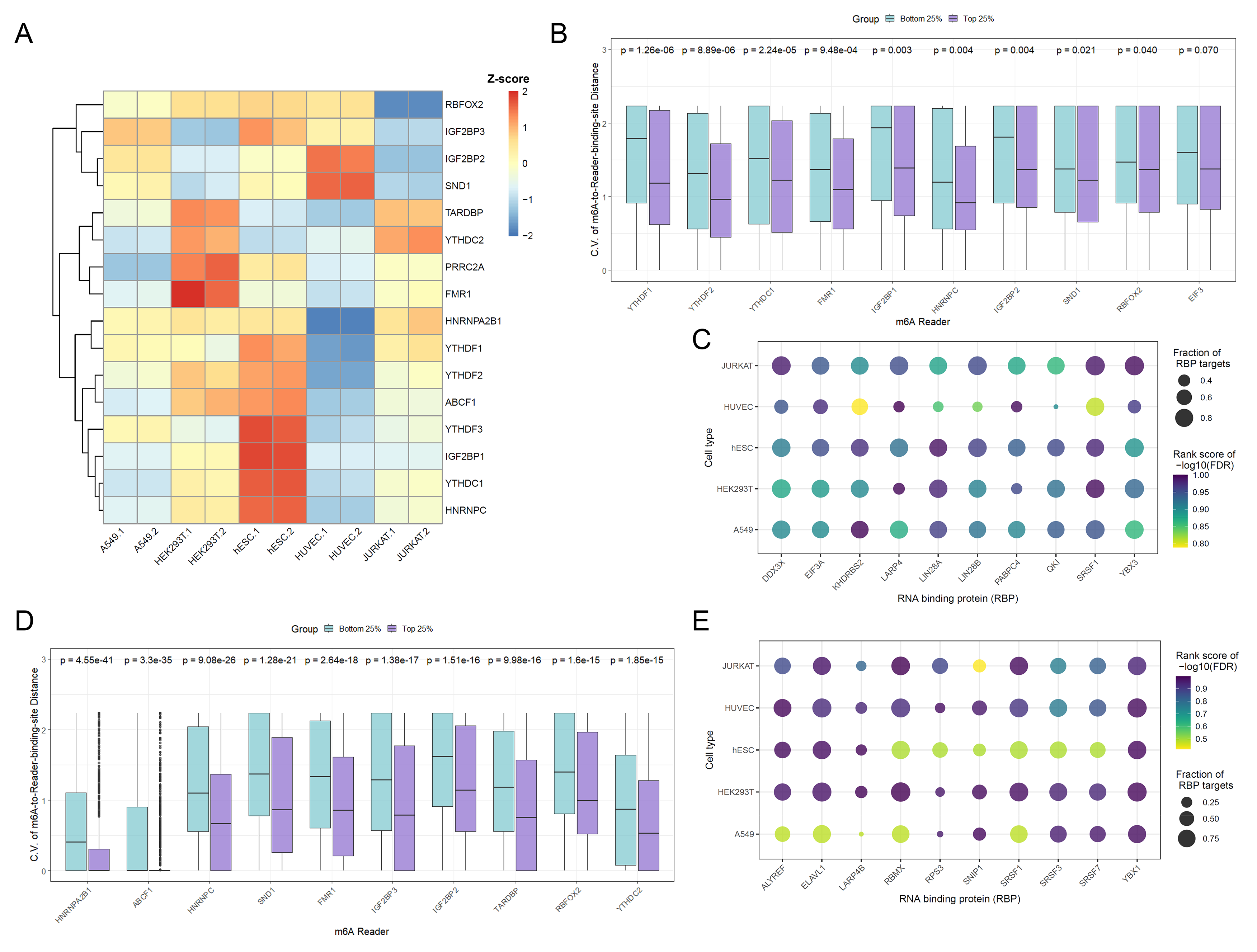


**Supplementary Figure S6.** **Association between mRNA half-life and translation efficiency changes and the RNA binding protein context.**

(**A**) Heatmap comparing gene expression levels of known m^6^A readers across the five cell types. (**B**) Boxplot comparing coefficient of variation (C.V.) of m^6^A site-to-m^6^A reader binding site distances across the five cell types between genes showing high and low variability of mRNA half-life changes, as estimated by the standard deviation (S.D.) of log2(fold change). (**C**) Bubble plot summarizing the top RNA binding proteins associated with mRNA half-life changes in the m^6^A negative group. (D) Boxplot comparing C.V. of m^6^A site-to-m^6^A reader binding site distances across the five cell types between genes showing high and low variability of translation efficiency changes. (**E**) Bubble plot summarizing the top RNA binding proteins associated with translation efficiency changes in the m^6^A negative group. Wilcoxon test was applied for data in (**B**) and (**D**). Fisher exact test was applied for data in (**C**) and (**E**). For all box plots, boxes cover Q1 to Q3, while whiskers extend to 1.5 IQR.


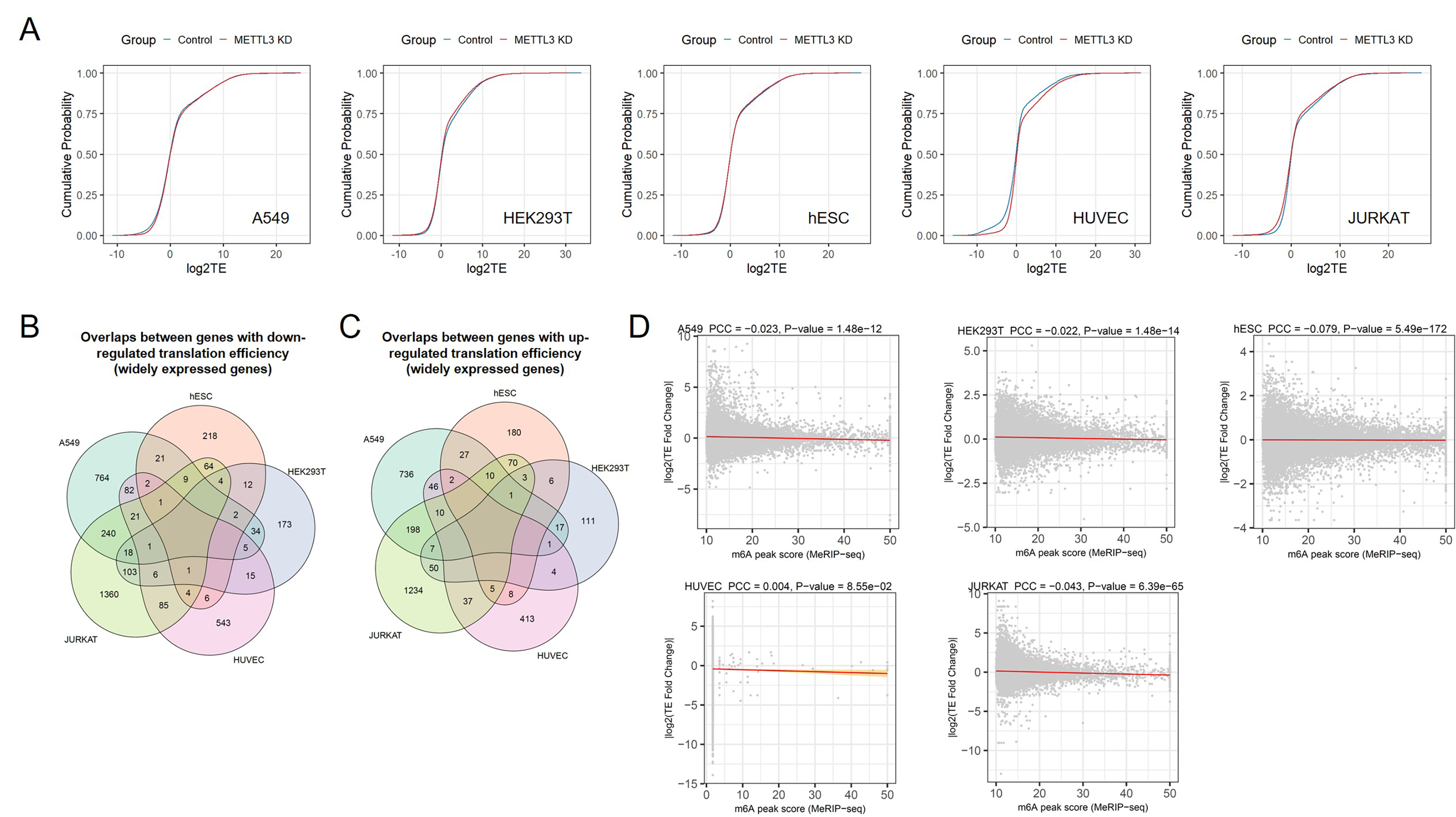


**Supplementary Figure S7.** **Overview of changes in translation efficiency in control-versus-METTL3 knockdown cell comparison.**

(**A**) Cumulative distribution plot of m^6^A-mediated changes in translation efficiency. The comparison was performed between m^6^A-normal cells (shControl) versus m^6^A-disrupted cells (shMETTL3) as the background. (**B-C**) Venn diagram of translation efficiency down-regulated (**B**) and up-regulated (**C**) genes in different cell types, with only genes widely expressed in all of the five cell types are considered here. (**D**) Correlation between fold change in half-life and m^6^A methylation levels assessed by MeRIP-seq. Correlation test was applied for data in (**D**).


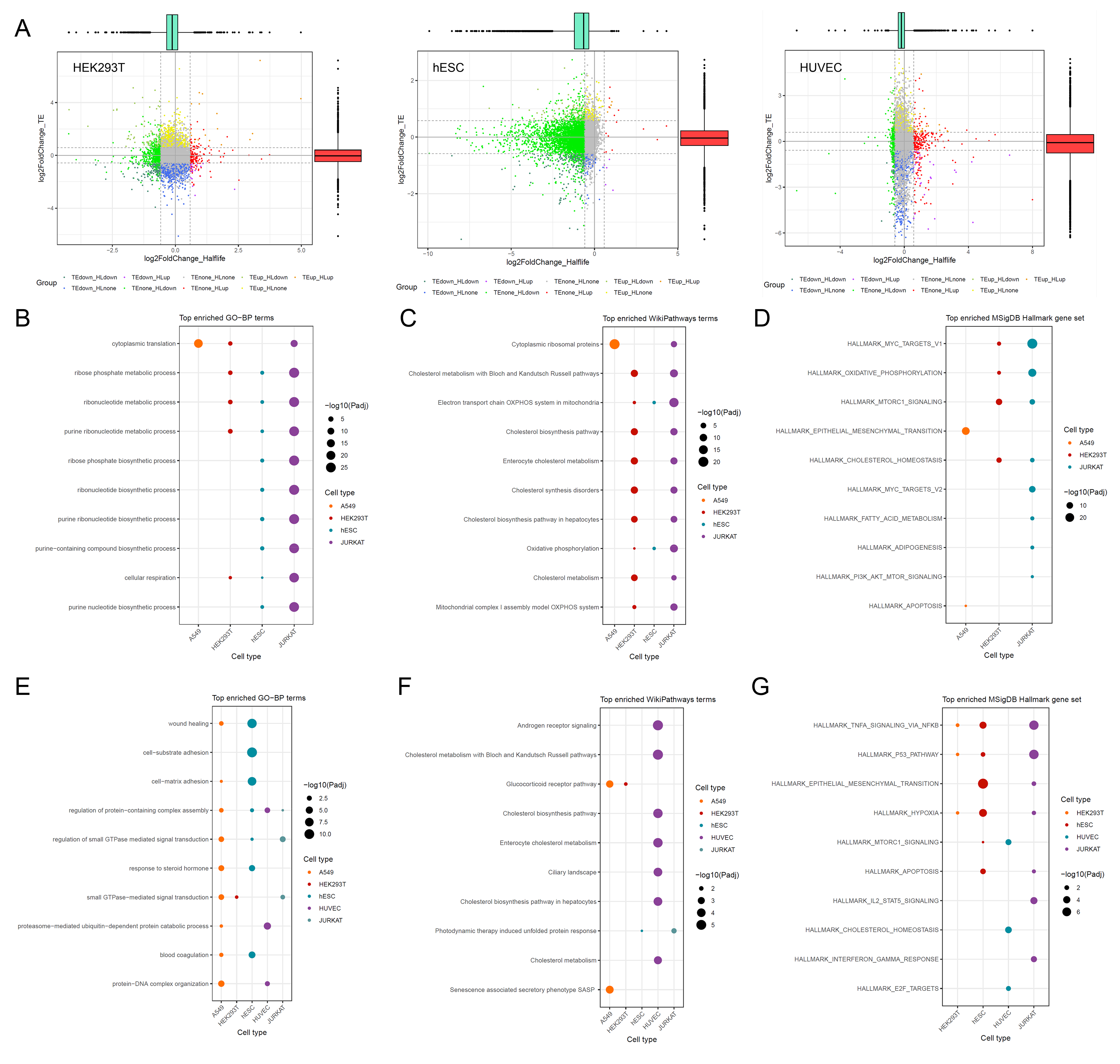


**Supplementary Figure S8.** **Distribution and functional association of genes showing m^6^A-mediated translation efficiency changes.**

(**A-C**) Scatter plot showing translation efficiency versus half-life changes in HEK293T (**A**), hESC (**B**) and HUVEC cells (**C**). (**D**) Top 10 enriched Gene Ontology-Biological Process (GO-BP) functional terms for translation efficiency down-regulated genes. (**E**) Top 10 enriched WikiPathways pathways for translation efficiency down-regulated genes. (**F**) Top 10 enriched MSigDB Hallmark gene set for translation efficiency down-regulated genes. (**G-I**) The corresponding results for translation efficiency up-regulated genes. Fisher exact test was applied for data in (**D-I**). For all box plots, boxes cover Q1 to Q3, while whiskers extend to 1.5 IQR.


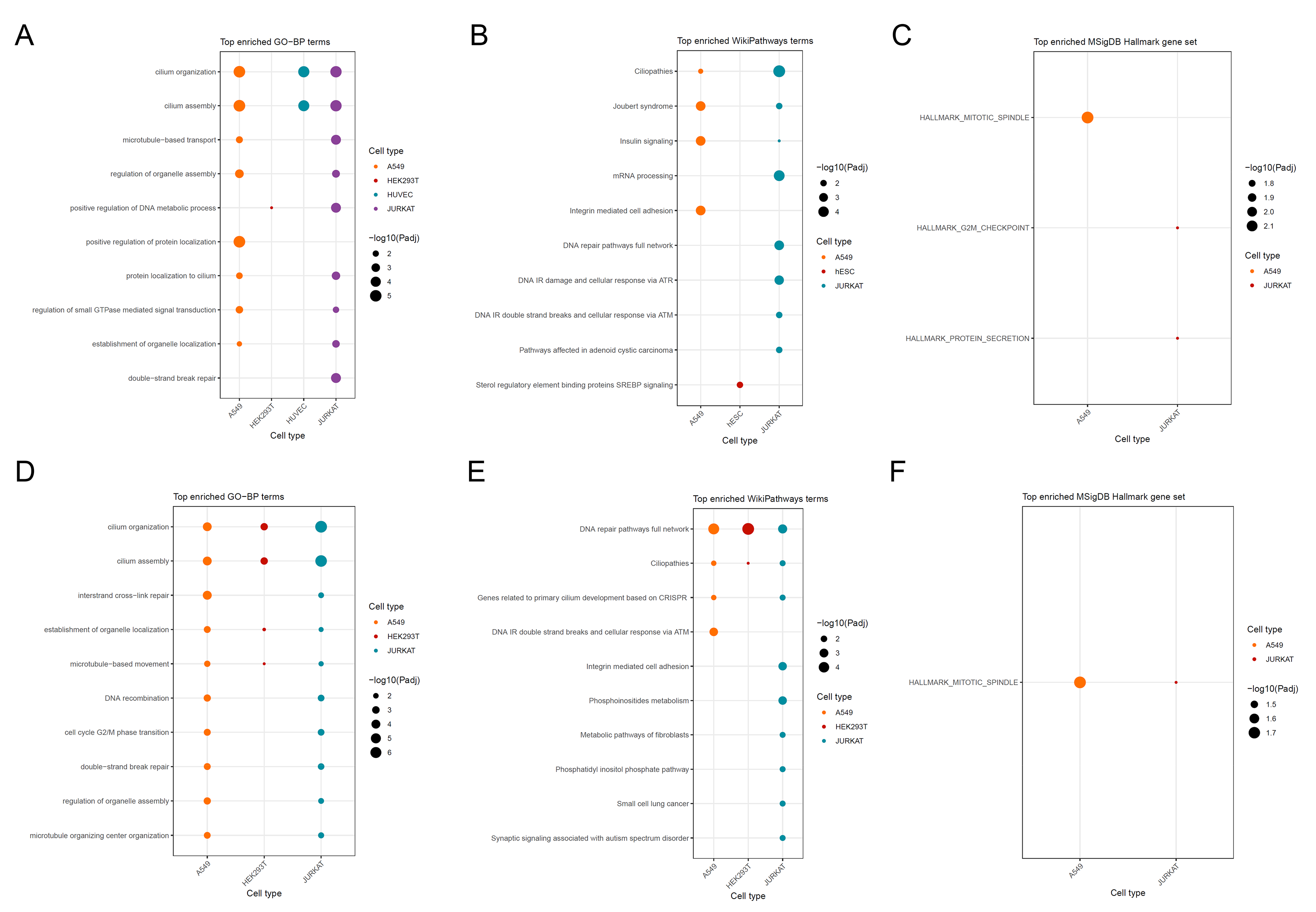


**Supplementary Figure S9. Functional association of genes with exon skipping events.**

(**A**) Top 10 enriched GO-BP functional terms for exon inclusion ratio down-regulated genes. (**B**) Top 10 enriched WikiPathways pathways for exon inclusion ratio down-regulated genes. (**C**) Top 10 enriched MSigDB Hallmark gene set for exon inclusion ratio down-regulated genes. (**D-F**) The corresponding results for exon inclusion ratio up-regulated genes. Fisher exact test was applied for data in (**A-F**).

**
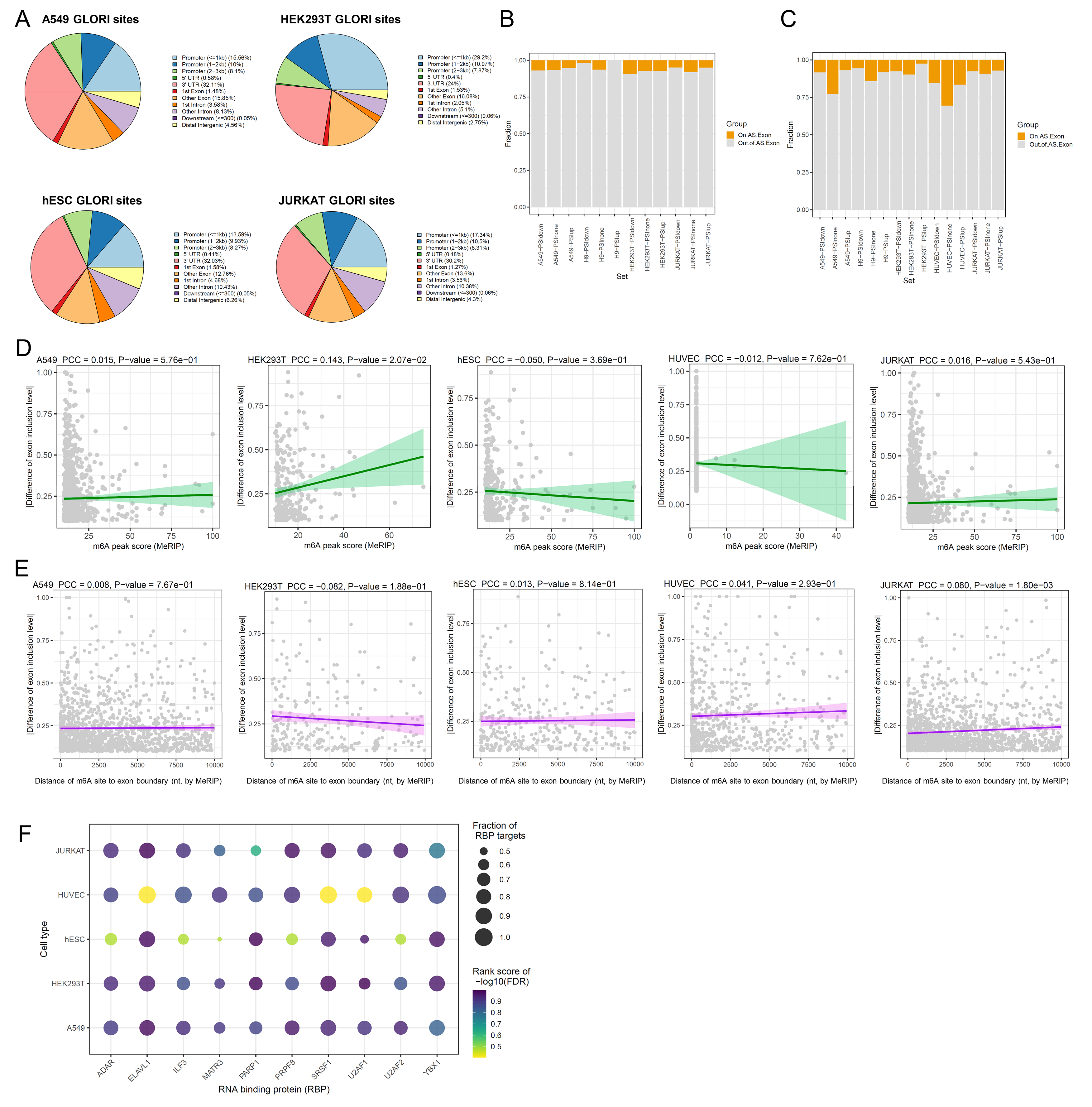
**

**Supplementary Figure S10. Investigation of the possible association between exon inclusion ratio changes and m^6^A sites.**

(**A**) Distribution of GLORI-identified m^6^A sites across different gene elements. (**B-C**) Fraction of exons harboring m^6^A sites among exons showing changes in exon inclusion ratio, as estimated using m^6^A site data from GLORI (**B**) or MeRIP-seq (**C**). (**D**) Correlation between changes in exon inclusion ratio (in absolute values) and m^6^A (within 10 kb to exon boundary) methylation levels assessed by MeRIP-seq. (**E**) Correlation between changes in exon inclusion ratio (in absolute values) and the distances from m^6^A methylation sites to exon boundaries. Correlation test was applied for data in (**D-E**). (**F**) Bubble plot summarizing the top RNA binding proteins associated with exon inclusion ratio changes in the m^6^A negative group, Fisher exact test applied for data in (**F**).


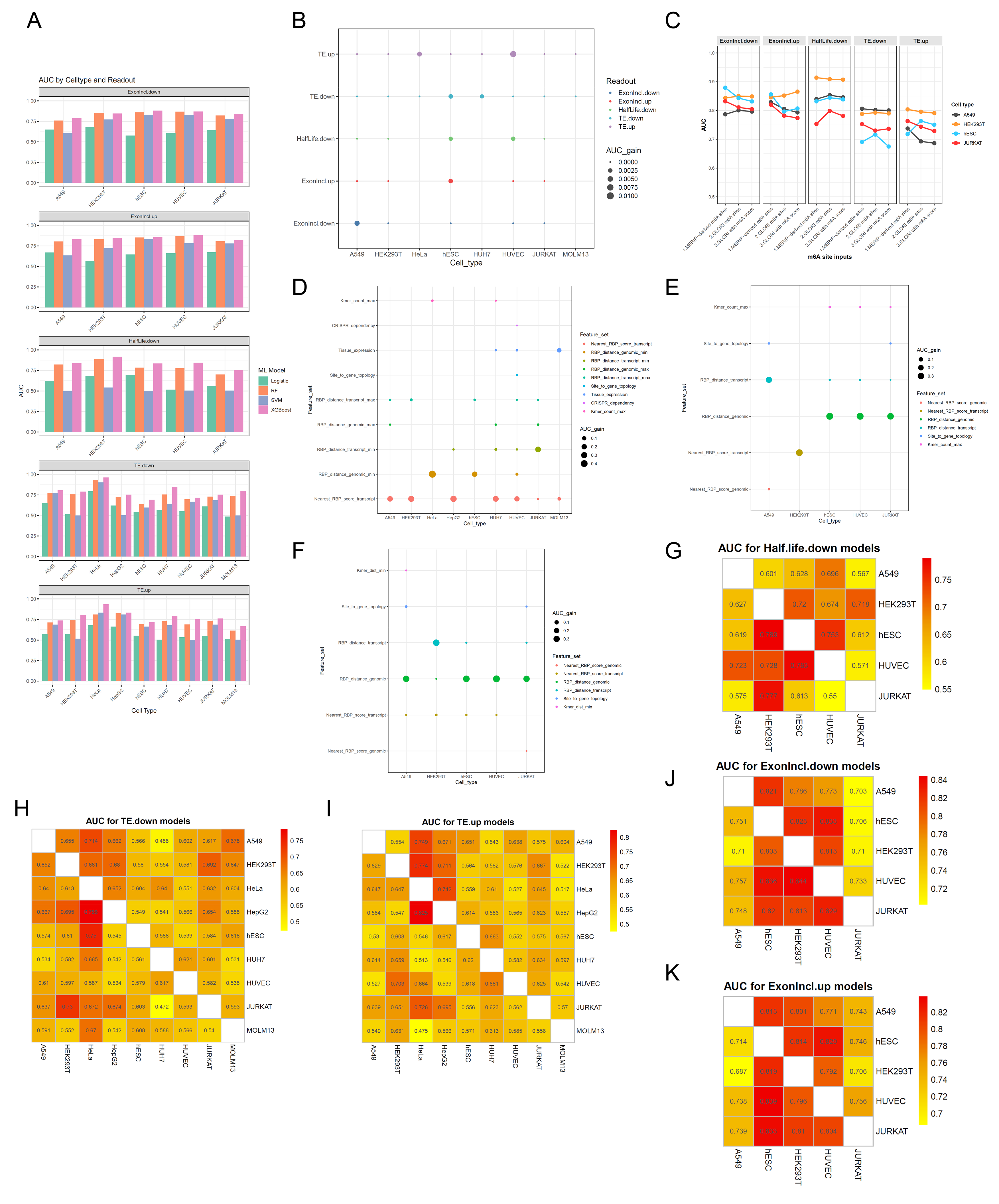


**Supplementary Figure S11. Ablation experiments, feature set contribution, and cross-cell-type prediction performance of the machine learning predication models of m^6^A readouts.**

(**A**) Comparison between XGBoost and other classical machine learning models in predicting different categories of m^6^A readouts. The classical machine learning models here include random forest (RF), logistic regression (Logistic) and support vector machine (SVM). ExonIncl means exon inclusion ratio; TE means translation efficiency. (**B**) Bubble plot summarizing the gain of AUC after parameter optimization of the XGBoost model. (**C**) Line plot comparing the performance of XGBoost models with MeRIP-seq-derived m^6^A sites, GLORI-derived m^6^A sites or GLORI-derived m^6^A sites plus the corresponding m^6^A methylation rate. (**D**) Bubble plot summarizing the performance contributions of different feature sets for predicting translation efficiency up-regulated genes. (**E**) Bubble plot summarizing the performance contributions of different feature sets for predicting exon inclusion ratio down-regulated exons. (**F**) Bubble plot summarizing the performance contributions of different feature sets for predicting exon inclusion ratio up-regulated exons. (**G-K**) Heatmap summarizing the cross-cell-type prediction performance of the models for predicting half-life down-regulation genes (**G**), translation efficiency down-regulated genes (**H**), translation efficiency up-regulated genes (**I**), exon inclusion ratio down-regulated exons (**J**), and exon inclusion ratio up-regulated exons (**K**). Rows represent the source cell type of testing data, while columns represent the cell type for training predictive model.

**
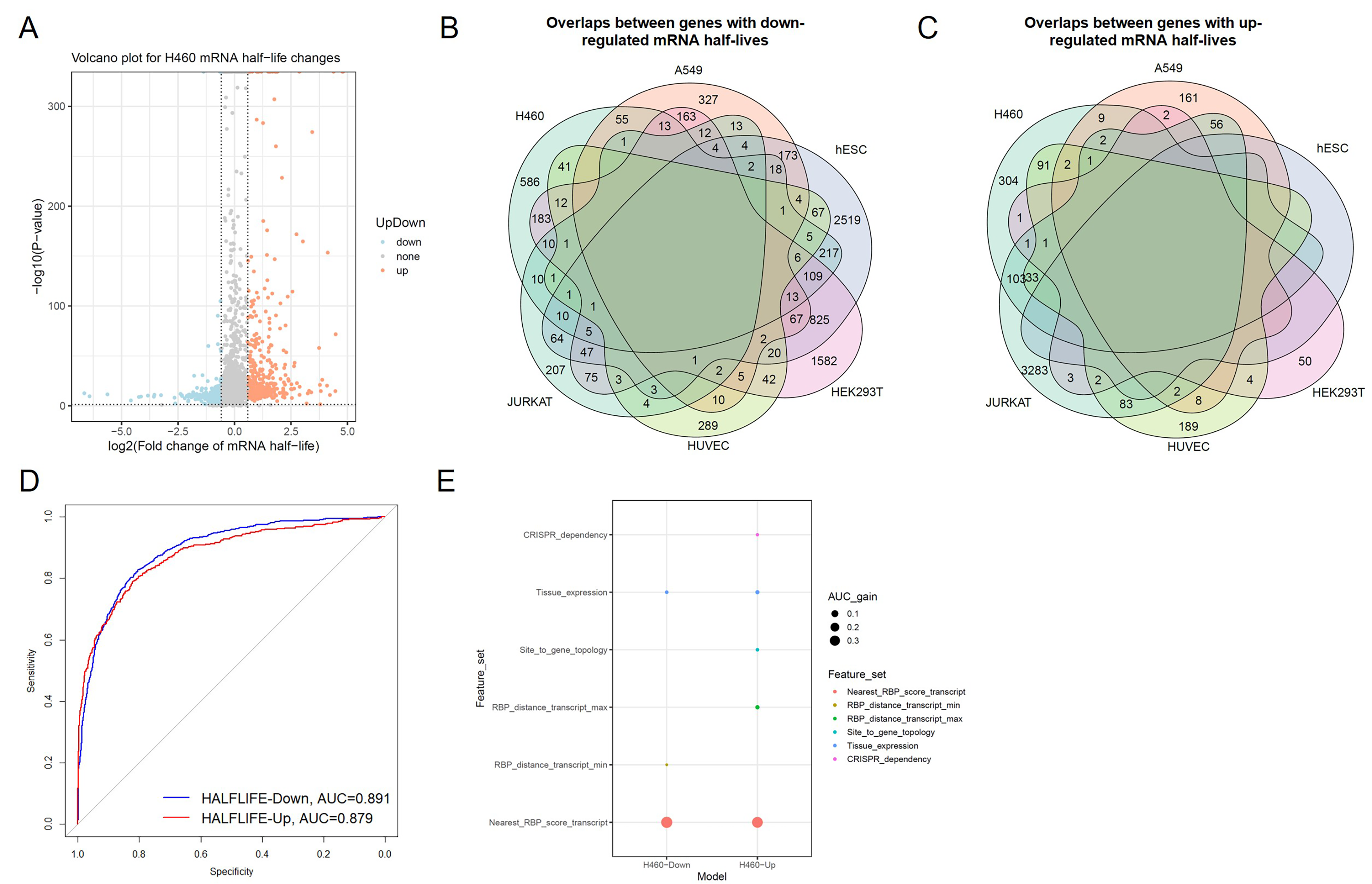
**

**Supplementary Figure S12. Half-life readout profiling and prediction in H460 cells treated by METTL3 inhibitor STM2457.**

(**A**) Volcano plot of m^6^A-mediated changes in mRNA half-lives. The comparison was performed between m^6^A-normal cells (Control) versus m^6^A-disrupted cells (METTL3 inhibitor treated) as the background. (**B-C**) Venn diagram of half-life down-regulated (**B**) and up-regulated (**C**) genes in different cell types. (**D**) ROC curve assessing the performances of machine learning models for predicting the m^6^A readout effects in terms of half-life down- and up-regulated genes. (**E**) Bubble plot summarizing the performance contributions of different feature sets for predicting half-life down- and up-regulated genes.

**
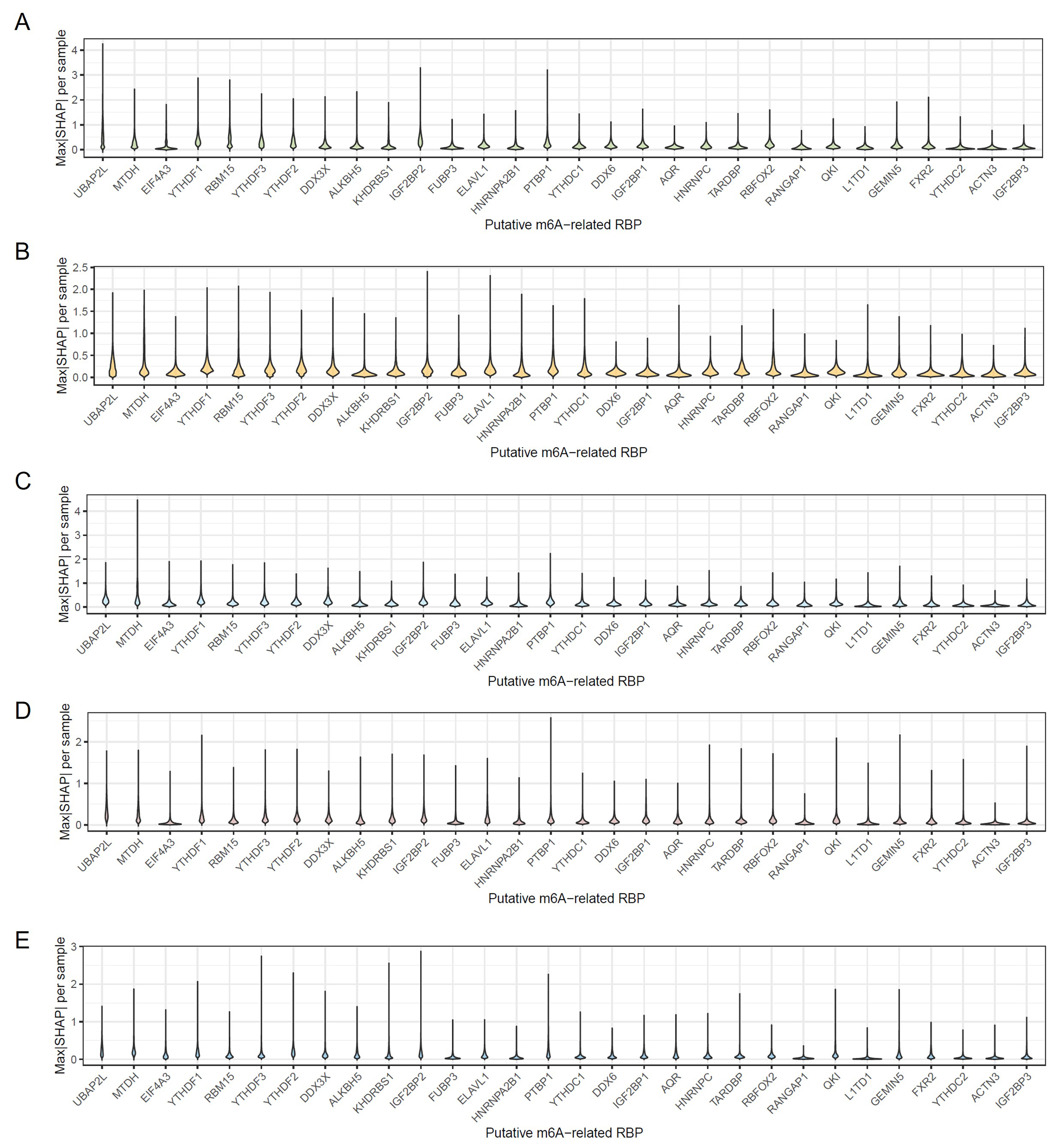
**

**Supplementary Figure S13. Validation of the RBP feature importance by Shapley additive explanations (SHAP) method.**

The distribution of per sample maximum absolute SHAP values are summarized for models predicting half-life down-regulation genes (**A**), translation efficiency down-regulated genes (**B**), translation efficiency up-regulated genes (**C**), exon inclusion ratio down-regulated exons (**D**), and exon inclusion ratio up-regulated exons (**E**).


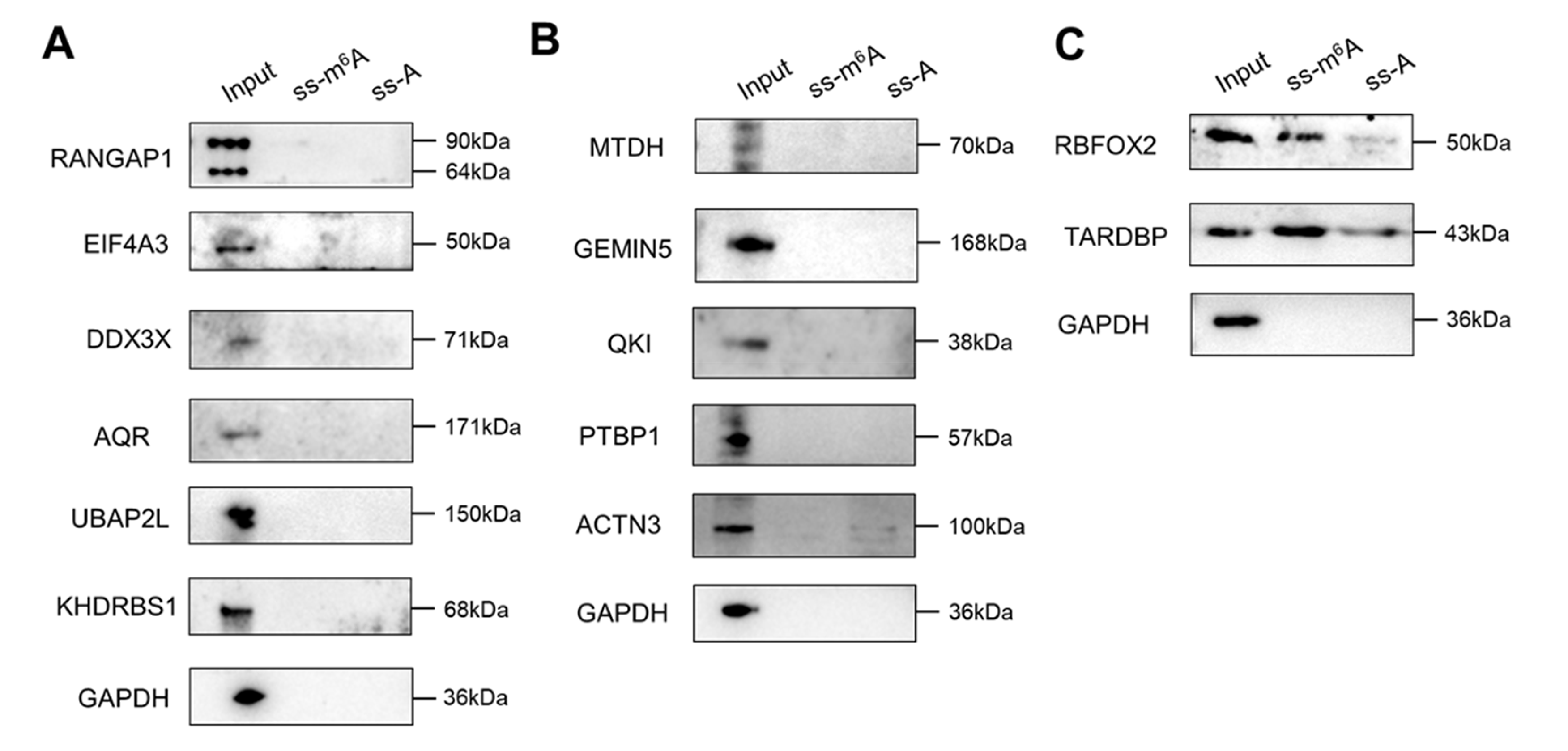


**Supplementary Figure S14. Screening of m^6^A binding proteins by RNA pull-down assay.**

Three batches of RNA pull-down assay were performed. (**A**) RNA pull-down assay batch 1. (**B**) RNA pull-down assay batch 2. (**C**) RNA pull-down assay batch 3. RBFOX2 and TARDBP (also known as TDP43) are known m^6^A readers that serve as positive controls here.

**
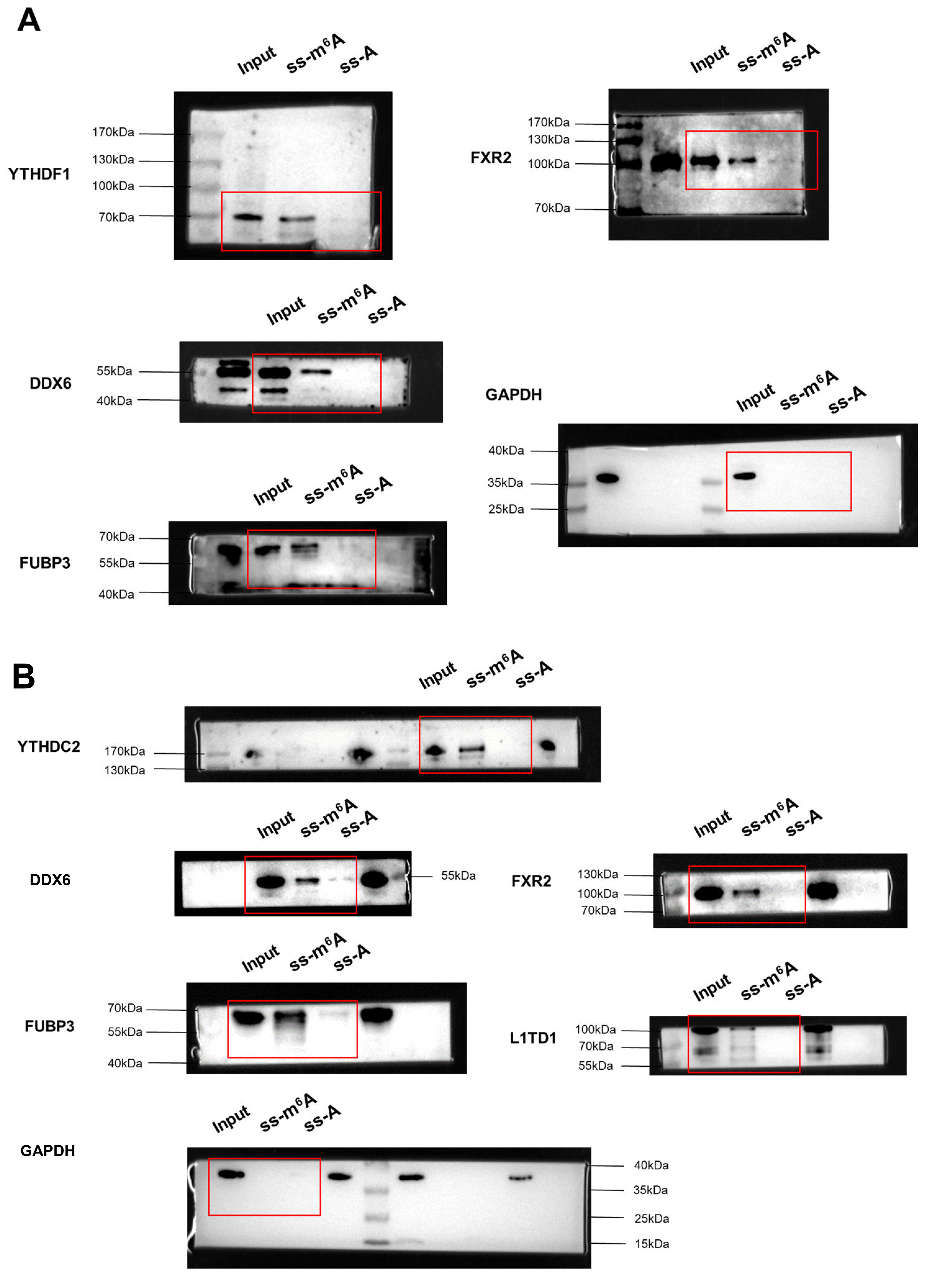
**

**Supplementary Figure S15. Source images of RNA pull-down assay performed in A549 cells and hESCs.**

(A) Source gel images of Figure 5A are shown. (B) Source gel images of Figure 5B are shown.


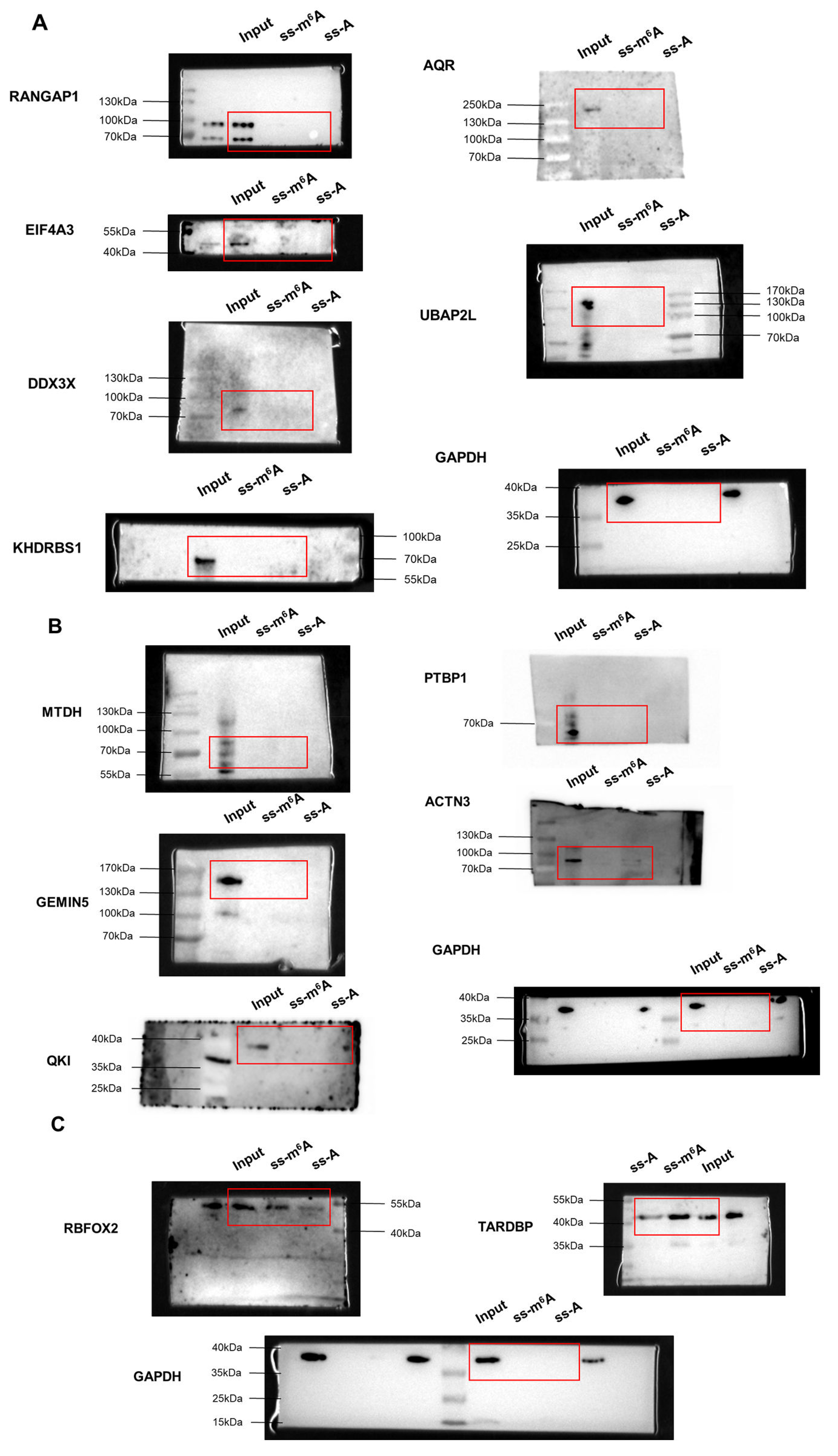


**Supplementary Figure S16. Source images of RNA pull-down assay performed in A549 cells, additional.**

Three batches of RNA pull-down assay were performed. (**A**) Source gel images of RNA pull-down assay batch 1 (Supplementary Figure S14A) are shown. (**B**) Source gel images of RNA pull-down assay batch 2 (Supplementary Figure S14B) are shown. (**C**) Source gel images of RNA pull-down assay batch 1 (Supplementary Figure S14C) are shown.


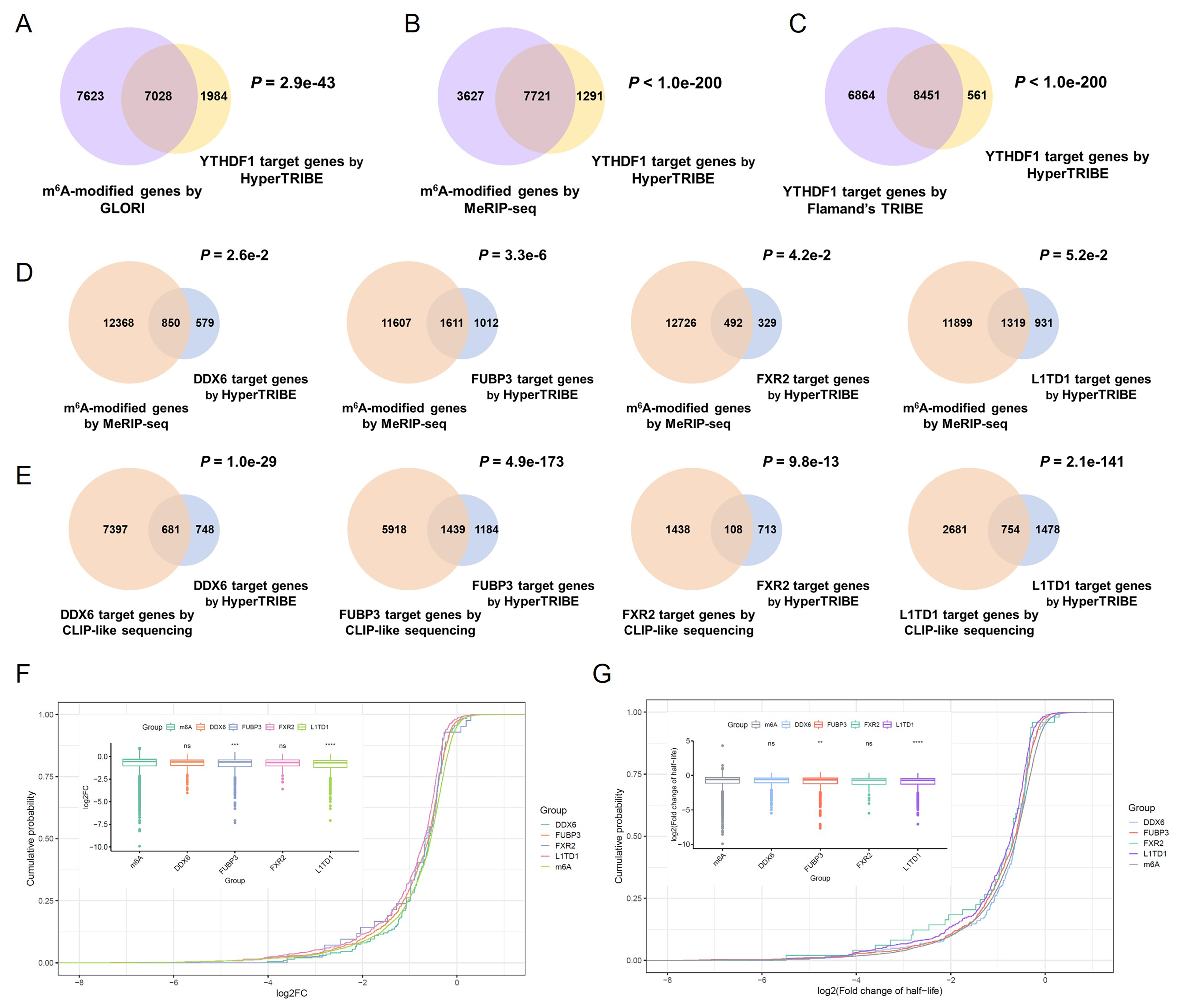


**Supplementary Figure S17.** **Overlaps between the binding target genes of putative m^6^A readers and m^6^A modification.**

HyperTRIBE assay of YTHDF1 in HEK293T was performed as the positive control assay. Then the HyperTRIBE assay was performed in the target cell type (hESCs) for the four putative m^6^A readers (DDX6, FUBP3, FXR2 and L1TD1) found in this study. (**A-B**) Venn diagram showing the intersection between YTHDF1 target genes identified by HyperTRIBE and m^6^A target genes identified by GLORI (**A**) or MeRIP-seq (**B**) in HEK293T. (**C**) Venn diagram showing the intersection between YTHDF1 target genes identified by HyperTRIBE and those identified by Flamand et al. (**D**) Venn diagrams showing the intersection between DDX6, FUBP3, FXR2 and L1TD1 target genes identified by HyperTRIBE and m^6^A target genes identified by MeRIP-seq in hESC. (**E**) Venn diagrams showing the intersection between DDX6, FUBP3, FXR2 and L1TD1 target genes identified by HyperTRIBE and those identified by CLIP-like techniques from the public data. (**F-G**) Overview of m^6^A readers-related half-life changes. The comparison was performed between m^6^A-normal cells (shControl) versus m^6^A-disrupted cells (shMETTL3) as the background. Only m^6^A-modified genes that are also targeted by specific readers are compared. The m^6^A-modified gene were derived from GLORI (**F**) or MeRIP-seq (**G**). Data in (**A-E**) and (**F-G**) were statistically analyzed using Fisher exact test and Wilcoxon test, respectively. For all box plots, boxes cover Q1 to Q3, while whiskers extend to 1.5 IQR. For all statistical plots, * means *P* < 0.05, ** means *P* < 0.01, *** means *P* < 0.001, **** means *P* < 0.0001, ns means not significant.


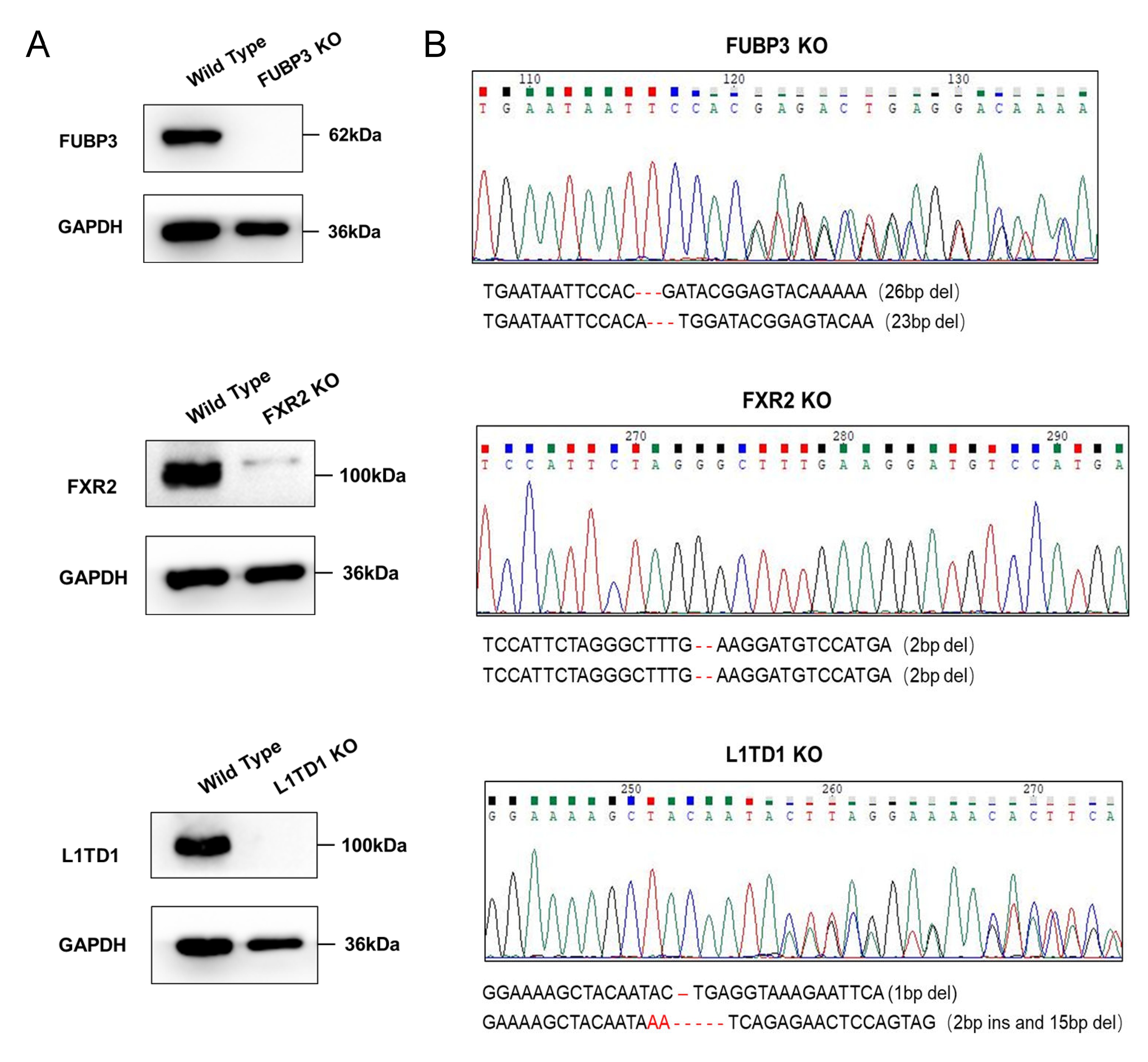


**Supplementary Figure S18. Validation of *FUBP3*, *FXR2* and *L1TD1* KO hESCs by Western blot and sanger sequencing.**

(**A**) The validation of *FUBP3*, *FXR2* and *L1TD1* KO cell lines by Western blot. (**B**) The validation of *FUBP3*, *FXR2* and *L1TD1* KO cell lines by sanger sequencing.

**
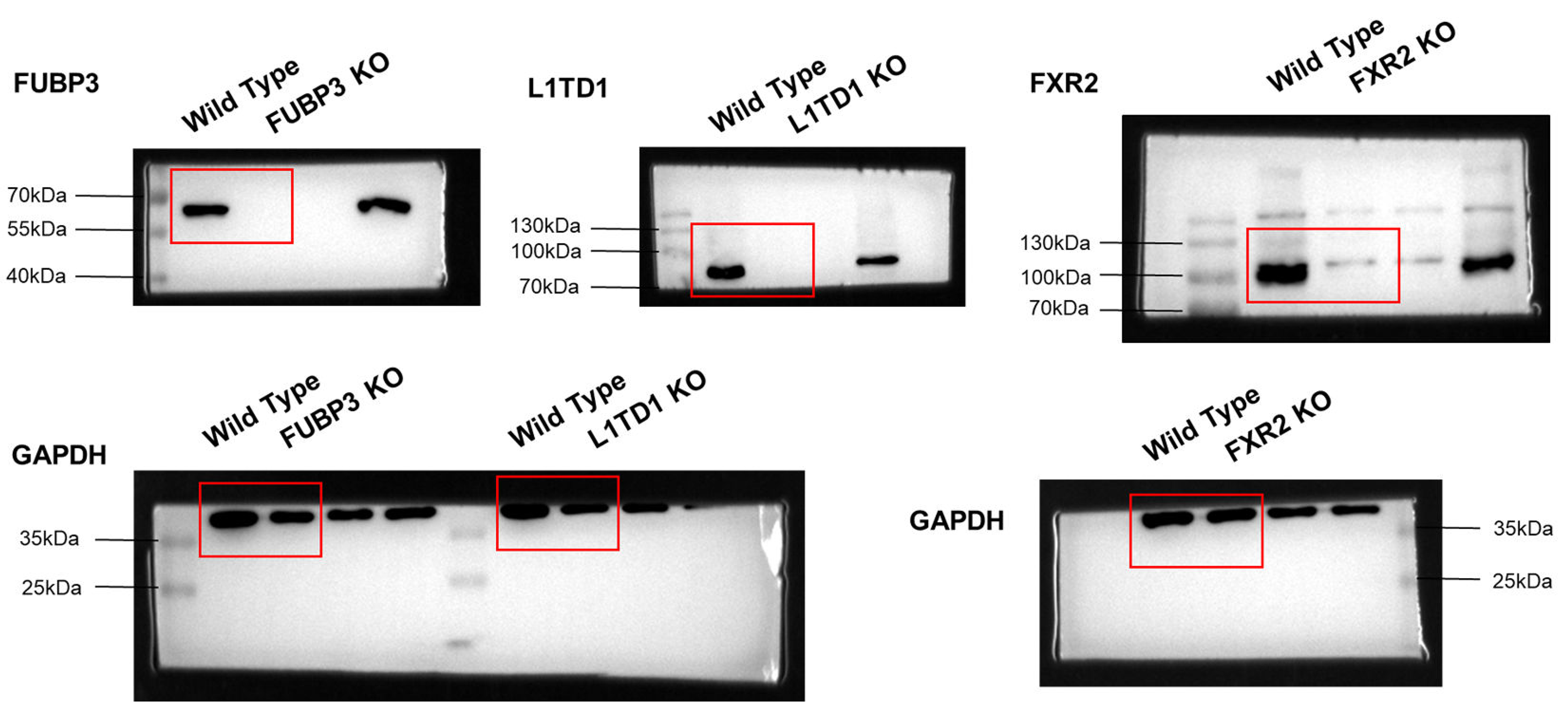
**

**Supplementary Figure S19. Source images of Western blot assay verifying *FUBP3*, *FXR2* and *L1TD1* KO hESCs.**

Source gel images of Supplementary Figure S18A are shown.

**
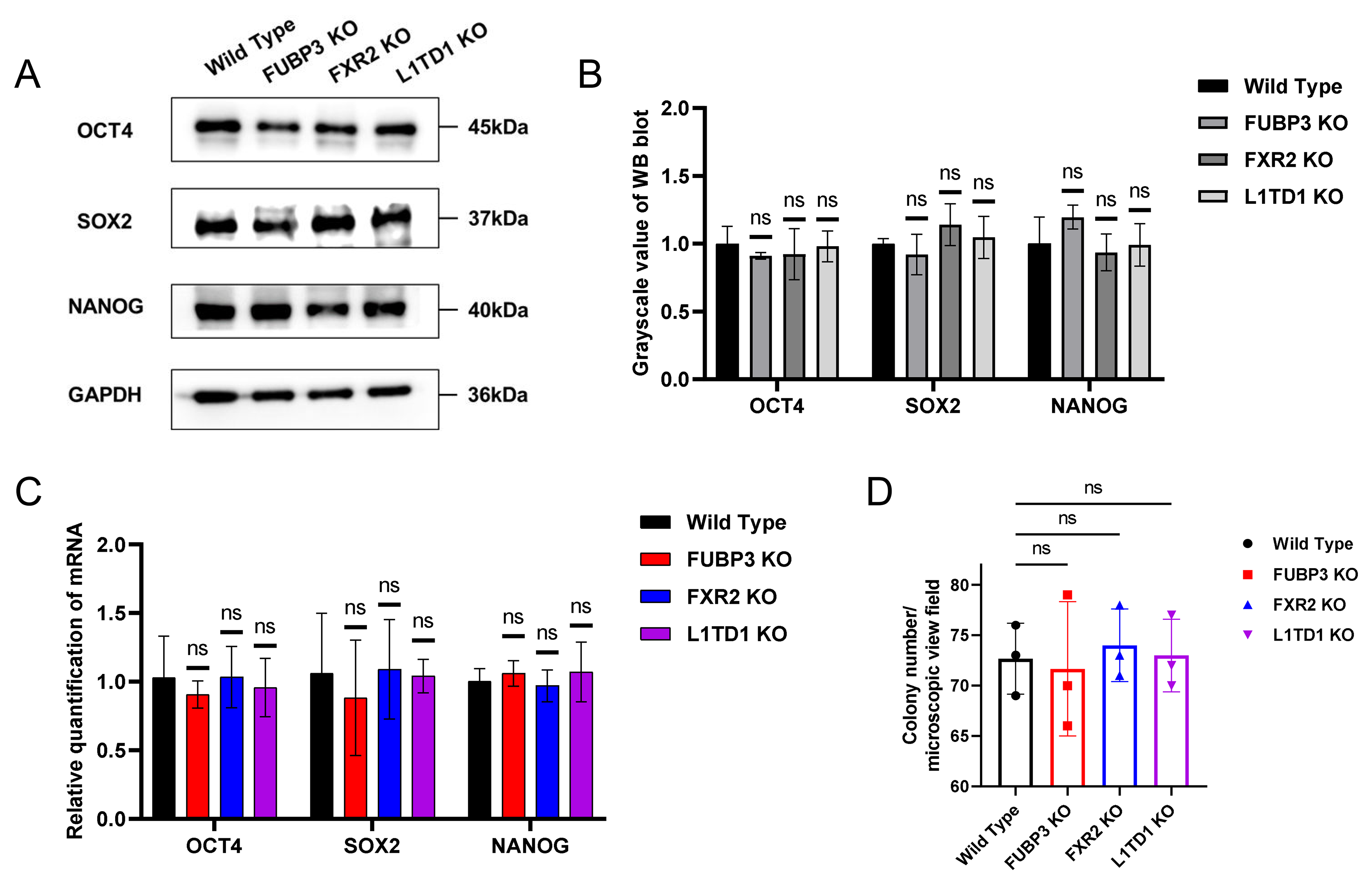
**

**Supplementary Figure S20. Newly identified m^6^A binding proteins do not affect self-renewal of hESCs.**

(**A**) Representative western blot analysis of expression of OCT4, SOX2 and NANOG in WT versus *FUBP3*, *FXR2* and *L1TD1* KO hESCs. (**B**) The statistics of grayscale value of western blot, ns means not significant. (**C**) RT-qPCR analysis of OCT4, SOX2, NANOG mRNA expression in WT versus *FUBP3*, *FXR2* and *L1TD1* KO hESCs. (**D**) Statistics of the ALP-positive colony numbers from WT versus *FUBP3*, *FXR2* and *L1TD1* KO hESCs. Data in (**B**-**D**) were statistically analyzed using two-tailed unpaired Student’s t-test. Error bars represent mean ± SD (n = 3 independent experiments). ns means not significant.

**
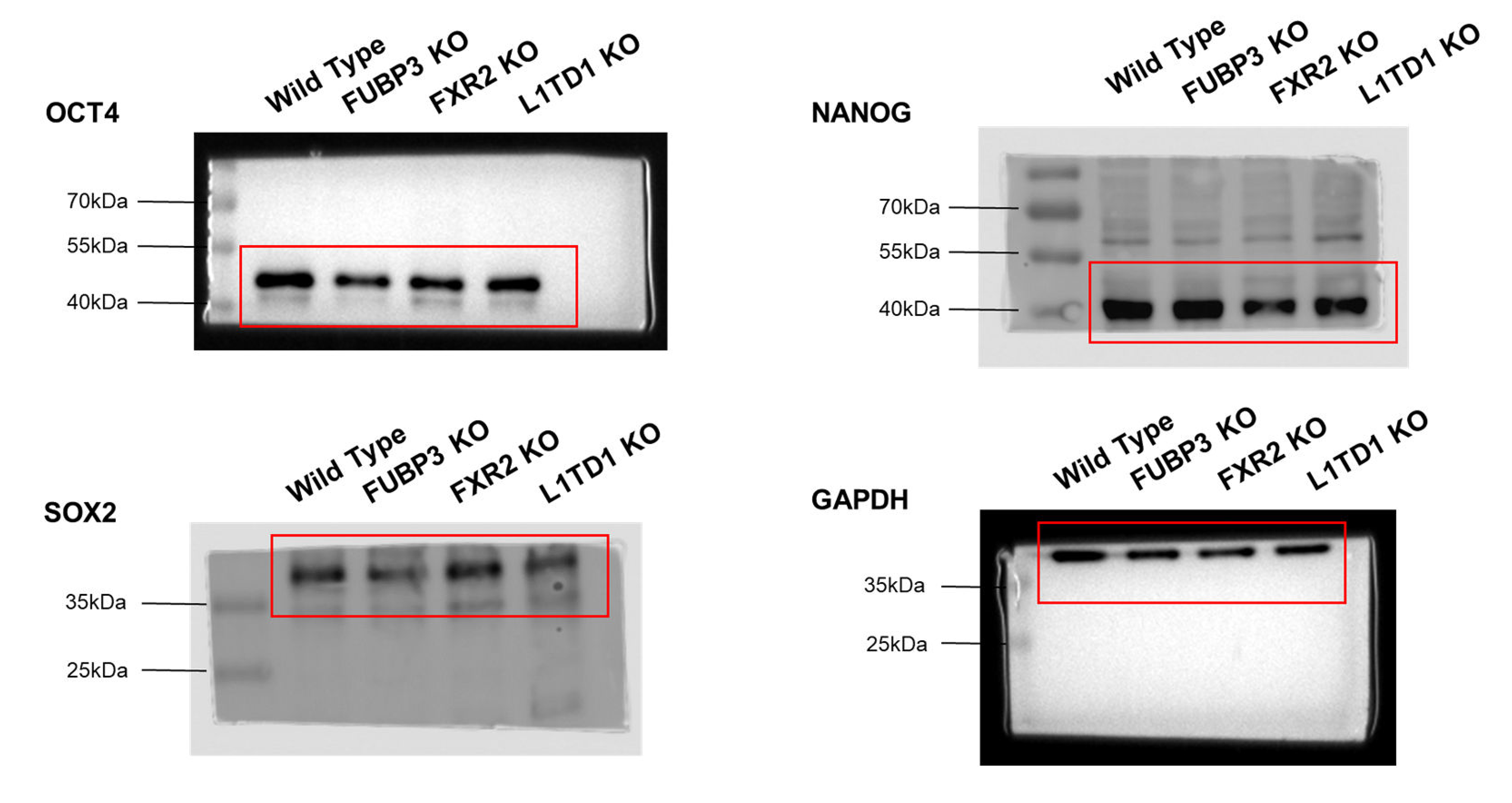
**

**Supplementary Figure S21. Source images of Western blot assay evaluating expression of OCT4, SOX2 and NANOG in WT versus *FUBP3*, *FXR2* and *L1TD1* KO hESCs.**

Source gel images of Supplementary Figure S20A are shown.


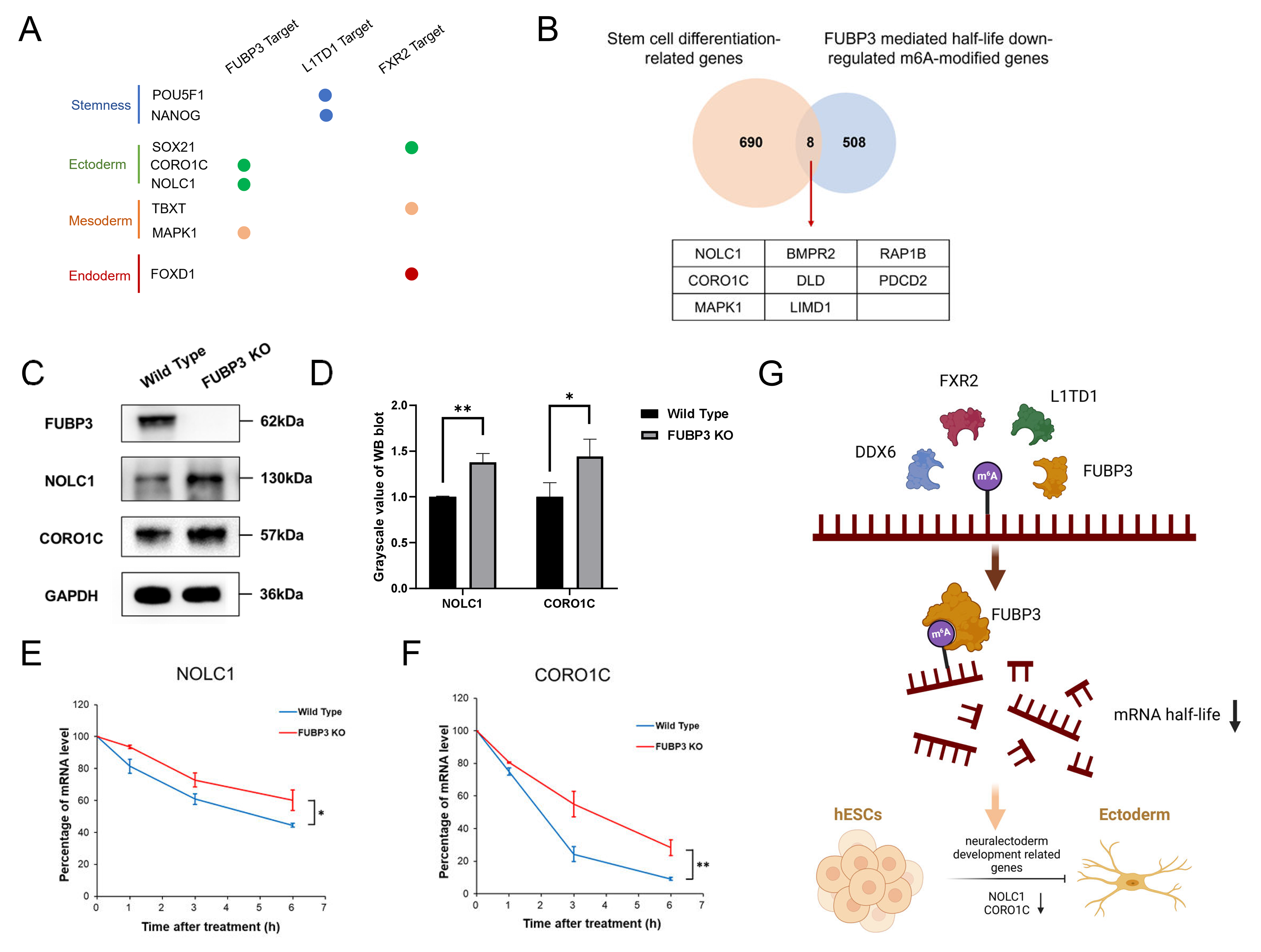


**Supplementary Figure S22.** **Possible regulatory targets of m^6^A readers for embryonic stem cell differentiation.**

(**A**) Bubble plot summarizing the distinction in target gene preference of different m^6^A binding proteins. Data were derived from HyperTRIBE assays in this study. (**B**) Venn diagram showing the intersection analysis between stem cell differentiation-related genes from GO-BP database and the FUBP3-targeted half-life down-regulated m^6^A-modified genes. (**C**) Representative western blot analysis of expression of FUBP3, NOLC1 and CORO1C in wild type and *FUBP3* KO hESCs. (**D**) The statistical histogram of grayscale value of western blot. (**E-F**) RT-qPCR analysis comparing mRNA decay rate of *NOLC1* (**E**) and *CORO1C* (**F**) between wild type and *FUBP3* KO hESCs. (**G**) Working model scheme summarizing the possible regulatory mechanism for the m^6^A readers and m^6^A binding proteins in regulating hESC differentiation. The scheme was generated with BioRender (https://BioRender.com). Data in (**C**-**F**) were statistically analyzed using two-tailed unpaired Student's t-test. Error bars represent mean ± SD (n = 3 independent experiments). * means *P* < 0.05, ** means *P* < 0.01, *** means *P* < 0.001, **** means *P* < 0.0001, ns means not significant.

**
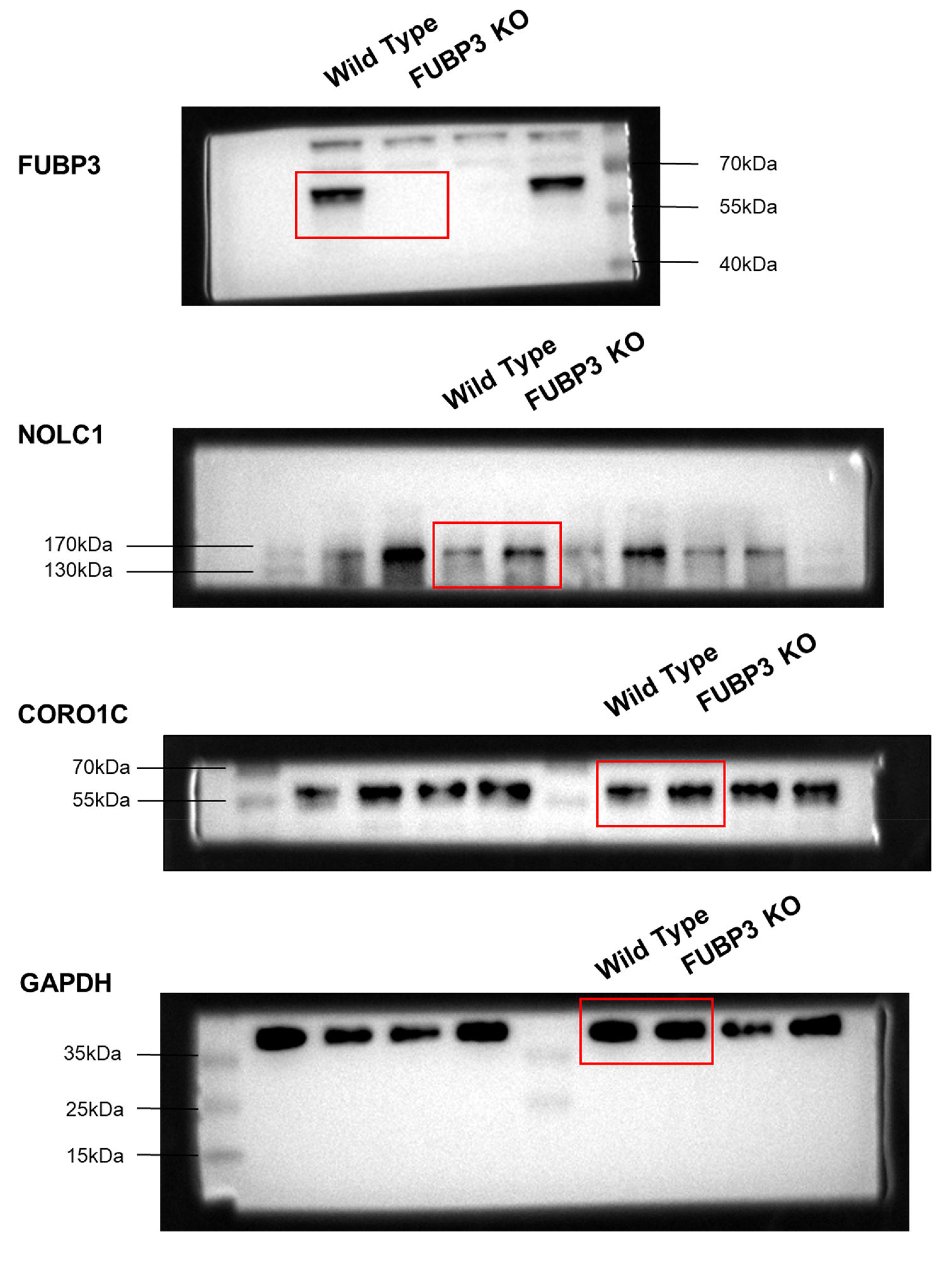
**

**Supplementary Figure S23. Source images of Western blot assay evaluating expression of FUBP3, NOLC1 and CORO1C in wild type and *FUBP3* KO hESCs.**

Source gel images of Supplementary Figure S22C are shown.

**Supplementary Table S1. Oligo Sequences Synthesized Targeting METTL3.**

| Gene | 5’ | Stem | Loop | Stem | 3’ |
| --- | --- | --- | --- | --- | --- |
| METTL3 | CTCGAGAAGGT  ATATTGCTGTTG  ACAGTGAGCG | ACCAAGGAACA  ATCCATTGTTG | TAGTGAAGCC  ACAGATGTA | CAACAATGGAT  TGTTCCTTGGC | TGCCTACTGC  CTCGCAATTG |

**Supplementary Table S2. RT-qPCR Primers for mRNA Quantification.**

| Gene | Forward | Reverse |
| --- | --- | --- |
| *GAPDH* | CTGGGCTACACTGAGCACC | AAGTGGTCGTTGAGGGCAATG |
| *METTL3* | CAAGCTGCACTTCAGACGAA | GCTTGGCGTGTGGTCTTT |
| *OCT4* | CTTGAATCCCGAATGGAAAGGG | GTGTATATCCCAGGGTGATCCTC |
| *NANOG* | ACAACTGGCCGAAGAATAGCA | GGTTCCCAGTCGGGTTCAC |
| *SOX2* | CCGTTCATCGACGAGGCTAA | ATGTGCGCGTAACTGTCCAT |
| *TBXT (T)* | TATGAGCCTCGAATCCACATAGT | CCTCGTTCTGATAAGCAGTCAC |
| *CD34* | CTACAACACCTAGTACCCTTGGA | GGTGAACACTGTGCTGATTACA |
| *SOX17* | CCTTCACGTGTACTACGGCG | GTTCAAATTCCGTGCGGTCC |
| *SOX1* | CAACCAGGACCGGGTCAAAC | CCTCGGACATGACCTTCCACT |
| *PAX6* | CGAGACTGGCTCCATCAGAC | CTTTTCGCTAGCCAGGTTGC |
| *NES* | AAGAGACTCAACAGCGACGG | TCTTGTCCCGCAGACTTCAG |
| *FOXA2* | GGAACACCACTACGCCTTCAAC | AGTGCATCACCTGTTCGTAGGC |

**Supplementary Table S3. Antibodies Used in This Study.**

| Name of Antibodies | Company | Catalog Number |
| --- | --- | --- |
| GAPDH | ABclonal | AC001 |
| METTL3 | CST | 86132S |
| OCT4 | Santa cruz | sc5279 |
| NANOG | Abcam | ab173368 |
| SOX2 | Abcam | ab171380 |
| YTHDF1 | Proteintech | 17479-1-AP |
| YTHDC2 | Proteintech | 27779-1-AP |
| DDX6 | Proteintech | 14632-1-AP |
| FUBP3 | Proteintech | 10623-1-AP |
| FXR2 | Proteintech | 12552-1-AP |
| L1TD1 | Proteintech | 21528-1-AP |
| MTDH | Proteintech | 68592-1-Ig |
| UBAP2L | Abcam | ab70319 |
| AQR | ABclonal | A6011 |
| GEMIN5 | ABclonal | A17125 |
| DDX3X | ABclonal | A5637 |
| ACTN3 | ABclonal | A12797 |
| QKI | Proteintech | 13169-1-AP |
| PTBP1 | Proteintech | 67462-1-Ig |
| EIF4A3 | ABclonal | A8985 |
| RANGAP1 | Proteintech | 67146-1-Ig |
| KHDRBS1 | Proteintech | 10222-1-AP |
| RBFOX2 | ABclonal | A20810 |
| TARDBP | Proteintech | 10782-2-AP |
| HRP-conjugated anti-rabbit IgG | Beyotime | A0208 |
| HRP-conjugated anti-mouse IgG | Beyotime | A0216 |

**Supplementary Table S4. Public MeRIP-seq data used in this study.**

| Cell line | GEO ID | Cell line | GEO ID |
| --- | --- | --- | --- |
| A549 | GSE76367 | HeLa | GSE165690 |
| HEK293T | GSE182607 | HepG2 | GSE110320 |
| hESC | GSE52600 | Huh7 | GSE155413 |
| HUVEC | GSE142386 | MOLM13 | GSE94613 |
| JURKAT | GSE154035 | H460 | GSE240879 |

**Supplementary Table S5. The RBP binding site profile used in this study.**

| **RBP** | **Source^a)^** | **Technique** | **Dataset ID^b)^** |
| --- | --- | --- | --- |
| AARS1 | ENCODE | eCLIP | - |
| AATF | ENCODE | eCLIP | - |
| ABCF1 | ENCODE | eCLIP | - |
| ACIN1 | GEO-reanalysis | classical-CLIP | GSE81460 |
| ACTN3 | GEO-processed | eCLIP | GSE221870 |
| ADAR | GEO-reanalysis | classical-CLIP | GSE227455, GSE161943 |
| ADAR | GEO-processed | eCLIP | GSE227455 |
| ADAR | ENCODE | eCLIP | - |
| ADAT1 | ENCODE | eCLIP | - |
| AGGF1 | ENCODE | eCLIP | - |
| AGO2 | ENCODE | eCLIP | - |
| AIFM1 | GEO-processed | eCLIP | GSE86035 |
| AIMP1 | GEO-processed | eCLIP | GSE117290 |
| AKAP1 | ENCODE | eCLIP | - |
| AKAP8 | GEO-processed | seCLIP | GSE139074 |
| AKAP8L | ENCODE | eCLIP | - |
| ALKBH1 | POSTAR | classical-CLIP | - |
| ALKBH3 | POSTAR, GEO-reanalysis | classical-CLIP | GSE112625 |
| ALKBH5 | POSTAR, GEO-reanalysis | classical-CLIP | GSE242283 |
| ALYREF | POSTAR, GEO-reanalysis | classical-CLIP | GSE242283, GSE113896, GSE115429, GSE133620 |
| APEX1 | ENCODE | eCLIP | - |
| APOBEC3C | ENCODE | eCLIP | - |
| AQR | ENCODE | eCLIP | - |
| ATXN2 | POSTAR | classical-CLIP | - |
| BCCIP | ENCODE | eCLIP | - |
| BCLAF1 | ENCODE | eCLIP | - |
| BOLL | GEO-processed | eCLIP | GSE117290 |
| BUD13 | ENCODE | eCLIP | - |
| CACTIN | ENCODE | eCLIP | - |
| CAPRIN1 | POSTAR | classical-CLIP | - |
| CDC40 | ENCODE | eCLIP | - |
| CDK1 | POSTAR, GEO-reanalysis | classical-CLIP | GSE100754 |
| CELF1 | GEO-processed | PIE-SEQ | GSE155844 |
| CELF2 | POSTAR | classical-CLIP | - |
| CELF2 | GEO-processed | PIE-SEQ | GSE155844 |
| CELF4 | GEO-processed | PIE-SEQ | GSE155844 |
| CHD7 | GEO-reanalysis | classical-CLIP | GSE155844, GSE171137 |
| CHTOP | GEO-reanalysis | classical-CLIP | GSE155844, GSE171137, GSE113896 |
| CLK3 | GEO-processed | eCLIP | GSE117290 |
| CNBP | POSTAR | classical-CLIP | - |
| CNOT7 | GEO-processed | eCLIP | GSE117290 |
| CPEB4 | GEO-processed | irCLIP | GSE188693 |
| CPEB4 | ENCODE | eCLIP | - |
| CPSF1 | POSTAR | classical-CLIP | - |
| CPSF2 | POSTAR | classical-CLIP | - |
| CPSF3 | POSTAR | classical-CLIP | - |
| CPSF4 | POSTAR | classical-CLIP | - |
| CPSF6 | POSTAR | classical-CLIP | - |
| CPSF6 | ENCODE | eCLIP | - |
| CPSF7 | POSTAR | classical-CLIP | - |
| CSDE1 | POSTAR, GEO-reanalysis | classical-CLIP | EMTAB3818 |
| CSTF2 | POSTAR | classical-CLIP | - |
| CSTF2 | GEO-processed | sCLIP | GSE92995 |
| CSTF2 | ENCODE | eCLIP | - |
| CSTF2T | POSTAR | classical-CLIP | - |
| CSTF2T | ENCODE | eCLIP | - |
| CTCF | POSTAR, GEO-reanalysis | classical-CLIP | GSE53554 |
| DCP1B | GEO-processed | eCLIP | GSE112782 |
| DDX1 | ENCODE | eCLIP | - |
| DDX21 | ENCODE | eCLIP | - |
| DDX24 | ENCODE | eCLIP | - |
| DDX3X | POSTAR | classical-CLIP | - |
| DDX3X | GEO-processed | eCLIP | GSE205536 |
| DDX3X | ENCODE | eCLIP | - |
| DDX42 | ENCODE | eCLIP | - |
| DDX43 | ENCODE | eCLIP | - |
| DDX47 | ENCODE | eCLIP | - |
| DDX5 | GEO-processed | eCLIP | GSE115442 |
| DDX51 | ENCODE | eCLIP | - |
| DDX52 | ENCODE | eCLIP | - |
| DDX54 | POSTAR, GEO-reanalysis | classical-CLIP | GSE89366 |
| DDX55 | ENCODE | eCLIP | - |
| DDX59 | ENCODE | eCLIP | - |
| DDX6 | GEO-processed | eCLIP | GSE117290 |
| DDX6 | ENCODE | eCLIP | - |
| DEK | ENCODE | eCLIP | - |
| DGCR8 | POSTAR | classical-CLIP | - |
| DGCR8 | ENCODE | eCLIP | - |
| DHX30 | ENCODE | eCLIP | - |
| DHX34 | GEO-processed | seCLIP | GSE204982 |
| DHX38 | ENCODE | eCLIP | - |
| DICER1 | POSTAR | classical-CLIP | - |
| DIS3 | POSTAR, GEO-reanalysis | classical-CLIP | GSE64332 |
| DIS3L2 | POSTAR | classical-CLIP | - |
| DKC1 | ENCODE | eCLIP | - |
| DMD | POSTAR, GEO-reanalysis | classical-CLIP | GSE128318 |
| DROSHA | ENCODE | eCLIP | - |
| EED | ENCODE | eCLIP | - |
| EEF2 | ENCODE | eCLIP | - |
| EFTUD2 | ENCODE | eCLIP | - |
| EIF2B5 | GEO-processed | eCLIP | GSE213867 |
| EIF2D | GEO-processed | eCLIP | GSE213867 |
| EIF2S2 | GEO-processed | eCLIP | GSE213867 |
| EIF3A | POSTAR | classical-CLIP | - |
| EIF3A | ENCODE | eCLIP | - |
| EIF3B | POSTAR | classical-CLIP | - |
| EIF3D | POSTAR | classical-CLIP | - |
| EIF3D | ENCODE | eCLIP | - |
| EIF3G | POSTAR | classical-CLIP | - |
| EIF3G | GEO-processed | eCLIP | GSE205536 |
| EIF3G | ENCODE | eCLIP | - |
| EIF3H | ENCODE | eCLIP | - |
| EIF3J | GEO-processed | eCLIP | GSE213867 |
| EIF4A3 | POSTAR, GEO-reanalysis | classical-CLIP | EMTAB4000 |
| EIF4E | POSTAR, GEO-reanalysis | classical-CLIP | EMTAB4000, GSE112276 |
| EIF4E | ENCODE | eCLIP | - |
| EIF4G2 | ENCODE | eCLIP | - |
| ELAC2 | ENCODE | eCLIP | - |
| ELAVL1 | POSTAR, GEO-reanalysis | classical-CLIP | EMTAB11854, GSE133620, GSE161943 |
| ELAVL1 | ENCODE | eCLIP | - |
| ESRP1 | POSTAR, GEO-reanalysis | classical-CLIP | EMTAB11854, GSE133620, GSE161943, GSE233931 |
| ESRRA | POSTAR, GEO-reanalysis | classical-CLIP | EMTAB11854, GSE133620, GSE161943, GSE233931, GSE173628 |
| EWSR1 | POSTAR | classical-CLIP | - |
| EWSR1 | ENCODE | eCLIP | - |
| EXOSC10 | POSTAR, GEO-reanalysis | classical-CLIP | GSE120574 |
| EXOSC10 | ENCODE | eCLIP | - |
| EXOSC5 | ENCODE | eCLIP | - |
| EZH2 | POSTAR | classical-CLIP | - |
| FAM120A | GEO-processed | eCLIP | GSE205536 |
| FAM120A | ENCODE | eCLIP | - |
| FASTKD2 | ENCODE | eCLIP | - |
| FBL | POSTAR | classical-CLIP | - |
| FIP1L1 | POSTAR | classical-CLIP | - |
| FKBP4 | ENCODE | eCLIP | - |
| FMR1 | POSTAR | classical-CLIP | - |
|  |  |  |  |
| FMR1 | GEO-processed | PIE-SEQ | GSE155844 |
| FMR1 | ENCODE | eCLIP | - |
| FTO | ENCODE | eCLIP | - |
| FUBP1 | GEO-reanalysis | classical-CLIP | GSE155844, GSE115429 |
| FUBP1 | GEO-processed | PIE-SEQ | GSE155844 |
| FUBP3 | POSTAR, GEO-reanalysis | classical-CLIP | GSE115429 |
| FUBP3 | ENCODE | eCLIP | - |
| FUS | POSTAR | classical-CLIP | - |
| FUS | GEO-processed | eCLIP | GSE112782 |
| FUS | ENCODE | eCLIP | - |
| FXR1 | POSTAR | classical-CLIP | - |
| FXR1 | ENCODE | eCLIP | - |
| FXR2 | POSTAR | classical-CLIP | - |
| FXR2 | ENCODE | eCLIP | - |
| G3BP1 | POSTAR, GEO-reanalysis | classical-CLIP | GSE98856 |
| G3BP1 | ENCODE | eCLIP | - |
| G3BP2 | POSTAR, GEO-reanalysis | classical-CLIP | GSE98856, GSE227855, GSE98856 |
| GARS1 | ENCODE | eCLIP | - |
| GATA2 | GEO-processed | seCLIP | GSE232179 |
| GEMIN5 | POSTAR, GEO-reanalysis | classical-CLIP | GSE206967 |
| GEMIN5 | ENCODE | eCLIP | - |
| GINS1 | POSTAR, GEO-reanalysis | classical-CLIP | GSE206967, GSE90650 |
| GNL3 | ENCODE | eCLIP | - |
| GPKOW | ENCODE | eCLIP | - |
| GRSF1 | ENCODE | eCLIP | - |
| GRWD1 | ENCODE | eCLIP | - |
| GTF2F1 | ENCODE | eCLIP | - |
| HDAC9 | POSTAR, GEO-reanalysis | classical-CLIP | GSE206967, GSE90650, GSE93744 |
| HDLBP | POSTAR, GEO-reanalysis | classical-CLIP | GSE206967, GSE90650, GSE93744, GSE148259 |
| HLTF | ENCODE | eCLIP | - |
| HMGB1 | GEO-processed | sCLIP | GSE146047 |
| HMGB2 | GEO-processed | sCLIP | GSE146047 |
| HNRNPA1 | POSTAR, GEO-reanalysis | classical-CLIP | EMTAB3612, GSE83923 |
| HNRNPA1 | GEO-processed | eCLIP | GSE115442 |
| HNRNPA1 | ENCODE | eCLIP | - |
| HNRNPA2B1 | POSTAR, GEO-reanalysis | classical-CLIP | GSE35799, GSE70061 |
| HNRNPC | POSTAR | classical-CLIP | - |
| HNRNPC | ENCODE | eCLIP | - |
| HNRNPD | POSTAR | classical-CLIP | - |
| HNRNPF | POSTAR | classical-CLIP | - |
| HNRNPH1 | POSTAR, GEO-reanalysis | classical-CLIP | EMTAB6220, EMTAB6221 |
| HNRNPK | ENCODE | eCLIP | - |
| HNRNPL | POSTAR, GEO-reanalysis | classical-CLIP | EMTAB6220, EMTAB6221, GSE37560 |
| HNRNPL | ENCODE | eCLIP | - |
| HNRNPM | POSTAR, GEO-reanalysis | classical-CLIP | GSE178223, GSE85851 |
| HNRNPM | ENCODE | eCLIP | - |
| HNRNPU | POSTAR | classical-CLIP | - |
| HNRNPU | ENCODE | eCLIP | - |
| HNRNPUL1 | ENCODE | eCLIP | - |
| HSP90AA1 | GEO-processed | eCLIP | GSE221870 |
| IFIT2 | GEO-processed | eCLIP | GSE117290 |
| IGF2BP1 | POSTAR | classical-CLIP | - |
| IGF2BP1 | GEO-processed | PIE-SEQ | GSE155844 |
| IGF2BP1 | ENCODE | eCLIP | - |
| IGF2BP2 | POSTAR, GEO-reanalysis | classical-CLIP | GSE73847 |
| IGF2BP2 | GEO-processed | eCLIP | GSE205536 |
| IGF2BP2 | GEO-processed | eCLIP | GSE192792 |
| IGF2BP2 | GEO-processed | PIE-SEQ | GSE155844 |
| IGF2BP2 | ENCODE | eCLIP | - |
| IGF2BP3 | POSTAR | classical-CLIP | - |
| IGF2BP3 | ENCODE | eCLIP | - |
| ILF2 | GEO-processed | eCLIP | GSE112782 |
| ILF3 | ENCODE | eCLIP | - |
| IMP3 | GEO-reanalysis | classical-CLIP | GSE112782, SRP139915 |
| INO80B | GEO-processed | eCLIP | GSE221870 |
| KHDRBS1 | GEO-processed | PIE-SEQ | GSE155844 |
| KHDRBS1 | ENCODE | eCLIP | - |
| KHDRBS2 | GEO-processed | PIE-SEQ | GSE155844 |
| KHDRBS3 | GEO-processed | PIE-SEQ | GSE155844 |
| KHSRP | POSTAR, GEO-reanalysis | classical-CLIP | GSE161943 |
| KHSRP | ENCODE | eCLIP | - |
| L1TD1 | POSTAR, GEO-reanalysis | classical-CLIP | GSE161943, GSE227855 |
| LARP1 | POSTAR, GEO-reanalysis | classical-CLIP | GSE161943, GSE227855, GSE59599 |
| LARP4 | ENCODE | eCLIP | - |
| LARP4B | POSTAR, GEO-reanalysis | classical-CLIP | GSE161943, GSE227855, GSE59599, GSE69169 |
| LARP6 | POSTAR, GEO-reanalysis | classical-CLIP | GSE161943, GSE227855, GSE59599, GSE69169, EMTAB9636 |
| LARP7 | ENCODE | eCLIP | - |
| LIN28A | POSTAR | classical-CLIP | - |
| LIN28A | GEO-processed | PIE-SEQ | GSE155844 |
| LIN28B | POSTAR | classical-CLIP | - |
| LIN28B | GEO-processed | eCLIP | GSE205536 |
| LIN28B | GEO-processed | PIE-SEQ | GSE155844 |
| LIN28B | ENCODE | eCLIP | - |
| LSM11 | ENCODE | eCLIP | - |
| MATR3 | ENCODE | eCLIP | - |
| MBNL1 | POSTAR, GEO-reanalysis | classical-CLIP | GSE76486 |
| MBNL1 | ENCODE | eCLIP | - |
| MBNL2 | POSTAR | classical-CLIP | - |
| MCCC1 | GEO-processed | eCLIP | GSE221870 |
| METAP2 | ENCODE | eCLIP | - |
| METTL1 | POSTAR, GEO-reanalysis | classical-CLIP | GSE100754, GSE112276 |
| METTL1 | ENCODE | eCLIP | - |
| METTL14 | POSTAR | classical-CLIP | - |
| METTL3 | POSTAR | classical-CLIP | - |
| MEX3C | GEO-processed | eCLIP | GSE117290 |
| MFAP1 | ENCODE | eCLIP | - |
| MKRN1 | GEO-reanalysis | classical-CLIP | GSE117290, GSE122869 |
| MORC2 | ENCODE | eCLIP | - |
| MOV10 | POSTAR | classical-CLIP | - |
| MSI1 | POSTAR, GEO-reanalysis | classical-CLIP | GSE68800 |
| MSI2 | POSTAR, GEO-reanalysis | classical-CLIP | GSE68800, GSE69583 |
| MTDH | GEO-reanalysis | classical-CLIP | GSE117290, GSE110260 |
| MTDH | GEO-processed | eCLIP | GSE117290 |
| MTPAP | ENCODE | eCLIP | - |
| NANOS3 | GEO-processed | eCLIP | GSE117290 |
| NCBP2 | ENCODE | eCLIP | - |
| NCBP3 | POSTAR | classical-CLIP | - |
| NIP7 | ENCODE | eCLIP | - |
| NIPBL | ENCODE | eCLIP | - |
| NKRF | ENCODE | eCLIP | - |
| NOL12 | ENCODE | eCLIP | - |
| NOLC1 | ENCODE | eCLIP | - |
| NONO | POSTAR, GEO-reanalysis | classical-CLIP | GSE114376, GSE90650 |
| NONO | ENCODE | eCLIP | - |
| NOP56 | POSTAR | classical-CLIP | - |
| NOP58 | POSTAR | classical-CLIP | - |
| NOVA1 | GEO-processed | PIE-SEQ | GSE155844 |
| NPM1 | ENCODE | eCLIP | - |
| NR5A1 | GEO-processed | eCLIP | GSE221870 |
| NSUN2 | ENCODE | eCLIP | - |
| NSUN4 | ENCODE | eCLIP | - |
| NUDT16L1 | GEO-reanalysis | classical-CLIP | GSE221870, GSE118049 |
| NUDT21 | POSTAR, GEO-reanalysis | classical-CLIP | GSE161943 |
| NUDT21 | GEO-processed | eCLIP | GSE190842 |
| NUMA1 | GEO-processed | eCLIP | GSE86035 |
| NXF1 | GEO-reanalysis | classical-CLIP | GSE86035, GSE113896 |
| OBI1 | GEO-processed | eCLIP | GSE86035 |
| PABPC4 | ENCODE | eCLIP | - |
| PABPN1 | ENCODE | eCLIP | - |
| PAIP1 | GEO-processed | RIP | GSE150780 |
| PARN | GEO-processed | eCLIP | GSE117290 |
| PARP1 | GEO-reanalysis | classical-CLIP | GSE117290, GSE95360 |
| PARP1 | ENCODE | eCLIP | - |
| PBRM1 | GEO-reanalysis | classical-CLIP | GSE117290, GSE95360, GSE221619 |
| PCBP1 | GEO-reanalysis | classical-CLIP | GSE117290, GSE95360, GSE221619, GSE84700 |
| PCBP1 | ENCODE | eCLIP | - |
| PCBP2 | ENCODE | eCLIP | - |
| PHC1 | GEO-reanalysis | classical-CLIP | GSE117290, GSE95360, GSE221619, GSE84700, GSE103233 |
| PHF6 | ENCODE | eCLIP | - |
| PIAS4 | GEO-processed | eCLIP | GSE221870 |
| POLR2G | ENCODE | eCLIP | - |
| PPIG | ENCODE | eCLIP | - |
| PPIL4 | ENCODE | eCLIP | - |
| PRDX1 | GEO-processed | RIP | GSE134504 |
| PRKRA | POSTAR | classical-CLIP | - |
| PRPF39 | ENCODE | eCLIP | - |
| PRPF4 | ENCODE | eCLIP | - |
| PRPF8 | GEO-processed | eCLIP | GSE205536 |
| PRPF8 | ENCODE | eCLIP | - |
| PRRC2B | GEO-reanalysis | classical-CLIP | GSE205536, GSE220057 |
| PTBP1 | POSTAR, GEO-reanalysis | classical-CLIP | EMTAB5027 |
| PTBP1 | GEO-processed | eCLIP | GSE192792 |
| PTBP1 | ENCODE | eCLIP | - |
| PTBP3 | POSTAR, GEO-reanalysis | classical-CLIP | EMTAB5027, GSE161943 |
| PUM1 | POSTAR, GEO-reanalysis | classical-CLIP | EMTAB5027, GSE161943, GSE110519, GSE115429 |
| PUM1 | ENCODE | eCLIP | - |
| PUM2 | POSTAR, GEO-reanalysis | classical-CLIP | GSE110519 |
| PUM2 | GEO-processed | eCLIP | GSE205536 |
| PUM2 | GEO-processed | PIE-SEQ | GSE155844 |
| PUM2 | ENCODE | eCLIP | - |
| PUS1 | ENCODE | eCLIP | - |
| QKI | POSTAR, GEO-reanalysis | classical-CLIP | GSE161943 |
| QKI | GEO-processed | PIE-SEQ | GSE155844 |
| QKI | GEO-processed | PIE-SEQ | GSE155844 |
| QKI | ENCODE | eCLIP | - |
| RANGAP1 | GEO-processed | eCLIP | GSE86035 |
| RBFOX2 | POSTAR, GEO-reanalysis | classical-CLIP | GSE229012, GSE85851 |
| RBFOX2 | GEO-processed | eCLIP | GSE205536 |
| RBFOX2 | ENCODE | eCLIP | - |
| RBM10 | POSTAR, GEO-reanalysis | classical-CLIP | GSE48066 |
| RBM12 | GEO-processed | eCLIP | GSE200676 |
| RBM15 | POSTAR | classical-CLIP | - |
| RBM15 | ENCODE | eCLIP | - |
| RBM15B | POSTAR | classical-CLIP | - |
| RBM20 | GEO-processed | eCLIP | GSE176060 |
| RBM22 | ENCODE | eCLIP | - |
| RBM3 | GEO-reanalysis | classical-CLIP | GSE176060, GSE242283, GSE241548 |
| RBM33 | GEO-reanalysis | classical-CLIP | GSE176060, GSE242283 |
| RBM4 | POSTAR, GEO-reanalysis | classical-CLIP | GSE147895 |
| RBM45 | GEO-reanalysis | classical-CLIP | GSE176060, GSE242283, GSE241548, GSE205625 |
| RBM47 | POSTAR | classical-CLIP | - |
| RBM5 | POSTAR, GEO-reanalysis | classical-CLIP | GSE48066 |
| RBM5 | ENCODE | eCLIP | - |
| RBM6 | POSTAR, GEO-reanalysis | classical-CLIP | GSE48066, GSE48066 |
| RBMX | POSTAR, GEO-reanalysis | classical-CLIP | GSE48066, GSE48066, GSE114311, GSE115429, GSE74085 |
| RBPMS | POSTAR | classical-CLIP | - |
| RC3H1 | POSTAR, GEO-reanalysis | classical-CLIP | GSE69153 |
| RELA | POSTAR, GEO-reanalysis | classical-CLIP | GSE69153, GSE197705 |
| RNF187 | ENCODE | eCLIP | - |
| RNMT | POSTAR, GEO-reanalysis | classical-CLIP | GSE69153, GSE197705, GSE112276 |
| RO60 | ENCODE | eCLIP | - |
| RPL29 | GEO-processed | eCLIP | GSE213867 |
| RPL35A | GEO-processed | eCLIP | GSE213867 |
| RPS10 | ENCODE | eCLIP | - |
| RPS11 | ENCODE | eCLIP | - |
| RPS14 | GEO-processed | eCLIP | GSE213867 |
| RPS19 | GEO-processed | eCLIP | GSE213867 |
| RPS3 | ENCODE | eCLIP | - |
| RPS3A | GEO-processed | eCLIP | GSE213867 |
| RPS6 | ENCODE | eCLIP | - |
| RTCA | GEO-processed | eCLIP | GSE232597 |
| RTCB | POSTAR | classical-CLIP | - |
| RYBP | ENCODE | eCLIP | - |
| SAFB | ENCODE | eCLIP | - |
| SAFB2 | ENCODE | eCLIP | - |
| SBDS | ENCODE | eCLIP | - |
| SBNO2 | POSTAR, GEO-reanalysis | classical-CLIP | GSE187784 |
| SCAF8 | GEO-processed | eCLIP | GSE232597 |
| SDAD1 | ENCODE | eCLIP | - |
| SERBP1 | POSTAR, GEO-reanalysis | classical-CLIP | EMTAB10830 |
| SERBP1 | ENCODE | eCLIP | - |
| SETD1A | POSTAR, GEO-reanalysis | classical-CLIP | EMTAB10830, GSE161943 |
| SF3A1 | ENCODE | eCLIP | - |
| SF3A3 | ENCODE | eCLIP | - |
| SF3B1 | POSTAR, GEO-reanalysis | classical-CLIP | GSE160948 |
| SF3B1 | ENCODE | eCLIP | - |
| SF3B4 | GEO-processed | eCLIP | GSE205536 |
| SF3B4 | ENCODE | eCLIP | - |
| SFPQ | ENCODE | eCLIP | - |
| SLBP | ENCODE | eCLIP | - |
| SLTM | ENCODE | eCLIP | - |
| SMARCA4 | GEO-reanalysis | classical-CLIP | GSE205536, GSE93744 |
| SMN1 | GEO-reanalysis | classical-CLIP | GSE205536, GSE93744, GSE110411 |
| SMNDC1 | ENCODE | eCLIP | - |
| SND1 | ENCODE | eCLIP | - |
| SNIP1 | GEO-reanalysis | classical-CLIP | GSE205536, GSE93744, GSE110411, GSE181777 |
| SNRNP200 | ENCODE | eCLIP | - |
| SNRPA | GEO-processed | eCLIP | GSE117290 |
| SNRPA1 | ENCODE | eCLIP | - |
| SNRPC | ENCODE | eCLIP | - |
| SNRPD3 | ENCODE | eCLIP | - |
| SORBS2 | GEO-processed | RIP | GSE100323 |
| SRRM4 | POSTAR | classical-CLIP | - |
| SRSF1 | POSTAR, GEO-reanalysis | classical-CLIP | GSE130867, GSE131745, GSE71096, GSE83923 |
| SRSF1 | GEO-processed | PIE-SEQ | GSE155844 |
| SRSF1 | ENCODE | eCLIP | - |
| SRSF10 | POSTAR, GEO-reanalysis | classical-CLIP | GSE71096 |
| SRSF2 | GEO-reanalysis | classical-CLIP | GSE155844, GSE111900, GSE207643 |
| SRSF2 | GEO-processed | PIE-SEQ | GSE155844 |
| SRSF3 | GEO-reanalysis | classical-CLIP | GSE155844, GSE71096 |
| SRSF3 | GEO-processed | PIE-SEQ | GSE155844 |
| SRSF5 | ENCODE | eCLIP | - |
| SRSF7 | POSTAR, GEO-reanalysis | classical-CLIP | GSE71096 |
| SRSF7 | ENCODE | eCLIP | - |
| SRSF9 | POSTAR, GEO-reanalysis | classical-CLIP | GSE71096 |
| SRSF9 | ENCODE | eCLIP | - |
| SSB | POSTAR | classical-CLIP | - |
| SSB | ENCODE | eCLIP | - |
| STAU1 | POSTAR | classical-CLIP | - |
| STAU2 | GEO-processed | eCLIP | GSE232597 |
| STAU2 | GEO-processed | PIE-SEQ | GSE155844 |
| STAU2 | ENCODE | eCLIP | - |
| SUB1 | ENCODE | eCLIP | - |
| SUGP2 | ENCODE | eCLIP | - |
| SUPV3L1 | ENCODE | eCLIP | - |
| TAF15 | POSTAR | classical-CLIP | - |
| TAF15 | ENCODE | eCLIP | - |
| TARBP2 | POSTAR | classical-CLIP | - |
| TARDBP | POSTAR, GEO-reanalysis | classical-CLIP | EMTAB4733, GSE85851 |
| TARDBP | GEO-processed | PIE-SEQ | GSE155844 |
| TARDBP | ENCODE | eCLIP | - |
| TBRG4 | POSTAR, GEO-reanalysis | classical-CLIP | GSE156556 |
| TBRG4 | ENCODE | eCLIP | - |
| TENT4B | POSTAR, GEO-reanalysis | classical-CLIP | GSE156556, GSE36987 |
| TIA1 | POSTAR | classical-CLIP | - |
| TIA1 | GEO-processed | eCLIP | GSE166402 |
| TIA1 | ENCODE | eCLIP | - |
| TIAL1 | POSTAR | classical-CLIP | - |
| TIAL1 | ENCODE | eCLIP | - |
| TNRC6A | POSTAR | classical-CLIP | - |
| TNRC6B | POSTAR | classical-CLIP | - |
| TNRC6C | POSTAR | classical-CLIP | - |
| TOB1 | GEO-processed | eCLIP | GSE117290 |
| TOB2 | GEO-processed | eCLIP | GSE117290 |
| TRA2A | ENCODE | eCLIP | - |
| TRIM25 | GEO-reanalysis | classical-CLIP | GSE117290, GSE104949 |
| TRMT10A | GEO-reanalysis | classical-CLIP | GSE117290, GSE104949, GSE146207 |
| TRNAU1AP | GEO-processed | eCLIP | GSE232597 |
| TRUB1 | GEO-reanalysis | classical-CLIP | GSE232597, GSE143510 |
| TXN | GEO-processed | RIP | GSE212595 |
| U2AF1 | POSTAR, GEO-reanalysis | classical-CLIP | GSE203531 |
| U2AF1 | ENCODE | eCLIP | - |
| U2AF2 | POSTAR, GEO-reanalysis | classical-CLIP | GSE83923, GSE99688 |
| U2AF2 | ENCODE | eCLIP | - |
| UBAP2L | GEO-processed | eCLIP | GSE117290 |
| UCHL5 | ENCODE | eCLIP | - |
| UPF1 | POSTAR | classical-CLIP | - |
| UPF1 | ENCODE | eCLIP | - |
| UTP18 | ENCODE | eCLIP | - |
| UTP3 | ENCODE | eCLIP | - |
| VIM | GEO-processed | eCLIP | GSE86035 |
| WDR3 | ENCODE | eCLIP | - |
| WDR33 | POSTAR | classical-CLIP | - |
| WDR4 | POSTAR, GEO-reanalysis | classical-CLIP | GSE112276 |
| WDR43 | ENCODE | eCLIP | - |
| WRN | ENCODE | eCLIP | - |
| WTAP | POSTAR | classical-CLIP | - |
| XPO5 | POSTAR, GEO-reanalysis | classical-CLIP | GSE111964 |
| XPO5 | ENCODE | eCLIP | - |
| XRCC6 | ENCODE | eCLIP | - |
| XRN2 | ENCODE | eCLIP | - |
| YBX1 | POSTAR, GEO-reanalysis | classical-CLIP | GSE111964, GSE133620, GSE150925, GSE249896 |
| YBX3 | ENCODE | eCLIP | - |
| YTHDC1 | POSTAR, GEO-reanalysis | classical-CLIP | GSE71096, GSE74397 |
| YTHDC1 | GEO-processed | PIE-SEQ | GSE155844 |
| YTHDC2 | POSTAR | classical-CLIP | - |
| YTHDC2 | GEO-processed | PIE-SEQ | GSE155844 |
| YTHDF1 | POSTAR | classical-CLIP | - |
| YTHDF1 | GEO-processed | STAMP | GSE200712 |
| YTHDF1 | GEO-processed | TRIBE | GSE200712 |
| YTHDF1 | GEO-processed | PIE-SEQ | GSE155844 |
| YTHDF2 | POSTAR | classical-CLIP | - |
| YTHDF2 | GEO-processed | STAMP | GSE200712 |
| YTHDF2 | GEO-processed | TRIBE | GSE200712 |
| YTHDF2 | GEO-processed | PIE-SEQ | GSE155844 |
| YTHDF3 | POSTAR | classical-CLIP | - |
| YTHDF3 | GEO-processed | STAMP | GSE200712 |
| YTHDF3 | GEO-processed | TRIBE | GSE200712 |
| YWHAE | GEO-processed | eCLIP | GSE221870 |
| YWHAG | GEO-processed | eCLIP | GSE221870 |
| YWHAG | ENCODE | eCLIP | - |
| YWHAH | GEO-processed | eCLIP | GSE221870 |
| YWHAZ | GEO-processed | eCLIP | GSE221870 |
| ZC3H11A | GEO-processed | eCLIP | GSE205536 |
| ZC3H11A | ENCODE | eCLIP | - |
| ZC3H7B | POSTAR | classical-CLIP | - |
| ZC3H8 | ENCODE | eCLIP | - |
| ZC3HAV1 | GEO-processed | eCLIP | GSE215250 |
| ZCCHC4 | GEO-reanalysis | classical-CLIP | GSE215250, GSE102336, GSE188703 |
| ZFP36 | GEO-reanalysis | classical-CLIP | GSE215250, GSE102336, GSE188703, GSE53184 |
| ZFP36L1 | GEO-reanalysis | classical-CLIP | GSE215250, GSE102336, GSE188703, GSE53184, GSE215769 |
| ZNF184 | GEO-processed | eCLIP | GSE86035 |
| ZNF622 | ENCODE | eCLIP | - |
| ZNF800 | ENCODE | eCLIP | - |
| ZRANB2 | ENCODE | eCLIP | - |
| ZRSR2 | GEO-reanalysis | classical-CLIP | GSE86035, GSE203531 |

a) For ENCODE data, two profiles were prepared for each RBP, i.e., 1) merged with default pipeline and 2) merged by taking union of the two biological replicates. b) The dataset ID recorded in POSTAR or ENCODE are not listed, as these IDs are publicly available in the corresponding databases.
